# Evolutionary tinkering of the expression of *PDF1*s suggests their joint effect on zinc tolerance and the response to pathogen attack

**DOI:** 10.3389/fpls.2014.00070

**Published:** 2014-03-11

**Authors:** Nga N. T. Nguyen, Vincent Ranwez, Denis Vile, Marie-Christine Soulié, Alia Dellagi, Dominique Expert, Françoise Gosti

**Affiliations:** ^1^Unité Mixte de Recherche, Biochimie et Physiologie Moléculaire des Plantes, Montpellier SupAgro/CNRS/INRA/Université Montpellier IIMontpellier, France; ^2^Unité Mixte de Recherche, Amélioration Génétique et Adaptation des Plantes Méditerranéennes et Tropicales, Montpellier SupAgro/CIRAD/INRAMontpellier, France; ^3^Laboratoire d'Ecophysiologie des Plantes sous Stress Environnementaux (LEPSE), UMR759 INRA/SupAgroMontpellier, France; ^4^Laboratoire des Interactions Plantes-Pathogènes, Unité Mixte de Recherche 217, Université Pierre et Marie Curie (UPMC Univ. Paris 06)Paris, France; ^5^Laboratoire des Interactions Plantes-Pathogènes, Unité Mixte de Recherche 217 INRA/AgroParisTech/UPMCParis, France

**Keywords:** defensins, zinc tolerance, MeJA, biotic and abiotic stress on plants, arabidopsis halleri, gene duplication and evolution, elementary defence and joint effect

## Abstract

Multigenic families of *Plant Defensin type 1* (*PDF1*) have been described in several species, including the model plant *Arabidopsis thaliana* as well as zinc tolerant and hyperaccumulator *A. halleri*. In *A. thaliana, PDF1* transcripts (*AtPDF1*) accumulate in response to pathogen attack following synergic activation of ethylene/jasmonate pathways. However, in *A. halleri*, *PDF1* transcripts (*AhPDF1*) are constitutively highly accumulated. Through an evolutionary approach, we investigated the possibility of *A. halleri* or *A. thaliana* species specialization in different *PDF1*s in conveying zinc tolerance and/or the response to pathogen attack *via* activation of the jasmonate (JA) signaling pathway. The accumulation of each *PDF1* from both *A. halleri* and *A. thaliana* was thus compared in response to zinc excess and MeJA application. In both species, *PDF1* paralogues were barely or not at all responsive to zinc. However, regarding the *PDF1* response to JA signaling activation, *A. thaliana* had a higher number of *PDF1*s responding to JA signaling activation. Remarkably, in *A. thaliana*, a slight but significant increase in zinc tolerance was correlated with activation of the JA signaling pathway. In addition, *A. halleri* was found to be more tolerant to the necrotrophic pathogen *Botrytis cinerea* than *A. thaliana*. Since *PDF1*s are known to be promiscuous antifungal proteins able to convey zinc tolerance, we propose, on the basis of the findings of this study, that high constitutive *PDF1* transcript accumulation in *A. halleri* is a potential way to skip the JA signaling activation step required to increase the *PDF1* transcript level in the *A. thaliana* model species. This could ultimately represent an adaptive evolutionary process that would promote a *PDF1* joint effect on both zinc tolerance and the response to pathogens in the *A. halleri* extremophile species.

## Introduction

Plants have undergone evolutionary processes allowing them to detect environmental changes and respond to various combined stress conditions, while conserving valuable resources for growth and reproduction (Atkinson and Urwin, 2012). Their responses to different stresses are highly complex and involve changes at transcriptome, cellular and physiological levels which would ultimately combine responses to both biotic and abiotic stresses. Phytohormone signaling pathway activation and a range of molecular mechanisms act together in a complex regulatory network to further orchestrate the behavior of plants in response to biotic and abiotic stresses (Fujita et al., 2006; Atkinson and Urwin, 2012). Among these, jasmonic acid (JA), an oxylipin plant hormone, is one of the most important signaling molecules coordinating plant responses to biotic and abiotic challenges (Bari and Jones, 2009; Browse, 2009; Ballare, 2011; Antico et al., 2012; Wasternack and Hause, 2013). In response to environmental stimuli, JA control a number of transcription factors regulating the expression of JA-responsive genes (Shan et al., 2007; Chico et al., 2008; Chini et al., 2009; Fonseca et al., 2009; Gfeller et al., 2010; Santino et al., 2013; Wasternack and Hause, 2013). Among these, *Plant Defensin type1* genes (*PDF1*s) are considered to be markers of JA signaling cascade activation (Memelink, 2009; Verhage et al., 2011).

Defensins are small peptide members of the antimicrobial peptide (AMP) super-family (Thomma et al., 2002; Ganz, 2003; Brown and Hancock, 2006) that are ubiquitous in the Plantae genome kingdom. They are mainly recognized for their antifungal properties, but also have multiple biological activities (Lay and Anderson, 2005; Wong et al., 2007; Carvalho and Gomes, 2011; Gachomo et al., 2012). Their specific mode of action in these different processes has yet to be clarified (Sagaram et al., 2012; De Coninck et al., 2013; Van Der Weerden et al., 2013). Defensin genes belong to multigenic families, which have been described in several species, including *Arabidopsis thaliana* (Silverstein et al., 2005, 2007). In this model species, *PDF1*s (Table 1) are usually associated with the response to pathogens and defensin *AtPDF1.2a* is considered to be a marker of the JA response (Yan et al., 2009; Pieterse et al., 2012). This defensin is inducible upon pathogen inoculation following activation of ethylene (ET) and JA signaling pathways (Penninckx et al., 1996, 1998; Manners et al., 1998; Niu et al., 2011). Expression studies were recently conducted to characterize *AtPDF1.1*, which was shown to be involved in the plant response to biotic stress (De Coninck et al., 2010). In addition, *AtPDF1.2a*-*2c*, and *AtPDF1.3* transcripts have been described for their equivalent positive response to non-host pathogens (Hiruma et al., 2011).

**Table 1:**
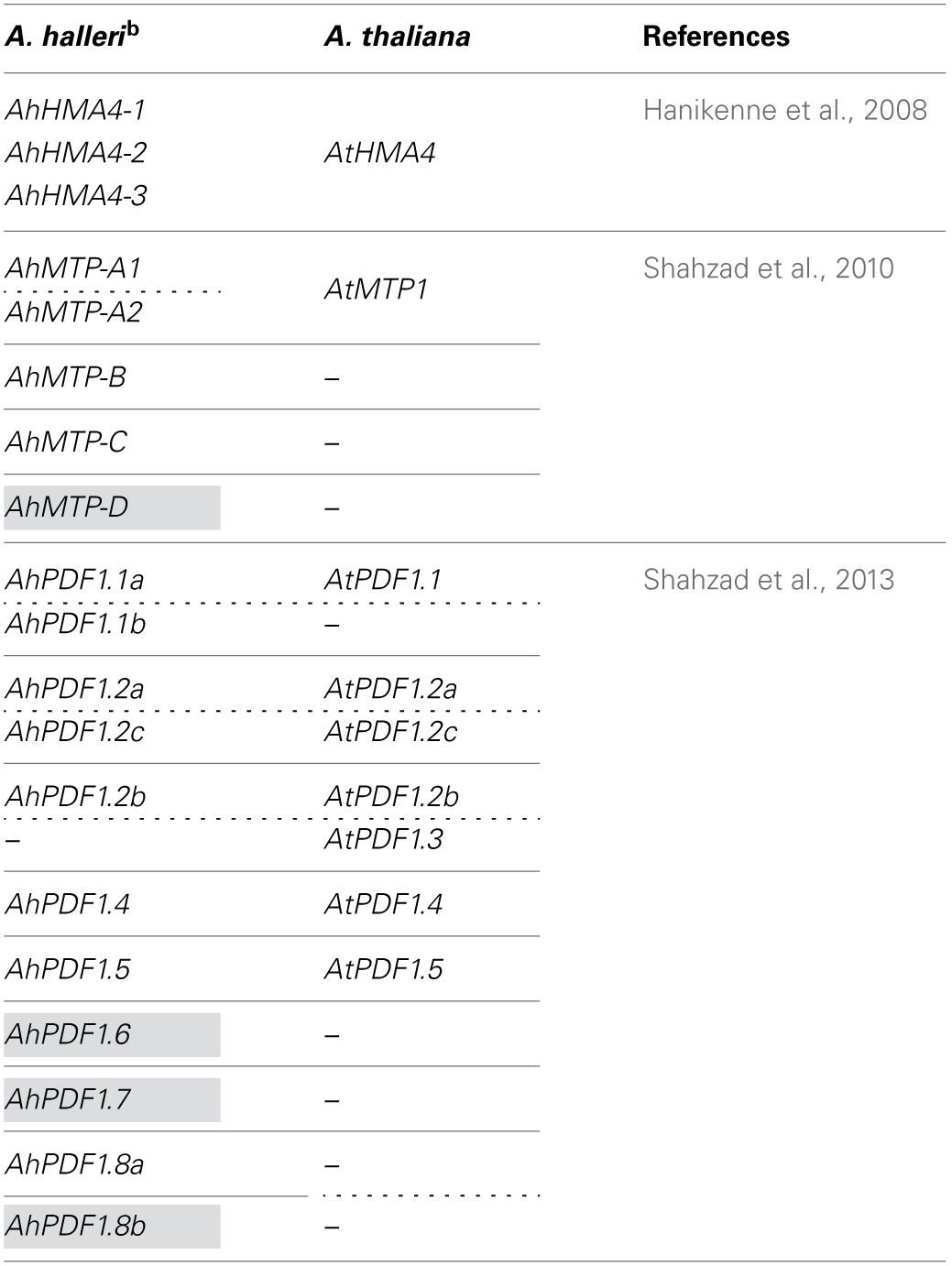
**Comparative genomic organization of some genes recognized for their high constitutive transcript accumulation in *A. halleri* as compared to *A. thaliana*^a^**.

a *Genes are classified within their family and organized according to their distribution in syntenic loci (plain lines) and to their orthologous relationship (dashed lines)*.

*^b^Gray shadowing indicates genes which were not fixed in the A. halleri population (AhMTP1-D and AhPDF1.8b) or that are likely to be non-functional (AhPDF1.6 as a pseudo-gene and AhPDF1.7 as having a premature stop codon)*.

Interestingly, the role of PDF1s in zinc tolerance has also been functionally documented in yeast and plants in studies on the extremophile species *A. halleri* (Mirouze et al., 2006). In the *Arabidopsis* genus, *A. halleri* is the only species adapted to metal contaminated soils displaying high zinc and cadmium tolerance and hyperaccumulation capacities (Clauss and Koch, 2006; Roosens et al., 2008; Kramer, 2010). *PDF1* is among several genes with high transcript accumulation in extremophile species (Hammond et al., 2006; Talke et al., 2006; Van De Mortel et al., 2006). Recent characterizations of each *PDF1* of the multigenic family showed that, in the *Arabidopsis* genus, PDF1 proteins are mostly promiscuous in their zinc tolerance and antifungal roles, i.e., the same molecule can convey zinc tolerance to yeast (*Saccharomyces cerevisiae*) while inhibiting *in vitro* fungal pathogen growth (*Fusarium oxysporum* f. sp. *melonii*) (Marques et al., 2009; Shahzad et al., 2013). Overall, these datasets indicate that *PDF1*s have a pivotal role in the plant response to both biotic (response to pathogens) and abiotic stress (here zinc excess). *PDF1* transcripts are constitutively accumulated at a much higher level in *A. halleri* than in *A. thaliana*, which is the main feature that differentiates the two species. In metal extremophile species, the evolution of hyperaccumulation is associated with drastic transcriptomic changes, which so far have been mainly shown by high constitutive transcript accumulation of metal homeostasis related genes (Talke et al., 2006; Van De Mortel et al., 2006). This high constitutive transcript accumulation of metal homeostasis related genes was noted in comparison to *A. lyrata* and *A. thaliana*, which are close phylogenetic relatives (Koch and Matschinger, 2007; Schranz et al., 2007; Beilstein et al., 2010; Roux et al., 2011). This transcriptomic modification can occur by gene amplification (Table 1) and/or in combination with high constitutive transcriptional expression (Talke et al., 2006; Van De Mortel et al., 2006; Hanikenne et al., 2008; Shahzad et al., 2010; Deinlein et al., 2012), which can be controlled by regulatory elements located in *cis* (Hanikenne et al., 2008). Note that, within the *Arabidopsis* genus, documented orthologous relationships between *PDF1*s do not favor *PDF1* specific gene expansion in the *A. halleri* lineage (Shahzad et al., 2013). The *PDF1* family should actually be considered as being evolutionarily dynamic in terms of the gain and loss of genes encoding promiscuous proteins conveying zinc tolerance and antifungal properties (Shahzad et al., 2013).

In this context, it could well be that some encoded members of the *PDF1* family have some specialized regulatory behavior. For example, some could be tailored for constitutive high transcript accumulation (as suggested for zinc tolerance) whereas others might be tailored for the pathogen attack response (as suggested for JA signaling pathway activation). However, experimental data are scarce and nothing is known so far on the *PDF1* response to JA signaling pathway activation in zinc tolerant and hyperaccumulating *A. halleri*. Conversely, in *A. thaliana*, the *PDF1* response solely to zinc excess (under axenic conditions) has not been reported. As a first indication, the present study involved an extensive characterization of the behavior of all members of the *PDF1* multigenic family in *A. thaliana* in response solely to zinc excess and in *A. halleri* in response to the application of methyl JA (MeJA), an indicator of JA signaling pathway activation (Turner et al., 2002; Cheong and Choi, 2003; Kombrink, 2012; Carvalhais et al., 2013) following pathogen attack. In both species, *PDF1*s were barely or not at all responsive to zinc, and *A. thaliana* contained a much higher number of JA-responsive *PDF1*s. Hence, *PDF1*s were mainly responsive to JA signaling activation in both species. At the functional level, a slight but significant increase in zinc tolerance was observed in *A. thaliana* following activation of the JA signaling pathway, suggesting that *PDF1*s could exert their role in zinc tolerance through *PDF1* transcript accumulation in response to this signaling pathway activation. Moreover, *A. halleri* was found to be more tolerant to *Botrytis cinerea* than *A. thaliana*. The results presented also highlight that evolutionary modification of the JA response has occurred amongst *A. thaliana* and *A. halleri* with respect to *PDF1* belonging to syntenic orthologous loci. Based on the overall data obtained in this study, we propose that the *PDF1* family was subject to an adaptive evolutionary process in the *A. halleri* extremophile species, which allowed the encoded protein to exert a “joint effect” on both zinc tolerance and the response to pathogens.

## Materials and methods

### Plant material and cultivation conditions

*Arabidopsis* seeds were obtained from the Nottingham Arabidopsis Stock Center (*A. thaliana*, Columbia accession; reference N60000) or collected at the site of Auby, France (50°24'57”N 3°03'18”E) for *A. halleri*. All experiments were conducted *in vitro* under axenic conditions in a growth chamber at 21.5°C under a long 16 h daily light cycle with 130 μmol.m^−2^.s^−1^ light intensity. Surface-sterilized seeds were germinated on standard medium containing Murashige and Skoog inorganic salts (Murashige and Skoog, 1962) at half concentration, with 1% (w/v) sucrose, 0.8% (w/v) agar and 2.5 mM (2-[N-morpholino] ethanesulfonic acid)—KOH at pH 5.7. For transcript accumulation quantification, plants were cultivated on normal media for 10 days for *A. thaliana* and 30 days for *A. halleri* to enable the plants to reach a similar developmental stage (around 6–9 leaves). Plantlets were then transferred onto standard medium supplemented with various combinations of MeJA (Sigma Aldrich, 392707) and ZnSO_4_ (Sigma Aldrich, 221376) and grown for an additional 5 days. For the zinc tolerance test, *A. thaliana* seeds (*n* = 42) were centrally sown every 5 mm according to a 3 × 3.5 cm grid pattern, as already described (Mirouze et al., 2006), on standard medium supplemented with various combinations of MeJA and ZnSO_4_.

### Quantification of transcript accumulation

Experiments were performed on plants treated or not with different concentrations of MeJA and ZnSO_4_. For each treatment, roots and shoots were harvested separately from individual plants (*n* = 6, originating from 2 biological replicates). Transcripts were quantified by quantitative RT-PCR (qRT-PCR). RNA extraction, cDNA synthesis and qRT-PCR were performed essentially as previously described (Shahzad et al., 2013). For *AhPDF1*s, the primer list, amplification efficiency and specificity of the amplified PCR products have already been described (Shahzad et al., 2013). Out of the 11 *AhPDF1*s identified, *AhPDF1.6* was not analyzed because it was described as a pseudo-gene (Shahzad et al., 2013). For each *AtPDF1*, new specific primer pairs were designed (Supplementary Table 1; Supplementary Figure 1). The specificity of primer pairs was assessed by sequencing the PCR product from the genomic DNA template (data not shown). The PCR efficiency (E) of each *AtPDF1* primer pair was determined after the analysis of 5 serial 1:10 dilutions of plasmid DNA (Supplementary Table 1). Actin was used as internal control (Shahzad et al., 2010, 2013). PCRs were performed on cDNA samples in triplicate. The qRT-PCR results were considered when the threshold cycle (*C_t_*) was below 35, as recommended in (Bustin et al., 2009). Above this value, transcripts were considered as non-detected. The *C_t_* value obtained for all experiments are listed in Supplementary Table 2. Actin relative expression levels (REL) with efficiency correction were determined as previously described (Shahzad et al., 2010, 2013) using the formula:

$$REL={\left[{\left(E\right)}^{-Ct}\right]}_{PDF1\text{ of interest}}/{\left[{\left(E\right)}^{-Ct}\right]}_{Actin},$$

where E and *C_t_* are the PCR amplification efficiency and threshold cycle, respectively, for the considered genes. The relative expression ratio (*R*), i.e., the response ratio of REL normalized to the control condition, was determined as described (Pfaffl, 2001) using the formula:

$$\begin{array}{l} R={\left(E_{PDF1\text{ of interest}}\right)}^{\Delta Ct_{PDF1\text{ of interest}}(\text{control-treatment})}\\ \;/{\left(E_{Actin}\right)}^{\Delta Ct_{Actin}(\text{control-treatment})}, \end{array}$$

where control corresponds to the transfer of plants onto media without any treatment, and treatment corresponds to the transfer of plants onto media supplemented with one of the MeJA-ZnSO_4_ combinations. Δ *C_t_* represents *C_t_* deviations of the control—treatment of the considered gene transcripts.

### Zinc tolerance assay

Shoots were harvested from pools of seedlings grown for 9 days after germination in different media (*n* ~ 20). These pools originated from 6 to 8 experimental replicates and the experiments were carried out in duplicate. Weight measurements were performed on material dried via 2 days of incubation at 80°C.

### *B. cinerea* culture and pathogenicity test

*B. cinerea* wild-type strain B0510 was grown on malt agar medium (1% of cristomalt and 1.5% of agar) at 21°C. For the pathogenicity test, *A. thaliana* and *A. halleri* leaves (*n* ~ 20 to ~40) of 6 weeks old plants were inoculated with mycelium plugs (3 mm diameter) as described in Soulie et al. (2006). Lesion surfaces were determined daily using ImageJ 1.42q. This experiment was repeated independently in triplicate.

### Promoter sequence analysis

For all *PDF1* genes from *A. thaliana* and *A. halleri* identified as previously described (Shahzad et al., 2013), a maximum 1 kb-long region before the ATG signal was considered (Supplementary File 1). Some of these upstream sequences were shorter, however, since the region contained another overlapping gene (in case of *PDF1.5*). Phylogeny inference was conducted using FFP software (Sims et al., 2009), which is an alignment-free approach based on k-mer frequencies. This alignment-free solution was preferred since, overall, *PDF1* upstream sequences were highly divergent and could not be reliably aligned. Two inferences were conducted, one with the 1 kb-upstream sequences and another with only the first 500 bp. For each inference, FFP facilities were used to conduct a bootstrap analysis with 100 replicates. Note that since FFP does not rely on sequence alignment, bootstrap resampling was not done on alignment sites but rather on distance matrix columns, as detailed in Sims et al. (2009).

Searches for *cis*-regulatory motifs were performed by considering either the promoter region corresponding to the full set of studied *PDF1*s or the one corresponding to the subset of four *PDF1*s responding positively to MeJA treatment. The subset of negatively responding *PDF1*s was not considered as it just involved a single sequence. Searches for *cis*-regulatory motifs were conducted using the TOUCAN workbench (Aerts et al., 2005). With the TOUCAN graphical interface, motifScanner software (Aerts et al., 2003) was used to search for known regulatory elements stored in PlantCare (Lescot et al., 2002). Motifs having a *P*-value < 0.5 in both 500 and 1000 bp flanking regions and appearing at least four times were considered as over-represented. Independently, specific searches were conducted with Geneious (Geneious) by manually entering motifs identified in the literature as being related to the MeJA-response.

### Statistical analysis

Comparisons of mean REL, shoot dry weights and surface lesions between treatments and/or plant species were performed using Kruskal–Wallis non-parametric tests (see Supplementary Table 3 for values regarding mean REL). Mean relative expression ratios were calculated from the mean *C_t_*, and standard errors and 95% confidence intervals were obtained using a randomization procedure of the raw *C_t_* (Pfaffl et al., 2002). All analyses were performed using R 2.15 (RCoreTeam, 2012).

## Results

### *PDF1*s are mainly evolutionarily different in their response to JA signaling pathway activation across *A. thaliana* and *A. halleri* species

**Figure 1:**
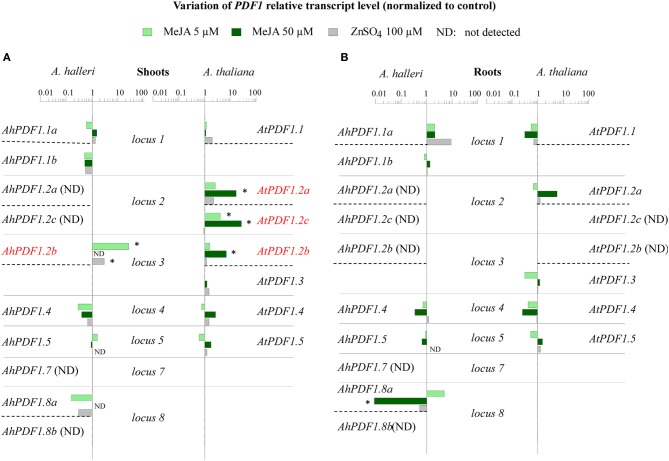
**Effect of zinc and MeJA treatments on *PDF1* transcript accumulation in shoots and roots within *A. thaliana* and *A. halleri***. The ratios of REL as normalized to actin and to control conditions are presented for each *PDF1* in shoots **(A)** and roots **(B)**. For each organ (**A,B**), the *AtPDF1* and *AhPDF1* REL ratios are organized according to orthologous loci (separated by plain lines) and the gene-to-gene orthologous relationship (indicated by dashed lines). Shoots and roots were collected separately from individual *A. halleri* (left panel) and *A. thaliana* (right panel) plants (*n* = 6) that had been transferred to and maintained for 5 days on control media or to media containing either 5 μM MeJA (light green), or 50 μM MeJA (dark green) or 100 μM ZnSO_4_ (gray). In *A. halleri*, *AhPDF1.2a*, *AhPDF1.2c*, *AhPDF1.7* and *AhPDF1.8b* transcripts were not detected in the shoots or roots. *AhPDF1.2b* transcripts were not detected in roots. In *A. thaliana*, *AtPDF1.2b* and *AtPDF1.2c* transcripts were not detected in roots. *PDF1*s shown in red had a significant transcript REL ratio in response to MeJA (see Supplementary Table 4 for bootstrapped 95% confidence interval). Stars indicate the statistical significance of the ratio (*P* < 0.05 according to bootstrapped 95% confidence intervals). ND, not detected.

In order to investigate whether any species specialization could be detected in the response of *PDF1* to zinc excess or JA signaling pathway activation, transcript quantification analyses were performed for each *PDF1* represented in both *A. halleri* and *A. thaliana*. This analysis was carried out separately in shoots and roots of plants cultivated in sterile conditions. After the plants reached a similar developmental stage, they were transferred onto media supplemented with different MeJA or zinc concentrations. Within *A. halleri* and *A. thaliana*, the *PDF1* response to zinc excess or MeJA exposure was mainly detected in shoots. Highly variable *PDF1* transcript fold changes were observed in this organ (Figure 1; see Supplementary Table 4 for bootstrapped standard errors of ratios of RELs). Upon exposure of *A. thaliana* plants to zinc, no *AtPDF1*s showed a significant transcript REL ratio response as compared to control. On the contrary, upon transfer to MeJA containing media, the expression ratio of several *AtPDF1*s significantly increased. Variations were observed for *AtPDF1.2a* (16-fold) upon transfer to 50 μM of MeJA (Figure 1). This was not surprising since this gene is commonly used as a positive marker of JA signaling pathway activation. The other *AtPDF1* that responded significantly in the shoots were *AtPDF1.2b* (7-fold for 50 μM MeJA) and *AtPDF1.2c* (4-fold and 25-fold for 5 and 50 μM MeJA, respectively). In *A. halleri*, significant ratio induction was noted for *AhPDF1.2b* in shoots upon zinc exposure (3-fold for 100 μM zinc). Surprisingly, when considering *A. halleri* species, only *AhPDF1.2b* showed a significant response ratio upon MeJA exposure (26-fold at 5 μM MeJA). Interestingly, this effect was reversed upon exposure to a higher (50 μM) MeJA concentration since transcript accumulation could not be detected under this condition (Figure 1; Supplementary Table 2). A decrease in the transcript accumulation ratio was also significantly revealed in shoots and roots for *AhPDF1.8a* treated with 50 μM MeJA (Figure 1). In summary, *A. thaliana* had a higher number of JA-responsive *PDF1*s in comparison to *A. halleri* and in both species *PDF1*s were barely or not at all responsive to zinc.

We then tried to determine if there is any evolutionary specialization between different members of the *PDF1* family by analysing the transcript quantification results in terms of orthologous and paralogous relationships within *A. thaliana* and *A. halleri* (Table 1). The response of *AtPDF1.2a* and its duplicated paralogue *AtPDF1.2c* was specific to *A. thaliana* since transcript accumulation of their syntenic orthologues in *A. halleri* (*AhPDF1.2a* and *AhPDF1.2c*) could not be detected under any of the tested physiological conditions (Figure 1; Supplementary Table 2). *AtPDF1.2b*, which was positioned at a different locus, was also one of the genes activated in response to JA signaling pathway activation. However, in that case, this property was not shared with *AtPDF1*.3, which is considered to be its duplicated paralogue (Silverstein et al., 2005, 2007). Note, however, that no gene syntenic orthologue of *AtPDF1.3* was found in *A. halleri*. Remarkably, syntenic orthologous *AtPDF1.2b* and *AhPDF1.2b* are both strongly responsive to JA signaling pathway activation. Interestingly, considering this gene, there was a marked difference between *A. thaliana* and *A. halleri*, i.e., in the latter, accumulation of *AhPDF1.2b* transcripts was no longer detected when plants were exposed to a higher MeJA concentration. A variation in this negative response was noted for *AhPDF1.8a* as its transcripts were found to constantly decrease in both shoots and roots (Figure 1).

Overall, these results suggest that the responsiveness to JA signaling pathway activation has not been systematically conserved through paralogous duplication events and has also not been systematically conserved through syntenic orthologues. In addition, some *A. halleri PDF1*s (*AhPDF1.8a*) constantly responded negatively to MeJA application, whereas others (*AhPDF1.2b*) showed a contrasting response (positive or negative response) according to the applied MeJA concentration.

### *In silico* analysis of the *PDF1* putative promoter region

**Figure 2:**
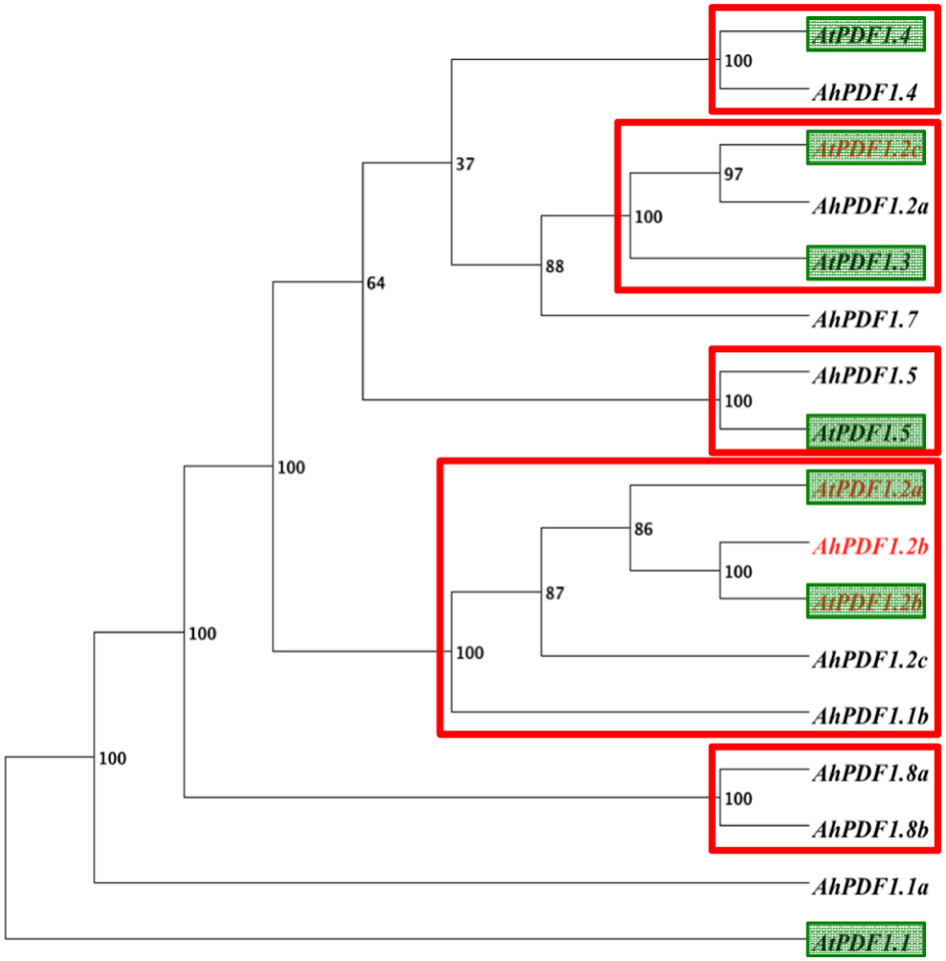
**Phylogeny of the 1 kb-upstream region of the studied *PDF1*s using an alignment free method**. Phylogeny analysis of the 1 kb-long upstream sequence for the 17 studied *PDF1* genes: 10 from *A. halleri* and 7 from *A. thaliana* (green boxes). Phylogeny inference was carried out using FFP software (Sims et al., 2009), which involves an alignment-free approach based on k-mer frequencies. The bootstrap value obtained with 100 replicates is indicated for each clade. Strongly supported clades (bootstrap support ≥ 95%) are indicated by a red rectangle. These supported clades were consistently recovered when the analysis was conducted with 500 bp upstream restriction sequences.

Besides *AtPDF1.2a*, which is characterized as a marker of JA signaling pathway activation, this study highlighted a positive MeJA-response for three additional *PDF1*s within *A. thaliana* and *A. halleri* (*AhPDF1.2b*, *AtPDF1.2b*, and *AtPDF1.2c*). In order to gain insight into these observed MeJA-response trends, nucleic sequences within all *PDF1* 1 kb-long putative promoter sequences were considered. Two distinct strategies were used: first through a study of their phylogeny to detect overall similarities between them; and second through a search of punctual motifs to detect regulatory elements they share. Phylogenetic analyses performed on 1 kb-long and on 500 bp sequences systematically grouped together (with high 100% support) the syntenic orthologues *AhPDF1.2b* and *AtPDF1.2b* (Table 1), and placed *AtPDF1.2a* as their sister group (Figure 2). Yet, in both length analyses, the putative promoter region of the fourth positive MeJA-responding gene, i.e., *AtPDF1.2c*, was always grouped with genes which were not its syntenic orthologues (Table 1; Shahzad et al., 2013) or responsive to MeJA (*AhPDF1.2a* and *AtPDF1.3* in Figure 1).

**Figure 3:**
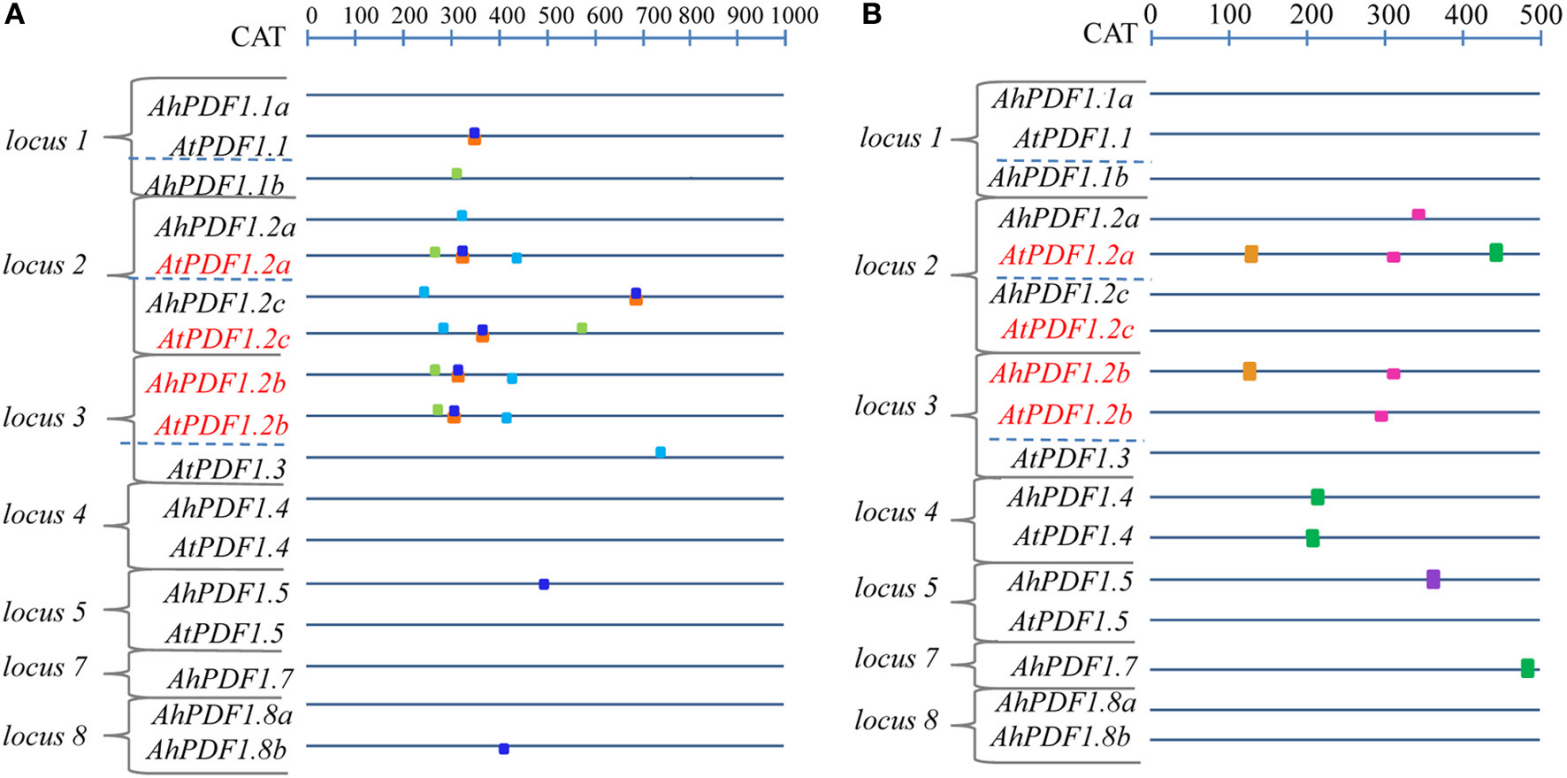
**Schematic architecture of upstream regions of *PDF1*s in *A. halleri* and *A. thaliana***. The potential promoter sequence of the 17 *A. halleri* and *A. thaliana PDF1*s are schematically represented, oriented upstream of the translation start codon from which the distance is indicated in bp on the above ruler. Red letters indicate *PDF1* sets responding positively to MeJA. **(A)** Motifs over-represented in the 1 kb-long upstream region of the *PDF1* set responding positively to MeJA are symbolized by boxes which are color-coded as follows: HV chs-Unit_1_m1 (light green), LE L-box (orange), AS GT1 (dark blue) and AT CAG (light blue). **(B)** JA-responsive motifs represented in the 500 bp upstream region of the *PDF1*s (Supplementary Table 5) are symbolized by boxes, color-coded as follows: *As*-1-type (green); G-box (light violet); G-box-like (brown); GCC-box (pink).

Since the phylogenetic sequence analysis did not reveal any grouping of *PDF1* responsiveness to MeJA, a search for punctual common *cis*-regulatory motifs was undertaken. No motif was identified as over-represented in the 500 bp upstream regions of all *PDF1*s as compared to the frequency file provided by TOUCAN for plant motifs (epd_plants_prior0.1.freq). This was not very surprising since this study clearly highlighted the heterogeneity of the *PDF1* transcript ratio in response to MeJA. In contrast, when considering the MeJA-responsive *PDF1*s (*AhPDF1.2b*, *AtPDF1.2a*, *AtPDF1.2b* and *AtPDF1.2c*) as compared to the whole set of studied *PDF1*s, a handful of motifs appeared to be significantly over-represented in the 500 bp upstream region and, most importantly, still over-represented in their 1 kb-long extension. Four motifs were revealed by these analyses (Table 2; Figure 3A). These motifs were not totally independent. Indeed, the AS GT1 motif sequence was part of the complementary LE L-box motif sequence. Consequently, the AS GT1 motif could be detected by itself (e.g., in *AhPDF1.5* and *AhPDF1.8b*), whereas the LE L-box motif was detected concomitantly with the AS GT1 motif (Figure 3A). Several of these four motifs were also present in the upstream region of other *PDF1*s, which were not characterized for their response to MeJA (Figure 3A). We specifically focused on these motifs because the following did not occur by chance: (i) their clustering in the 200–400 bp region upstream of the translation initiation site, and ii) their systematic association in the subset of *PDF1*s responsive to JA signaling pathway activation (Figure 3A). *Vegetative Storage Protein* (*VSP1* and *VSP2*) genes are also responsive to MeJA and involved in the plant response to herbivores (Hossain et al., 2011; Verhage et al., 2011). Searches conducted on the 1 kb-long upstream region of these genes revealed that two out of the four motifs were present in *VSP1* (HV chs-Unit_1_m1 and AS GT1) but none in *VSP2* (data not shown), hence these motifs were not clustered in the upstream region of *VSP* genes.

**Table 2:** **Identification of motifs over-represented in 500 bp and 1 kb-upstream regions of *PDF1*s responding to MeJA**.

**Identification**	**Sequence^a^**	**Nb Occ^b^**	***P*-value**
HV chs-Unit_1_m1	ACCTAACCCGC	4^c^	0.006
LE L-box	AGATTAACCAAC	4^d^	0.01
AT CAG motif	GAAAGGCAGAC	4^e^	0.026
AS GT1 motif	GGTTAAT	4	0.04

a *As indicated in the annotation file of MotifScaner file*.

b *Number of occurrences*.

c *Variations in these motifs are identified as “ACCTAAGCGGC” in AtPDF1.2a, AtPDF1.2b and AhPDF1.2b and “ACCAGCCCCGC” in AtPDF1.2c*.

d *Variations in these motifs are identified as “AGATTAACCAGC” in all four JA-responsive PDF1s, i.e.: AtPDF1.2a, AtPDF1.2b, AtPDF1.3, and AhPDF1.2b*.

e *Variations in these motifs are identified as “GAAGGTCAGAC” in AtPDF1.2a, AtPDF1.2b and AhPDF1.2b and “GAAAGGCTGCC” in AtPDF1.2c*.

Independently of this study, JA-responsive motifs have been identified and functionally studied *in planta* (Memelink, 2009). These motifs were sought in the studied *PDF1*s. They were found to be present at several locations and several times in different *PDF1*s originating from *A. thaliana* or *A. halleri* (Figure 3B; Supplementary Table 5). Remarkably, there were no combinations of these motifs clustered in the upstream region of the MeJA-responsive *PDF1* subset. On the other hand, no single motif was over-represented in this *PDF1* subset. Note, however, that the presence of these identified JA-responsive motifs is crucial but not sufficient since a residual response was reported to be detected when they were inactivated (Brown et al., 2003).

In summary, besides the fact that no phylogenetic grouping of promoter regions of MeJA-responsive *PDF1*s was identified, a set of over-represented motifs could be associated with these loci in the different studied species. It would thus be interesting to functionally investigate them further in order to experimentally validate their significance with respect to the *PDF1* JA-response.

### Activation of the JA signaling pathway affects zinc tolerance in *A. thaliana*

High constitutive *AhPDF1* transcript accumulation in *A. halleri* was proposed to be an evolutionary innovation co-opting promiscuous *PDF1*s for their contribution to zinc tolerance (Shahzad et al., 2013). The initial observation that the over-expression of one *PDF1* paralogue (*AhPDF1.1b*) increased zinc tolerance in *A. thaliana* plants gives functional support for this proposal (Mirouze et al., 2006). Remarkably, *PDF1* transcripts are naturally accumulated through the JA signaling pathway as part of the pathogen response.

**Figure 4:**
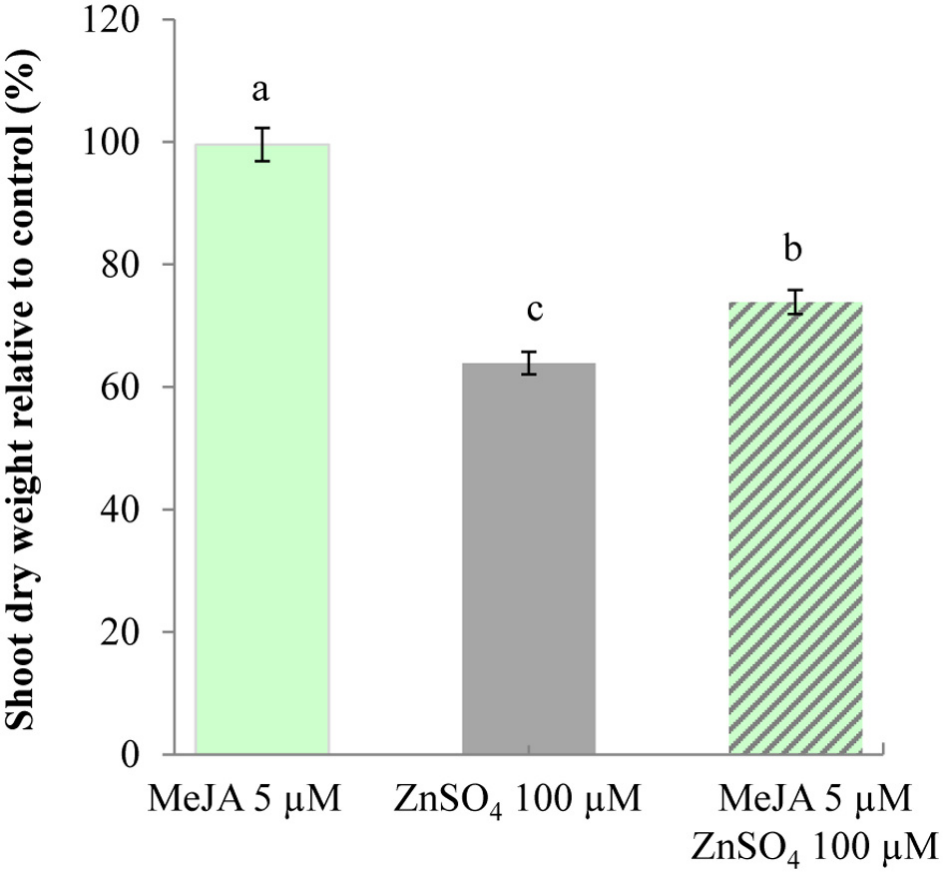
**Increased zinc tolerance of *A. thaliana* upon exposure to MeJA**. Shoots of *A. thaliana* seedlings were collected from plants (*n* = 20) 9 days following germination in control conditions or in the presence of 5 μM MeJA (light green) or 100 μM ZnSO_4_ (gray), or both 5 μM MeJA and 100 μM ZnSO_4_ (hashed). For each condition, shoot dry weights are standardized to the shoot dry weight of seedlings germinated in control conditions. Different letters indicate significant differences (*P* < 0.05) according to the Kruskal–Wallis test.

We thus investigated whether activation of this signaling pathway in *A. thaliana* could be correlated with increased zinc tolerance in this model species. Zinc tolerance assays were thus conducted by measuring the dry weight of shoots from *A. thaliana* seedlings germinated on media containing different combinations of MeJA and/or zinc (Supplementary Table 6). No significant difference was observed when measuring the dry weight of shoots from seedlings grown in control conditions or in the presence of 5 μM MeJA (Figure 4). A 36% decrease in shoot dry weight was observed for seedlings germinated in the presence of zinc, thus highlighting the sensitivity of this species. Surprisingly, there was only a 26% decrease when MeJA was added to zinc. In addition, *PDF1* transcript accumulation was affected by cultivation in the presence of MeJA, whereas accumulation of transcripts for two marker genes involved in zinc homeostasis and tolerance, i.e., *AtHMA4* and *AtMTP1* (Hanikenne et al., 2008; Shahzad et al., 2010), was not affected by MeJA treatment or by its combination with zinc treatment (Supplementary Figure 2). Activation of the JA signaling pathway through MeJA application in the media was thus responsible for this slight but significant increase in zinc tolerance.

### Increased tolerance to *B. cinerea* in *A. halleri*

**Figure 5:**
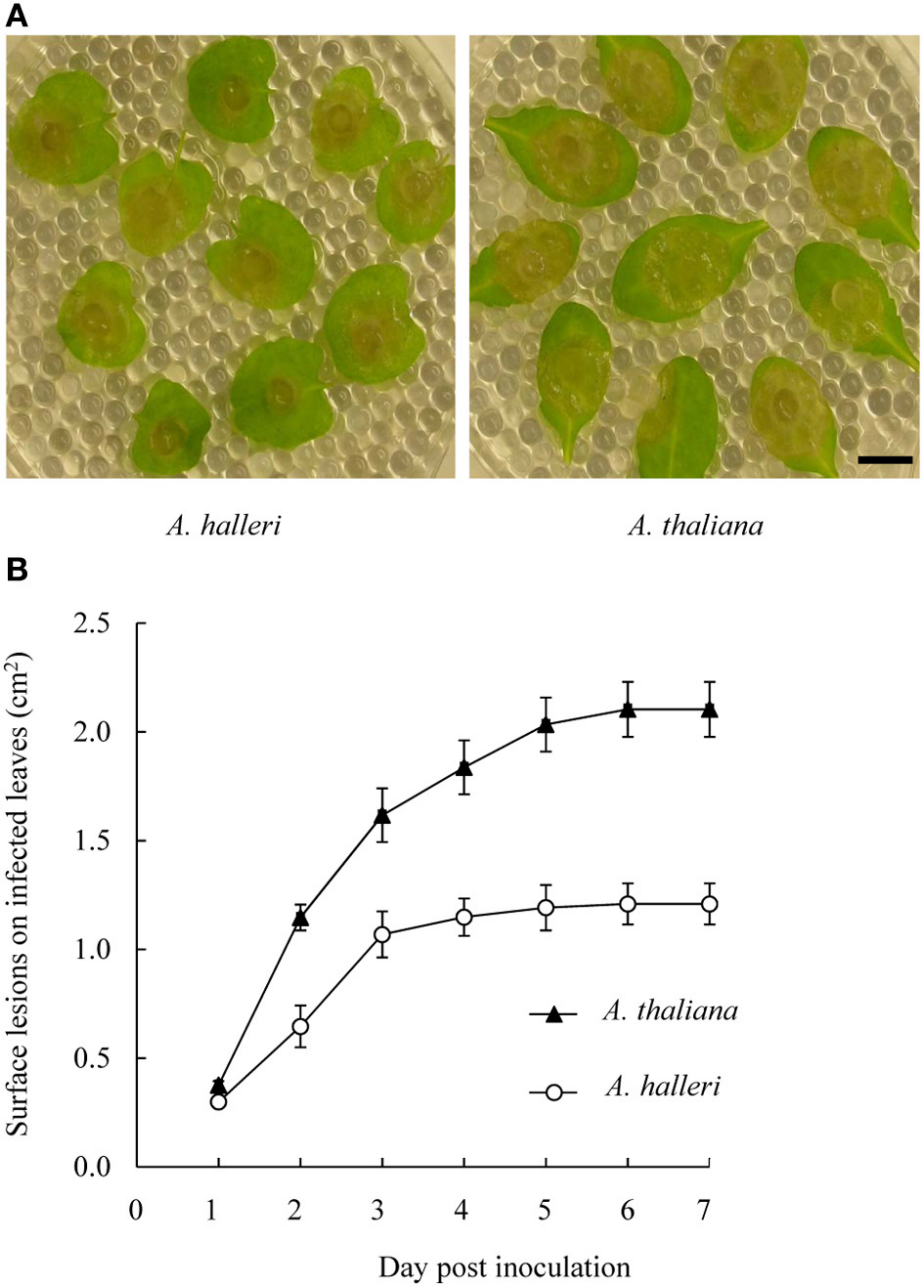
**Disease development on *A. thaliana* and *A. halleri* leaves following *B. cinerea* inoculation**. Leaves were inoculated with 3 mm diameter mycelium plugs of *B. cinerea* **(A)** Spreading lesions on *A. halleri* leaves (left) and on *A. thaliana* leaves (right) were photographed 3 days post-inoculation (dpi). Bar = 1 cm. **(B)** Surface lesions on infected leaves were measured daily up to 7 dpi. For each genotype, mean lesion surface values were determined from ~20 inoculated leaves derived from 6 plants grown in soil. Error bars represent standard deviations. A Kruskal-Wallis statistical test was performed and all *A. thaliana* measurement values showed significant differences (*p* < 0.005) with *A. halleri* measurement values from 2 dpi.

Most plant defensins display antifungal activities (Aerts et al., 2008; Carvalho and Gomes, 2011; De Coninck et al., 2013) and two major pathogen classes have been roughly identified on the basis of their “lifestyles.” Biotrophic pathogens infect living host cells and necrotrophic pathogens kill cells prior to consuming them (Oliver and Ipcho, 2004). Plant responses to biotrophic pathogens are largely mediated by salicylate signaling, while plant responses to necrotrophic pathogens appear to be mainly mediated by JA and ET. Since *PDF1* transcript induction occurs in response to JA signaling pathway activation, they are associated with plant defense against necrotrophic pathogens (Thomma et al., 1998; Glazebrook, 2005). This prompted us to investigate whether the high constitutive level of *PDF1* transcripts in *A. halleri* could be correlated with a higher level of immunity. For this purpose, we used the wide host range necrotrophic pathogen *B. cinerea* to comparatively challenge *A. halleri* and *A. thaliana* plants. Pathogenic assays were conducted by measuring the surfaces of macerating lesions during 7 days following inoculation with *B. cinerea* fungal hyphae. The results of pathogenic assays conducted following inoculation with *B. cinerea* fungal hyphae (Figure 5; Supplementary Figure 3; Supplementary Table 7) indicated that macerating surfaces in *A. halleri* were significantly reduced compared to *A. thaliana*. Hence, *A. halleri* was more tolerant to *B. cinerea* than *A. thaliana*.

## Discussion

Certain organisms are particularly remarkable in their ability to survive under metal contamination conditions that would be detrimental to other organisms. Among these, a rare class of plants, called hyperaccumulators, accumulate and detoxify extraordinarily high concentrations of metal ions in their above-ground organs (Baker, 1989; Macnair, 2003). The scientific community is now faced with the challenge of uncovering the functions involved in these extremophile plants together with the benefits and adaptive values that triggered the evolution of the metal hyperaccumulation trait (Maestri et al., 2010; Rascio and Navari-Izzo, 2011). Molecular genetics and functional studies conducted on these extremophile plant species have consistently highlighted the importance of metal homeostasis-related genes, which play a key role in driving the uptake, translocation to leaves and, finally, sequestration in vacuoles or cell walls of great amounts of heavy metals (Verbruggen et al., 2009; Kramer, 2010; Marques and Oomen, 2011; Van Der Ent et al., 2013). Now regarding the question of the benefits and adaptive values of metal hyperaccumulation, a variety of hypotheses have been put forward on the selective factors that caused the evolution of hyperaccumulation (Boyd and Martens, 1992). Of these, the “elemental defence” hypothesis has received the most supporting evidence (Boyd, 2007). This hypothesis suggests that hyperaccumulation is a self-defensive tactic because it can protect plants from some natural enemies, e.g., herbivores and pathogens (Rascio and Navari-Izzo, 2011; Boyd, 2012a,b). The chemical defence of plants from enemy attack also involves a variety of organic (secondary metabolites) compounds. The “joint effect” hypothesis is based on the idea that organic defences increase the defensive effect of metals (Boyd, 2007, 2012b). On this basis, conceptual models have been developed which might explain the evolutionary emergence of the hyperaccumulation trait (Boyd, 2012a,b; Hörger et al., 2013).

*PDF1* can shed new light on the benefits and adaptive values that triggered the evolution of metal hyperaccumulating plants because their functional promiscuity conveying zinc tolerance and antifungal properties placed them at the crossroads of the plant response to both biotic and abiotic stresses. With the aim of determining how both of these *PDF1* characteristics (zinc tolerance and antifungal properties) could be combined in zinc hyperaccumulator species, this study describes, for the first time, the comparative behavior of *PDF1* transcripts in the *A. thaliana* model species and *A. halleri* extremophile species in response to zinc excess or activation of the JA signaling pathway (as an indicator of the response to pathogen attack). The findings presented here highlighted that transcript variations in response to zinc excess and JA signaling were higher in shoots than in roots. This heterogeneity was also documented with respect to constitutive *PDF1* transcript accumulation in both *A. thaliana* and *A. halleri* (Shahzad et al., 2013). Overall, the *PDF1* characteristics presented in this study were generally in agreement with previous reports regarding the *AtPDF1* response to MeJA application (Zimmerli et al., 2004) and/or *AhPDF1* response to zinc excess (Shahzad et al., 2013). This was noteworthy since *PDF1* transcript levels are known to fluctuate and depend on culture conditions and developmental stages, etc. (https://www.genevestigator.com/gv/). Regarding abiotic stress in response to zinc excess, the present study revealed that *PDF1* transcripts were not responsive to zinc excess in *A. thaliana*. This is actually very close to the situation in *A. halleri*, where *PDF1*s are barely or not at all responsive to zinc, but instead characterized by higher constitutive transcript accumulation in comparison to *A. thaliana*, as supported by the recent detailed description of each member of this gene family (Shahzad et al., 2013). Regarding biotic stress, the present study showed that in *A. thaliana*, 3 out of 7 *PDF1*s were highly JA-responsive. Conversely, out of 11 *PDF1*s described in *A. halleri*, *AhPDF1.2b* was the only one to be JA-responsive (note, however, that exposure to a higher MeJA dosage actually reversed this response). According to these results, it would be very tempting to interpret the high constitutive *PDF1* transcript accumulation in *A. halleri* as being a means to skip the JA signaling induction characteristic noted in model species. In this context, we freely propose that there could be species specialization in promiscuous PDF1 proteins with respect to their role in zinc tolerance and the response to pathogens. In *A. thaliana*, the response to pathogens could rely on *PDF1* induction through activation of the JA signaling pathway. Conversely, in *A. halleri* hyperaccummulator and tolerant species, high constitutive *PDF1* expression would allow a joint effect on both zinc tolerance and the response to pathogens.

Comparisons of multigenic families between species should take orthologous and paralogous relationships into account since they are inextricably intertwined and constitute a framework upon which evolutionary events can be mapped (Koonin, 2005). The results presented here revealed high diversity among *PDF1* paralogues in both *A. thaliana* and *A. halleri* with respect to their response to JA signaling pathway activation, i.e., their main responsive feature. Indeed, responsiveness to JA signaling pathway activation has not been systematically conserved during paralogous duplication events (*AtPDF1.2b* and *AtPDF1.3*), nor has it been systematically conserved in syntenic orthologues (see *PDF1* present at *locus* 2). This is indicative of evolutionary differences that exist in *PDF1* expression amongst *A. thaliana* and *A. halleri* species, which could potentially be due to micro-divergences in promoter regions amongst members of this multigenic family. Indeed, this study highlighted four punctual nucleotide motifs, which so far have not been described as being associated with or involved in the JA-response. They are annotated in the PlantCare database as related also to light-responsive elements. However, this is not completely disconnected from JA signaling pathway activation since, interestingly, interplay between light and the JA-response has been described (Kazan and Manners, 2011; Svyatyna and Riemann, 2012). This is also the case for some other motifs already described in the upstream region of *AtPDF1.2a* (De Coninck et al., 2010; Zarei et al., 2011; Germain et al., 2012), which were also described to be involved in light responsiveness (Wang et al., 2011). However, this situation is not out of line with our rationale here, which was to identify a common signature within JA-responsive *PDF1* promoters between species. These motifs could thus be involved in the common JA-response of this set of *PDF1*s or could at least highlight regions to be manipulated in order to gain further insight into the regulation of this response for this set of *PDF1*s.

From an evolutionary standpoint, the existence of a multigenic family implies that genes could have different evolutionary fates (Hurles, 2004). This study also revealed the JA-negative response of some *PDF1*s in *A. halleri* (*AhPDF1.8a*) and some JA unexpected characteristics (*AhPDF1.2b*). These characteristics have never been reported so far for *PDF1*s and would deserve further exploration, particularly with several time-point measurements of *AhPDF1.2b* transcript accumulation. It could well be that these observations are indicative of the different evolutionary fates of these genes, e.g., the restriction of their response to specific physiological conditions experienced by the plant when growing in the wild. Eventually, such negative behavior could also be indicative of some “species specialization” since some concerned *AhPDF1* are located at *locus* 8, which harbors *PDF1* specifically in the extremophile species *A. halleri* (Table 1). From an evolutionary standpoint, the *PDF1* family is evolutionarily dynamic in terms of gene gains and losses, but might also be in terms of transcript regulation in response to different signals.

Plant protection by metals can occur by different modes of action, as described in (Poschenrieder et al., 2006). Metal can act as a plant stressor, which can trigger an abiotic stress response sharing many characteristics with biotic stress responses. In particular, the key factors seem to be ROS production (Mithofer et al., 2004; Fujita et al., 2006; Poschenrieder et al., 2006; Fones et al., 2013) or the ability to maintain a high level of reduced glutathione (Freeman et al., 2005). The “joint effect” might therefore be expected from both metal and these secondary metabolites (Poschenrieder et al., 2006; Boyd, 2012b; Hörger et al., 2013). However, the mode of signal transduction seems to be stress specific. Hence, activation of one of the signaling pathways might not provide broad co-resistance (see Glombitza et al., 2004 and details in Poschenrieder et al., 2006). Ultimately, hyperaccumulator plants deprived of metals might show increased susceptibility to diseases. In that case, metals might act as a remedy for metabolic defects because of their toxicity to pathogens more than to plants, (Freeman et al., 2005; Poschenrieder et al., 2006; Fones et al., 2010, 2013). In metal extremophile species, most studies on “elemental defenses” and “joint effects” have focused on responses to herbivores and relatively few studies have been conducted on responses to pathogen attacks (Fones et al., 2013). In fact, no precise data is available on *A. halleri* responses to pathogens. As a first step, we showed in this study that *A. halleri* was more tolerant than *A. thaliana*, when challenged by the necrotrophic pathogenic fungus *B. cinerea* (Figure 5; Supplementary Figure 3 and Supplementary Table 7). Since PDF1 is associated with the plant response to fungal pathogens, further studies are needed in order to go beyond this initial observation. In particular, future analyses should be focused on determining whether the disease tolerance of *A. halleri* plants is decreased when the *PDF1* transcript and/or zinc contents are decreased. Considering the PDF1 protein promiscuity, it would also be relevant to test zinc tolerance in such plants. In the same vein, it would be very interesting to investigate if high *PDF1* constitutive transcript accumulation in *A. halleri* is correlated with a high JA content. Interestingly, the presently hypothesized PDF1 “joint effect” on zinc tolerance and response to pathogens could be supported by the *A. thaliana* data presented here, which revealed a correlation between increased *PDF1* transcript accumulation following activation of the JA signaling pathway (Supplementary Figure 2) and increased zinc tolerance (Figure 4). Further in-depth genetic and physiological investigations will also now be required in *A. thaliana* to determine if and how *PDF1* are involved in zinc tolerance through their increased transcript accumulation following JA signaling pathway activation.

In conclusion, and to quote François Jacob (Jacob, 1977), we suggest that evolutionary tinkering of *PDF1* expression is an adaptive evolutionary process, leading to a *PDF1* joint effect in *A. halleri*, where “the more you defend, the more you tolerate and/or *vice versa*.” Yet, the causal links of the *PDF1* “joint effect hypothesis” remain to be assessed in both *A. thaliana* and *A. halleri* with suitable evolutionary ecological and molecular genetics approaches, since natural ecosystems are much more complex than found in laboratory experimental conditions.

### Conflict of interest statement

The authors declare that the research was conducted in the absence of any commercial or financial relationships that could be construed as a potential conflict of interest.

## Supplementary material

Supplementary Figure 1 **Location of *AtPDF1* specific primer pairs used in qRT-PCR along the cDNA aligned sequences**. *AtPDF1* cDNA sequences were obtained from The Arabidopsis Information Resource (http://arabidopsis.org/index.jsp) according to the ID given in parenthesis: *AtPDF1.1* (NM_106233), *AtPDF1.2a* (NM_123809), *AtPDF1.2b* (NM_128161), *AtPDF1.2c* (NM_123810), *AtPDF1.3* (NM_128160), *AtPDF1.4* (NM_101817) and *AtPDF1.5* (NM_10437). When *AtPDF1* cDNAs were not available (*AtPDF1.2b* and *AtPDF1.5*), cDNAs were manually predicted by slicing the genomic sequence 200 bp downstream of the stop codon. *AtPDF1* cDNAs were aligned with MUSCLE3.8.31 software (Edgar, 2004) and visualized with the BOXSHADE 3.21 software package (http://www.ch.embnet.org/software/BOX_form.html). The positions of primer pairs used for qRT-PCR were located in the alignment and color-coded according to the gene name. Start codon and stop codon were colored in light pink.

Supplementary Figure 2 **Effect of MeJA and ZnSO_4_ on transcript accumulation upon germination**. *A. thaliana* shoots were collected from pools of plants 9 days after germination in control conditions (empty boxes) or in presence of 5 μM MeJA (light green) or of both 5 μM MeJA and 100 μM ZnSO_4_ (hashed light green and gray). Transcripts could not be quantified from shoots harvested from seedlings germinated in the presence of 100 μM ZnSO_4_ due to the poor state of the seedlings. Three individual pools of plants were analyzed and transcript quantifications were averaged and expressed relative to actin. *AtPDF1.2b* and *AtPDF1.2c* transcripts were not detected in control conditions. A Kruskal-Wallis statistical test was performed and different letters indicate significant differences (*p* < 0.005). ND stands for not detected.

Supplementary Figure 3 **Disease development on *A. thaliana* and *A. halleri* leaves following *B. cinerea* inoculation for two biological repeats performed in addition to the results presented in Figure 5**. Leaves were inoculated with 3 mm diameter mycelium plugs of *B. cinerea*. For each genotype, mean lesion surface values were measured daily up to 7 dpi. For each genotype, mean lesion surface values were determined from ~20 to ~40 inoculated leaves derived from 6 plants grown in soil. Error bars represent standard deviations. A Kruskal-Wallis statistical test was performed and all *A. thaliana* measurements showed significant differences (p< 0.005) with respect to *A. halleri* measurements.

Supplementary Table 1 **Gene specific primer pairs used in quantitative RT-PCR analyses**.

Supplementary Table 2 **Threshold cycle (C_*t*_) values obtained in qRT-PCR analyses performed in transfer assays and germination assays (Excel table)**.

Supplementary Table 3 **Presence of JA-responsive motifs in the 500 bp upstream region of *PDF1*s in *A. halleri* and *A. thaliana***.

Supplementary Table 4 **Values from Kruskal Wallis tests comparing the *PDF1* relative expression levels to actin determined for *A. thaliana* and *A. halleri* plants upon transfer to MeJA or ZnSO_4_**.

Supplementary Table 5 **Median values and one-standard error equivalent and 95% confidence intervals calculated for *PDF1* REL ratios on the basis of pairwise bootstrapped C_*t*_ and primer efficiency values**.

Supplementary Table 6 **Dry weight of shoots from pools of *A. thaliana* seedlings collected 9 days following germination in control conditions or in the presence of 5 μM MeJA or 100 μM ZnSO_4_ or both 5 μM MeJA and 100 μM ZnSO_4_**.

Supplementary Table 7 **Surface lesion measurements on infected *A. thaliana* and *A. halleri* leaves following *B. cinerea* inoculation**.

Supplementary File 1 **Nucleic sequences of the putative *PDF1* promoter region used for *in silico* motif searches**.
