# Supplementary material for: Evolutionary tinkering of the expression of PDF1s suggests their joint effect on zinc tolerance and the response to pathogen attack
Source: Front Plant Sci. 2014 Mar 11;5:70. doi: 10.3389/fpls.2014.00070 (PMC3949115; doi:10.3389/fpls.2014.00070)
Supplement: Supplementary Figure 1 — Location of AtPDF1 specific primer pairs used in qRT-PCR along the cDNA aligned sequences. AtPDF1 cDNA sequences were obtained from The Arabidopsis Information Resource (http://arabidopsis.org/index.jsp) according to the ID given in parenthesis: AtPDF1.1 (NM_106233), AtPDF1.2a (NM_123809), AtPDF1.2b (NM_128161), AtPDF1.2c (NM_123810), AtPDF1.3 (NM_128160), AtPDF1.4 (NM_101817) and AtPDF1.5 (NM_10437). When AtPDF1 cDNAs were not available (AtPDF1.2b and AtPDF1.5), cDNAs were manually predicted by slicing the genomic sequence 200 bp downstream of the stop codon. AtPDF1 cDNAs were aligned with MUSCLE3.8.31 software (Edgar, 2004) and visualized with the BOXSHADE 3.21 software package (http://www.ch.embnet.org/software/BOX_form.html). The positions of primer pairs used for qRT-PCR were located in the alignment and color-coded according to the gene name. Start codon and stop codon were colored in light pink. [file Presentation1.ZIP › PROOF_Table 1.docx]

#### Table 1: Comparative genomic organization of some genes recognized for their high constitutive transcript accumulation in *A. halleri* as compared to *A. thaliana*^[[1]](#footnote-1)^

| ***A. halleri***^[[2]](#footnote-2)^ | ***A. thaliana*** | **References** |
| --- | --- | --- |
| *AhHMA4-1*  *AhHMA4-2*  *AhHMA4-3* | *AtHMA4* | (Hanikenne et al., 2008) |
|  |  |  |
| *AhMTP-A1* | *AtMTP1* | (Shahzad et al., 2010) |
| *AhMTP-A2* |  |  |
| *AhMTP-B* | *-* |  |
| *AhMTP-C* | *-* |  |
| *AhMTP-D* | *-* |  |
|  |  |  |
| *AhPDF1.1a* | *AtPDF1.1* | (Shahzad et al., 2013) |
| *AhPDF1.1b* | *-* |  |
| *AhPDF1.2a* | *AtPDF1.2a* |  |
| *AhPDF1.2c* | *AtPDF1.2c* |  |
| *AhPDF1.2b* | *AtPDF1.2b* |  |
| *-* | *AtPDF1.3* |  |
| *AhPDF1.4* | *AtPDF1.4* |  |
| *AhPDF1.5* | *AtPDF1.5* |  |
| *AhPDF1.6* | *-* |  |
| *AhPDF1.7* | *-* |  |
| *AhPDF1.8a* | *-* |  |
| *AhPDF1.8b* | *-* |  |

1. Genes are classified within their family and organized according to their distribution in syntenic loci (plain lines) and to their orthologous relationship (dashed lines). [↑](#footnote-ref-1)
2. Grey shadowing indicates genes which were not fixed in the *A. halleri* population (*AhMTP1-D* and *AhPDF1.8b*) or that are likely to be non-functional (*AhPDF1.6* as a pseudo-gene and *AhPDF1.7* as having a premature stop codon) [↑](#footnote-ref-2)
