# Supplementary material for: Evolutionary tinkering of the expression of PDF1s suggests their joint effect on zinc tolerance and the response to pathogen attack
Source: Front Plant Sci. 2014 Mar 11;5:70. doi: 10.3389/fpls.2014.00070 (PMC3949115; doi:10.3389/fpls.2014.00070)
Supplement: Supplementary Figure 1 — Location of AtPDF1 specific primer pairs used in qRT-PCR along the cDNA aligned sequences. AtPDF1 cDNA sequences were obtained from The Arabidopsis Information Resource (http://arabidopsis.org/index.jsp) according to the ID given in parenthesis: AtPDF1.1 (NM_106233), AtPDF1.2a (NM_123809), AtPDF1.2b (NM_128161), AtPDF1.2c (NM_123810), AtPDF1.3 (NM_128160), AtPDF1.4 (NM_101817) and AtPDF1.5 (NM_10437). When AtPDF1 cDNAs were not available (AtPDF1.2b and AtPDF1.5), cDNAs were manually predicted by slicing the genomic sequence 200 bp downstream of the stop codon. AtPDF1 cDNAs were aligned with MUSCLE3.8.31 software (Edgar, 2004) and visualized with the BOXSHADE 3.21 software package (http://www.ch.embnet.org/software/BOX_form.html). The positions of primer pairs used for qRT-PCR were located in the alignment and color-coded according to the gene name. Start codon and stop codon were colored in light pink. [file Presentation1.ZIP › v2_Nguyen_Author Query Form.docx]

**Author Query Form**

| **Q1** | Kindly confirm if the first name and surname of all the authors have been identified correctly in the front page and citation text. | **Yes,** first name and surname of all the authors have been identified correctly in the front page and citation text. |
| --- | --- | --- |
| **Q2** | Please ask the following authors to register with Frontiers (at https://www.frontiersin.org/Registration/Register.aspx) if they would like their names on the article abstract page and PDF to be linked to a Frontiers profile. Please ensure to register the authors before submitting the proof corrections. Non-registered authors will have the default profile image displayed by their name on the article page.  “Vincent Ranwez”  “Marie-Christine Soulie”  “Alia Dellagi”  “Dominique Expert.” | **Yes,** we ask the authors to register |
| **Q3** | If you decide to use previously published, copyrighted figures in your article, please keep in mind that it is your responsibility as author to obtain the appropriate permissions and licences and to follow any citation instructions requested by third-party rights holders. If obtaining the reproduction rights involves the payment of a fee, these charges are to be paid by the authors. | **We did not used** previously published, copyrighted figures in our article |
| **Q4** | Please provide the complete details [university (if any)] for “Laboratoire d’Ecophysiologie des Plantes sous Stress Environnementaux, Unité Mixte de Recherche 759, Montpellier, France.” | Replace by: Laboratoire d'Ecophysiologie des Plantes sous Stress Environnementaux (LEPSE), UMR759 INRA/SupAgro, F-34060 Montpellier, France. |
| **Q5** | Please reduce short running title to maximum of five words. | At present, I do not have access to the submitted running title. If the new one below is convenient to you, please replace.  "Specialization of *PDF1* response to JA-signalling in *Arabidopsis*" |
| **Q6** | Please specify the complete details for “et al. 2009b” cited here. | Fonseca et al., 2009 |
| **Q7** | Please add “Edgar, 2004” to the reference list. | Edgar RC. 2004. MUSCLE: multiple sequence alignment with high accuracy and high throughput. *Nucleic Acids Res* 32(5): 1792-1797. doi: 10.1093/nar/gkh340 |
| **Q8** | Please provide doi for the following references.  “Boyd, 2012a; Van Der Ent et al., 2012; Wang et al., 2011.” | Boyd, 2012a:  doi: 10.1016/j.plantsci.2012.06.012  Van Der Ent et al., 2012:  Correct 2012 to 2013  doi: 10.1007/s11104-012-1287-3  Wang et al., 2011:  There is no doi, but there is an ISSN number  ISSN 1861-3829 |
| **Q9** | Please provide the volume number and page range for the following references. “Fones et al., 2013; Van Der Weerden et al., 2013.” | Fones, H.N., Eyles, C.J., Bennett, M.H., Smith, J.A., and Preston, G.M. (2013). Uncoupling of reactive oxygen species accumulation and defence signalling in the metal hyperaccumulator plant Noccaea caerulescens. *New Phytol* 199**,** 916-924. doi:10.1111/nph.12354  Van Der Weerden, N.L., Bleackley, M.R., and Anderson, M.A. (2013). Properties and mechanisms of action of naturally occurring antifungal peptides. *Cell Mol Life Sci* 70**,** 3545-3570. doi: 10.1007/s00018-013-1260-1 |
| **Q10** | Please cite “Fonseca et al., 2009” inside the text. | We cite Fonseca et al, 2009, see our answer to Q6 |
| **Q11** | Please confirm whether the formatting of heading levels is fine globally. | We confirm that the formatting of heading levels is fine globally. |
| **Q12** | Lanes 138-171  Please revise presentation of Table 1:  a) The dashed lines originally present in the submitted document (attached to this message) do not appear anymore. This is preventing the good comprehension of the figure  b) in the column describing A. thaliana genes, the position of the AtHMA4 and AtMTP1 with respect to the genes presents in the A. halleri column have not been conserved. Again this can lead to a miss-understanding of this Table. |  |
| **Q13** | Lane 387  Replace "excessor" by "excess or" (missing space) |  |
| **Q14** | Lane 624  Italicize all capitals of the gene "VSP1" |  |
| **Q15** | Lanes 734-735  Suppress first sentence of Legend of Figure 4: " Dry weight of shoots from seedlings were standardized to control conditions." |  |
| **Q16** | Line 988  Replace "**Table 7"** to "**Supplementary Table 7"** |  |
| **Q17** | Line 1057  Replace "seedlingsgerminated" by "seedlings germinated" (missing space) |  |
| **Q18** | Line 1064  Replace "*A thaliana"* by "*A. thaliana"* (missing dot) |  |
