# Supplementary material for: Evolutionary tinkering of the expression of PDF1s suggests their joint effect on zinc tolerance and the response to pathogen attack
Source: Front Plant Sci. 2014 Mar 11;5:70. doi: 10.3389/fpls.2014.00070 (PMC3949115; doi:10.3389/fpls.2014.00070)
Supplement: Supplementary Figure 1 — Location of AtPDF1 specific primer pairs used in qRT-PCR along the cDNA aligned sequences. AtPDF1 cDNA sequences were obtained from The Arabidopsis Information Resource (http://arabidopsis.org/index.jsp) according to the ID given in parenthesis: AtPDF1.1 (NM_106233), AtPDF1.2a (NM_123809), AtPDF1.2b (NM_128161), AtPDF1.2c (NM_123810), AtPDF1.3 (NM_128160), AtPDF1.4 (NM_101817) and AtPDF1.5 (NM_10437). When AtPDF1 cDNAs were not available (AtPDF1.2b and AtPDF1.5), cDNAs were manually predicted by slicing the genomic sequence 200 bp downstream of the stop codon. AtPDF1 cDNAs were aligned with MUSCLE3.8.31 software (Edgar, 2004) and visualized with the BOXSHADE 3.21 software package (http://www.ch.embnet.org/software/BOX_form.html). The positions of primer pairs used for qRT-PCR were located in the alignment and color-coded according to the gene name. Start codon and stop codon were colored in light pink. [file Presentation1.ZIP › 60418_GOSTI_Author's_Proof.pdf]

frontiers

Author's Proof

Please check your proof carefully and mark all corrections in the appropriate place.

Annotate your corrections on-screen using the Adobe Reader PDF editing tools, save and upload as Author's Proof Corrections.
Login → Journal Tab → Manage Articles → Author's Proof → Select Article → Press Enter
Production Forum → Manuscript → Upload Files

If there are only a few minor corrections and query replies, these can be listed by referring to the specific line number in the proof and be directly communicated in the Discussion Forum of your article.
Login → Journal Tab → Manage Articles → Author's Proof → Select Article → Press Enter
Production Forum → Interactive Discussion → Enter Discussion Forum

Make sure to also answer all the queries thoroughly before submitting your comments, as failing to do so will cause delays.

Do not make any corrections by submitting a new manuscript file.

To ensure fast publication of your paper please return your corrections as soon as possible.

If you have any questions contact the Science Production Office.

- Ensure to proofread the entire article, including figures and tables, captions, equations, citations, and references.
- Double-check the spelling of all author names, accuracy of affiliations and addresses.
- Verify that all the special characters are displayed correctly.
- Be sure that you have obtained permission for any reprinted material.
- Carefully reply to all of the author queries to avoid any production delays.

Author Queries Form

Query No.	Details required	Author's Response
Q1	Kindly confirm if the first name and surname of all the authors have been identified correctly in the front page and citation text.	
Q2	Please ask the following authors to register with Frontiers (at https://www.frontiersin.org/Registration/Register.aspx) if they would like their names on the article abstract page and PDF to be linked to a Frontiers profile. Please ensure to register the authors before submitting the proof corrections. Non-registered authors will have the default profile image displayed by their name on the article page.<br>"Vincent Ranwez"<br>"Marie-Christine Soulie"<br>"Alia Dellagi"<br>"Dominique Expert."	
Q3	If you decide to use previously published, copyrighted figures in your article, please keep in mind that it is your responsibility as author to obtain the appropriate permissions and licences and to follow any citation instructions requested by third-party rights holders. If obtaining the reproduction rights involves the payment of a fee, these charges are to be paid by the authors.	
Q4	Please provide the complete details [university (if any)] for "Laboratoire d'Ecophysiologie des Plantes sous Stress Environnementaux, Unité Mixte de Recherche 759, Montpellier, France."	
Q5	Please reduce short running title to maximum of five words.	

Query No.	Details required	Author's Response
**Q6**	Please specify the complete details for "et al. 2009b" cited here.	
**Q7**	Please add "Edgar, 2004" to the reference list.	
**Q8**	Please provide doi for the following references.<br>"Boyd, 2012a; Van Der Ent et al., 2012; Wang et al., 2011."	
**Q9**	Please provide the volume number and page range for the following references.<br>"Fones et al., 2013; Van Der Weerden et al., 2013."	
**Q10**	Please cite "Fonseca et al., 2009" inside the text.	
**Q11**	Please confirm whether the formatting of heading levels is fine globally.	

frontiers in
PLANT SCIENCE

ORIGINAL RESEARCH ARTICLE
published: xx February 2014
doi: 10.3389/fpls.2014.00070

Evolutionary tinkering of the expression of *PDF1*s suggests their joint effect on zinc tolerance and the response to pathogen attack

Nga N. T. Nguyen[1], Vincent Ranwez[2†], Denis Vile[3†], Marie-Christine Soulié[4], Alia Dellagi[5], Dominique Expert[5] and Françoise Gosti[1]*

[1] Unité Mixte de Recherche, Biochimie et Physiologie Moléculaire des Plantes, Montpellier SupAgro/CNRS/INRA/Université Montpellier II, Montpellier, France
[2] Unité Mixte de Recherche, Amélioration Génétique et Adaptation des Plantes Méditerranéennes et Tropicales, Montpellier SupAgro/CIRAD/INRA, Montpellier, France
[3] Laboratoire d'Ecophysiologie des Plantes sous Stress Environnementaux, Unité Mixte de Recherche 759, Montpellier, France
[4] Laboratoire des Interactions Plantes-Pathogènes, Unité Mixte de Recherche 217, Université Pierre et Marie Curie (UPMC Univ. Paris 06), Paris, France
[5] Laboratoire des Interactions Plantes-Pathogènes, Unité Mixte de Recherche 217 INRA/AgroParisTech/UPMC, Paris, France

Edited by:
Cécile Nouet, University of Liège, Belgium

Reviewed by:
Cécile Nouet, University of Liège, Belgium
Barbara De Coninck, University of Leuven (KU Leuven), Belgium

***Correspondence:**
Françoise Gosti, Unité Mixte de Recherche, Biochimie et Physiologie Moléculaire des Plantes, Montpellier SupAgro/CNRS/INRA/Université Montpellier II, 2 Place Viala, F-34060 Montpellier Cedex 1, France
e-mail: gosti@supagro.inra.fr

† These authors have contributed equally to this work and are listed in alphabetical order.

Multigenic families of *Plant Defensin type 1* (*PDF1*) have been described in several species, including the model plant *Arabidopsis thaliana* as well as zinc tolerant and hyperaccumulator *A. halleri*. In *A. thaliana*, *PDF1* transcripts (*AtPDF1*) accumulate in response to pathogen attack following synergic activation of ethylene/jasmonate pathways. However, in *A. halleri*, *PDF1* transcripts (*AhPDF1*) are constitutively highly accumulated. Through an evolutionary approach, we investigated the possibility of *A. halleri* or *A. thaliana* species specialization in different *PDF1*s in conveying zinc tolerance and/or the response to pathogen attack *via* activation of the jasmonate (JA) signaling pathway. The accumulation of each *PDF1* from both *A. halleri* and *A. thaliana* was thus compared in response to zinc excess and MeJA application. In both species, *PDF1* paralogues were barely or not at all responsive to zinc. However, regarding the *PDF1* response to JA signaling activation, *A. thaliana* had a higher number of *PDF1*s responding to JA signaling activation. Remarkably, in *A. thaliana*, a slight but significant increase in zinc tolerance was correlated with activation of the JA signaling pathway. In addition, *A. halleri* was found to be more tolerant to the necrotrophic pathogen *Botrytis cinerea* than *A. thaliana*. Since *PDF1*s are known to be promiscuous antifungal proteins able to convey zinc tolerance, we propose, on the basis of the findings of this study, that high constitutive *PDF1* transcript accumulation in *A. halleri* is a potential way to skip the JA signaling activation step required to increase the *PDF1* transcript level in the *A. thaliana* model species. This could ultimately represent an adaptive evolutionary process that would promote a *PDF1* joint effect on both zinc tolerance and the response to pathogens in the *A. halleri* extremophile species.

Keywords: defensins, zinc tolerance, MeJA, biotic and abiotic stress on plants, arabidopsis halleri, gene duplication and evolution, elementary defence and joint effect

INTRODUCTION

Plants have undergone evolutionary processes allowing them to detect environmental changes and respond to various combined stress conditions, while conserving valuable resources for growth and reproduction (Atkinson and Urwin, 2012). Their responses to different stresses are highly complex and involve changes at transcriptome, cellular and physiological levels which would ultimately combine responses to both biotic and abiotic stresses. Phytohormone signaling pathway activation and a range of molecular mechanisms act together in a complex regulatory network to further orchestrate the behavior of plants in response to biotic and abiotic stresses (Fujita et al., 2006; Atkinson and Urwin, 2012). Among these, jasmonic acid (JA), an oxylipin plant hormone, is one of the most important signaling molecules coordinating plant responses to biotic and abiotic challenges (Bari and Jones, 2009; Browse, 2009; Ballare, 2011; Antico et al., 2012; Wasternack and Hause, 2013). In response to environmental stimuli, JA control a number of transcription factors regulating the expression of JA-responsive genes (Shan et al., 2007; Chico et al., 2008; Chini et al., 2009; et al., 2009a; Gfeller et al., 2010; Santino et al., 2013; Wasternack and Hause, 2013). Among these, *Plant Defensin type1* genes (*PDF1*s) are considered to be markers of JA signaling cascade activation (Memelink, 2009; Verhage et al., 2011).

Defensins are small peptide members of the antimicrobial peptide (AMP) super-family (Thomma et al., 2002; Ganz, 2003; Brown and Hancock, 2006) that are ubiquitous in the Plantae genome kingdom. They are mainly recognized for their antifungal properties, but also have multiple biological activities (Lay and Anderson, 2005; Wong et al., 2007; Carvalho and Gomes, 2011;

Gachomo et al., 2012). Their specific mode of action in these different processes has yet to be clarified (Sagaram et al., 2012; De Coninck et al., 2013; Van Der Weerden et al., 2013). Defensin genes belong to multigenic families, which have been described in several species, including *Arabidopsis thaliana* (Silverstein et al., 2005, 2007). In this model species, *PDF1*s (**Table 1**) are usually associated with the response to pathogens and defensin *AtPDF1.2a* is considered to be a marker of the JA response (Yan et al., 2009; Pieterse et al., 2012). This defensin is inducible upon pathogen inoculation following activation of ethylene (ET) and JA signaling pathways (Penninckx et al., 1996, 1998; Manners et al., 1998; Niu et al., 2011). Expression studies were recently conducted to characterize *AtPDF1.1*, which was shown to be involved in the plant response to biotic stress (De Coninck et al., 2010). In addition, *AtPDF1.2a-2c*, and *AtPDF1.3* transcripts have been described for their equivalent positive response to non-host pathogens (Hiruma et al., 2011).

Interestingly, the role of PDF1s in zinc tolerance has also been functionally documented in yeast and plants in studies on the extremophile species *A. halleri* (Mirouze et al., 2006). In the *Arabidopsis* genus, *A. halleri* is the only species adapted to metal contaminated soils displaying high zinc and cadmium

Table 1 | Comparative genomic organization of some genes recognized for their high constitutive transcript accumulation in *A. halleri* as compared to *A. thaliana*[a].

A. halleri[b]	*A. thaliana*	References
AhHMA4-1	*AtHMA4*	Hanikenne et al., 2008
AhHMA4-2		
AhHMA4-3		
AhMTP-A1	*AtMTP1*	Shahzad et al., 2010
AhMTP-A2		
AhMTP-B	–	
AhMTP-C	–	
AhMTP-D	–	
AhPDF1.1a	*AtPDF1.1*	Shahzad et al., 2013
AhPDF1.1b	–	
AhPDF1.2a	*AtPDF1.2a*	
AhPDF1.2c	*AtPDF1.2c*	
AhPDF1.2b	*AtPDF1.2b*	
–	*AtPDF1.3*	
AhPDF1.4	*AtPDF1.4*	
AhPDF1.5	*AtPDF1.5*	
AhPDF1.6	–	
AhPDF1.7	–	
AhPDF1.8a	–	
AhPDF1.8b	–	

[a] *Genes are classified within their family and organized according to their distribution in syntenic loci (plain lines) and to their orthologous relationship (dashed lines).*

[b] *Gray shadowing indicates genes which were not fixed in the A. halleri population (AhMTP1-D and AhPDF1.8b) or that are likely to be non-functional (AhPDF1.6 as a pseudo-gene and AhPDF1.7 as having a premature stop codon).*

tolerance and hyperaccumulation capacities (Clauss and Koch, 2006; Roosens et al., 2008; Kramer, 2010). *PDF1* is among several genes with high transcript accumulation in extremophile species (Hammond et al., 2006; Talke et al., 2006; Van De Mortel et al., 2006). Recent characterizations of each *PDF1* of the multigenic family showed that, in the *Arabidopsis* genus, PDF1 proteins are mostly promiscuous in their zinc tolerance and antifungal roles, i.e., the same molecule can convey zinc tolerance to yeast (*Saccharomyces cerevisiae*) while inhibiting *in vitro* fungal pathogen growth (*Fusarium oxysporum* f. sp. *melonii*) (Marques et al., 2009; Shahzad et al., 2013). Overall, these datasets indicate that *PDF1*s have a pivotal role in the plant response to both biotic (response to pathogens) and abiotic stress (here zinc excess). *PDF1* transcripts are constitutively accumulated at a much higher level in *A. halleri* than in *A. thaliana*, which is the main feature that differentiates the two species. In metal extremophile species, the evolution of hyperaccumulation is associated with drastic transcriptomic changes, which so far have been mainly shown by high constitutive transcript accumulation of metal homeostasis related genes (Talke et al., 2006; Van De Mortel et al., 2006). This high constitutive transcript accumulation of metal homeostasis related genes was noted in comparison to *A. lyrata* and *A. thaliana*, which are close phylogenetic relatives (Koch and Matschinger, 2007; Schranz et al., 2007; Beilstein et al., 2010; Roux et al., 2011). This transcriptomic modification can occur by gene amplification (**Table 1**) and/or in combination with high constitutive transcriptional expression (Talke et al., 2006; Van De Mortel et al., 2006; Hanikenne et al., 2008; Shahzad et al., 2010; Deinlein et al., 2012), which can be controlled by regulatory elements located in *cis* (Hanikenne et al., 2008). Note that, within the *Arabidopsis* genus, documented orthologous relationships between *PDF1*s do not favor *PDF1* specific gene expansion in the *A. halleri* lineage (Shahzad et al., 2013). The *PDF1* family should actually be considered as being evolutionarily dynamic in terms of the gain and loss of genes encoding promiscuous proteins conveying zinc tolerance and antifungal properties (Shahzad et al., 2013).

In this context, it could well be that some encoded members of the *PDF1* family have some specialized regulatory behavior. For example, some could be tailored for constitutive high transcript accumulation (as suggested for zinc tolerance) whereas others might be tailored for the pathogen attack response (as suggested for JA signaling pathway activation). However, experimental data are scarce and nothing is known so far on the *PDF1* response to JA signaling pathway activation in zinc tolerant and hyperaccumulating *A. halleri*. Conversely, in *A. thaliana*, the *PDF1* response solely to zinc excess (under axenic conditions) has not been reported. As a first indication, the present study involved an extensive characterization of the behavior of all members of the *PDF1* multigenic family in *A. thaliana* in response solely to zinc excess and in *A. halleri* in response to the application of methyl JA (MeJA), an indicator of JA signaling pathway activation (Turner et al., 2002; Cheong and Choi, 2003; Kombrink, 2012; Carvalhais et al., 2013) following pathogen attack. In both species, *PDF1*s were barely or not at all responsive to zinc, and *A. thaliana* contained a much higher number of JA-responsive *PDF1*s. Hence, *PDF1*s were mainly responsive to JA signaling activation in both

species. At the functional level, a slight but significant increase in zinc tolerance was observed in *A. thaliana* following activation of the JA signaling pathway, suggesting that *PDF1*s could exert their role in zinc tolerance through *PDF1* transcript accumulation in response to this signaling pathway activation. Moreover, *A. halleri* was found to be more tolerant to *Botrytis cinerea* than *A. thaliana*. The results presented also highlight that evolutionary modification of the JA response has occurred amongst *A. thaliana* and *A. halleri* with respect to *PDF1* belonging to syntenic orthologous loci. Based on the overall data obtained in this study, we propose that the *PDF1* family was subject to an adaptive evolutionary process in the *A. halleri* extremophile species, which allowed the encoded protein to exert a "joint effect" on both zinc tolerance and the response to pathogens.

MATERIALS AND METHODS

PLANT MATERIAL AND CULTIVATION CONDITIONS

Arabidopsis seeds were obtained from the Nottingham Arabidopsis Stock Center (*A. thaliana*, Columbia accession; reference N60000) or collected at the site of Auby, France (50°24′57″N 3°03′18″E) for *A. halleri*. All experiments were conducted *in vitro* under axenic conditions in a growth chamber at 21.5°C under a long 16 h daily light cycle with 130 μmol.m^{-2}.s^{-1} light intensity. Surface-sterilized seeds were germinated on standard medium containing Murashige and Skoog inorganic salts (Murashige and Skoog, 1962) at half concentration, with 1% (w/v) sucrose, 0.8% (w/v) agar and 2.5 mM (2-[N-morpholino] ethanesulfonic acid)—KOH at pH 5.7. For transcript accumulation quantification, plants were cultivated on normal media for 10 days for *A. thaliana* and 30 days for *A. halleri* to enable the plants to reach a similar developmental stage (around 6–9 leaves). Plantlets were then transferred onto standard medium supplemented with various combinations of MeJA (Sigma Aldrich, 392707) and $ZnSO_4$ (Sigma Aldrich, 221376) and grown for an additional 5 days. For the zinc tolerance test, *A. thaliana* seeds ($n = 42$) were centrally sown every 5 mm according to a 3 × 3.5 cm grid pattern, as already described (Mirouze et al., 2006), on standard medium supplemented with various combinations of MeJA and $ZnSO_4$.

QUANTIFICATION OF TRANSCRIPT ACCUMULATION

Experiments were performed on plants treated or not with different concentrations of MeJA and $ZnSO_4$. For each treatment, roots and shoots were harvested separately from individual plants ($n = 6$, originating from 2 biological replicates). Transcripts were quantified by quantitative RT-PCR (qRT-PCR). RNA extraction, cDNA synthesis and qRT-PCR were performed essentially as previously described (Shahzad et al., 2013). For *AhPDF1*s, the primer list, amplification efficiency and specificity of the amplified PCR products have already been described (Shahzad et al., 2013). Out of the 11 *AhPDF1*s identified, *AhPDF1.6* was not analyzed because it was described as a pseudo-gene (Shahzad et al., 2013). For each *AtPDF1*, new specific primer pairs were designed (**Supplementary Table 1**; **Supplementary Figure 1**). The specificity of primer pairs was assessed by sequencing the PCR product from the genomic DNA template (data not shown). The PCR efficiency (E) of each *AtPDF1* primer pair was determined after the analysis of 5 serial 1:10 dilutions of plasmid DNA (**Supplementary Table 1**). Actin was used as internal control (Shahzad et al., 2010, 2013). PCRs were performed on cDNA samples in triplicate. The qRT-PCR results were considered when the threshold cycle (C_t) was below 35, as recommended in (Bustin et al., 2009). Above this value, transcripts were considered as non-detected. The C_t value obtained for all experiments are listed in **Supplementary Table 2**. Actin relative expression levels (REL) with efficiency correction were determined as previously described (Shahzad et al., 2010, 2013) using the formula:

$$REL = [(E)^{-Ct}]_{PDF1\text{ of interest}}/[(E)^{-Ct}]_{Actin},$$

where E and C_t are the PCR amplification efficiency and threshold cycle, respectively, for the considered genes. The relative expression ratio (R), i.e., the response ratio of REL normalized to the control condition, was determined as described (Pfaffl, 2001) using the formula:

$$R = (E_{PDF1\text{ of interest}})^{\Delta Ct_{PDF1\text{ of interest}}(\text{control}-\text{treatment})} / (E_{Actin})^{\Delta Ct_{Actin}(\text{control}-\text{treatment})},$$

where control corresponds to the transfer of plants onto media without any treatment, and treatment corresponds to the transfer of plants onto media supplemented with one of the MeJA-$ZnSO_4$ combinations. ΔC_t represents C_t deviations of the control—treatment of the considered gene transcripts.

ZINC TOLERANCE ASSAY

Shoots were harvested from pools of seedlings grown for 9 days after germination in different media ($n \sim 20$). These pools originated from 6 to 8 experimental replicates and the experiments were carried out in duplicate. Weight measurements were performed on material dried via 2 days of incubation at 80°C.

B. CINEREA CULTURE AND PATHOGENICITY TEST

B. cinerea wild-type strain B0510 was grown on malt agar medium (1% of cristomalt and 1.5% of agar) at 21°C. For the pathogenicity test, *A. thaliana* and *A. halleri* leaves ($n \sim 20$ to $\sim$40) of 6 weeks old plants were inoculated with mycelium plugs (3 mm diameter) as described in Soulie et al. (2006). Lesion surfaces were determined daily using ImageJ 1.42q. This experiment was repeated independently in triplicate.

PROMOTER SEQUENCE ANALYSIS

For all *PDF1* genes from *A. thaliana* and *A. halleri* identified as previously described (Shahzad et al., 2013), a maximum 1 kb-long region before the ATG signal was considered (**Supplementary File 1**). Some of these upstream sequences were shorter, however, since the region contained another overlapping gene (in case of *PDF1.5*). Phylogeny inference was conducted using FFP software (Sims et al., 2009), which is an alignment-free approach based on k-mer frequencies. This alignment-free solution was preferred since, overall, *PDF1* upstream sequences were highly divergent and could not be reliably aligned. Two inferences were conducted, one with the 1 kb-upstream sequences and

another with only the first 500 bp. For each inference, FFP facilities were used to conduct a bootstrap analysis with 100 replicates. Note that since FFP does not rely on sequence alignment, bootstrap resampling was not done on alignment sites but rather on distance matrix columns, as detailed in Sims et al. (2009).

Searches for *cis*-regulatory motifs were performed by considering either the promoter region corresponding to the full set of studied *PDF1*s or the one corresponding to the subset of four *PDF1*s responding positively to MeJA treatment. The subset of negatively responding *PDF1*s was not considered as it just involved a single sequence. Searches for *cis*-regulatory motifs were conducted using the TOUCAN workbench (Aerts et al., 2005). With the TOUCAN graphical interface, motifScanner software (Aerts et al., 2003) was used to search for known regulatory elements stored in PlantCare (Lescot et al., 2002). Motifs having a *P*-value < 0.5 in both 500 and 1000 bp flanking regions and appearing at least four times were considered as over-represented. Independently, specific searches were conducted with Geneious (Geneious) by manually entering motifs identified in the literature as being related to the MeJA-response.

STATISTICAL ANALYSIS

Comparisons of mean REL, shoot dry weights and surface lesions between treatments and/or plant species were performed using Kruskal–Wallis non-parametric tests (see **Supplementary Table 3** for values regarding mean REL). Mean relative expression ratios were calculated from the mean C_t, and standard errors and 95% confidence intervals were obtained using a randomization procedure of the raw C_t (Pfaffl et al., 2002). All analyses were performed using R 2.15 (RCoreTeam, 2012).

RESULTS

*PDF1*s ARE MAINLY EVOLUTIONARILY DIFFERENT IN THEIR RESPONSE TO JA SIGNALING PATHWAY ACTIVATION ACROSS *A. THALIANA* AND *A. HALLERI* SPECIES

In order to investigate whether any species specialization could be detected in the response of *PDF1* to zinc excess or JA signaling pathway activation, transcript quantification analyses were performed for each *PDF1* represented in both *A. halleri* and *A. thaliana*. This analysis was carried out separately in shoots and roots of plants cultivated in sterile conditions. After the plants reached a similar developmental stage, they were transferred onto media supplemented with different MeJA or zinc concentrations. Within *A. halleri* and *A. thaliana*, the *PDF1* response to zinc excessor MeJA exposure was mainly detected in shoots. Highly variable *PDF1* transcript fold changes were observed in this organ (**Figure 1**; see **Supplementary Table 4** for bootstrapped standard errors of ratios of RELs). Upon exposure of *A. thaliana* plants to zinc, no *AtPDF1*s showed a significant transcript REL ratio response as compared to control. On the contrary, upon transfer to MeJA containing media, the expression ratio of several *AtPDF1*s significantly increased. Variations were observed for *AtPDF1.2a* (16-fold) upon transfer to 50 μM of MeJA (**Figure 1**). This was not surprising since this gene is commonly used as a positive marker of JA signaling pathway activation. The other *AtPDF1* that responded significantly in the shoots were *AtPDF1.2b* (7-fold for 50 μM MeJA) and *AtPDF1.2c* (4-fold and 25-fold for 5 and 50 μM MeJA, respectively). In *A. halleri*, significant ratio induction was noted for *AhPDF1.2b* in shoots upon zinc exposure (3-fold for 100 μM zinc). Surprisingly, when considering *A. halleri* species, only *AhPDF1.2b* showed a significant response ratio upon MeJA exposure (26-fold at 5 μM MeJA). Interestingly, this effect was reversed upon exposure to a higher (50 μM) MeJA concentration since transcript accumulation could not be detected under this condition (**Figure 1**; **Supplementary Table 2**). A decrease in the transcript accumulation ratio was also significantly revealed in shoots and roots for *AhPDF1.8a* treated with 50 μM MeJA (**Figure 1**). In summary, *A. thaliana* had a higher number of JA-responsive *PDF1*s in comparison to *A. halleri* and in both species *PDF1*s were barely or not at all responsive to zinc.

We then tried to determine if there is any evolutionary specialization between different members of the *PDF1* family by analysing the transcript quantification results in terms of orthologous and paralogous relationships within *A. thaliana* and *A. halleri* (**Table 1**). The response of *AtPDF1.2a* and its duplicated paralogue *AtPDF1.2c* was specific to *A. thaliana* since transcript accumulation of their syntenic orthologues in *A. halleri* (*AhPDF1.2a* and *AhPDF1.2c*) could not be detected under any of the tested physiological conditions (**Figure 1**; **Supplementary Table 2**). *AtPDF1.2b*, which was positioned at a different locus, was also one of the genes activated in response to JA signaling pathway activation. However, in that case, this property was not shared with *AtPDF1.3*, which is considered to be its duplicated paralogue (Silverstein et al., 2005, 2007). Note, however, that no gene syntenic orthologue of *AtPDF1.3* was found in *A. halleri*. Remarkably, syntenic orthologous *AtPDF1.2b* and *AhPDF1.2b* are both strongly responsive to JA signaling pathway activation. Interestingly, considering this gene, there was a marked difference between *A. thaliana* and *A. halleri*, i.e., in the latter, accumulation of *AhPDF1.2b* transcripts was no longer detected when plants were exposed to a higher MeJA concentration. A variation in this negative response was noted for *AhPDF1.8a* as its transcripts were found to constantly decrease in both shoots and roots (**Figure 1**).

Overall, these results suggest that the responsiveness to JA signaling pathway activation has not been systematically conserved through paralogous duplication events and has also not been systematically conserved through syntenic orthologues. In addition, some *A. halleri PDF1*s (*AhPDF1.8a*) constantly responded negatively to MeJA application, whereas others (*AhPDF1.2b*) showed a contrasting response (positive or negative response) according to the applied MeJA concentration.

IN SILICO ANALYSIS OF THE *PDF1* PUTATIVE PROMOTER REGION

Besides *AtPDF1.2a*, which is characterized as a marker of JA signaling pathway activation, this study highlighted a positive MeJA-response for three additional *PDF1*s within *A. thaliana* and *A. halleri* (*AhPDF1.2b*, *AtPDF1.2b*, and *AtPDF1.2c*). In order to gain insight into these observed MeJA-response trends, nucleic sequences within all *PDF1* 1 kb-long putative promoter sequences were considered. Two distinct strategies were used: first through a study of their phylogeny to detect overall similarities between them; and second through a search of punctual motifs to detect regulatory elements they share. Phylogenetic analyses performed

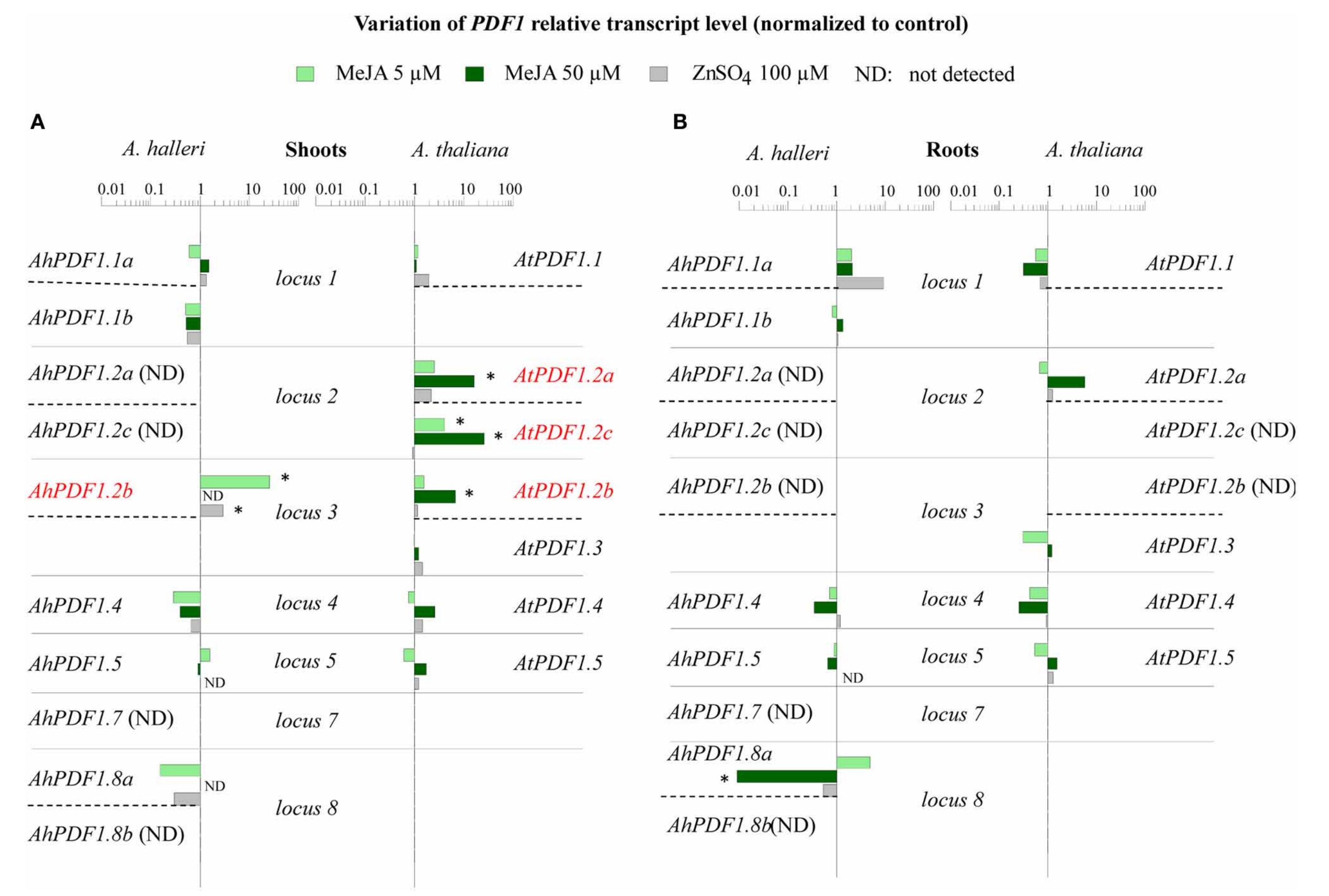

FIGURE 1 | Effect of zinc and MeJA treatments on *PDF1* transcript accumulation in shoots and roots within *A. thaliana* and *A. halleri*. The ratios of REL as normalized to actin and to control conditions are presented for each *PDF1* in shoots **(A)** and roots **(B)**. For each organ (**A,B**), the *AtPDF1* and *AhPDF1* REL ratios are organized according to orthologous loci (separated by plain lines) and the gene-to-gene orthologous relationship (indicated by dashed lines). Shoots and roots were collected separately from individual *A. halleri* (left panel) and *A. thaliana* (right panel) plants ($n = 6$) that had been transferred to and maintained for 5 days on control media or to media containing either 5 µM MeJA (light green), or 50 µM MeJA (dark green) or 100 µM $ZnSO_4$ (gray). In *A. halleri*, *AhPDF1.2a*, *AhPDF1.2c*, *AhPDF1.7* and *AhPDF1.8b* transcripts were not detected in the shoots or roots. *AhPDF1.2b* transcripts were not detected in roots. In *A. thaliana*, *AtPDF1.2b* and *AtPDF1.2c* transcripts were not detected in roots. *PDF1*s shown in red had a significant transcript REL ratio in response to MeJA (see **Supplementary Table 4** for bootstrapped 95% confidence interval). Stars indicate the statistical significance of the ratio ($P < 0.05$ according to bootstrapped 95% confidence intervals). ND, not detected.

on 1 kb-long and on 500 bp sequences systematically grouped together (with high 100% support) the syntenic orthologues *AhPDF1.2b* and *AtPDF1.2b* (**Table 1**), and placed *AtPDF1.2a* as their sister group (**Figure 2**). Yet, in both length analyses, the putative promoter region of the fourth positive MeJA-responding gene, i.e., *AtPDF1.2c*, was always grouped with genes which were not its syntenic orthologues (**Table 1**; Shahzad et al., 2013) or responsive to MeJA (*AhPDF1.2a* and *AtPDF1.3* in **Figure 1**).

Since the phylogenetic sequence analysis did not reveal any grouping of *PDF1* responsiveness to MeJA, a search for punctual common *cis*-regulatory motifs was undertaken. No motif was identified as over-represented in the 500 bp upstream regions of all *PDF1*s as compared to the frequency file provided by TOUCAN for plant motifs (epd_plants_prior0.1.freq). This was not very surprising since this study clearly highlighted the heterogeneity of the *PDF1* transcript ratio in response to MeJA. In contrast, when considering the MeJA-responsive *PDF1*s (*AhPDF1.2b*, *AtPDF1.2a*, *AtPDF1.2b* and *AtPDF1.2c*) as compared to the whole set of studied *PDF1*s, a handful of motifs appeared to be significantly over-represented in the 500 bp upstream region and, most importantly, still over-represented in their 1 kb-long extension. Four motifs were revealed by these analyses (**Table 2**; **Figure 3A**). These motifs were not totally independent. Indeed, the AS GT1 motif sequence was part of the complementary LE L-box motif sequence. Consequently, the AS GT1 motif could be detected by itself (e.g., in *AhPDF1.5* and *AhPDF1.8b*), whereas the LE L-box motif was detected concomitantly with the AS GT1 motif (**Figure 3A**). Several of these four motifs were also present in the upstream region of other *PDF1*s, which were not characterized for their response to MeJA (**Figure 3A**). We specifically focused on these motifs because the following did not occur by chance: (i) their clustering in the 200–400 bp region upstream of the translation initiation site, and ii) their systematic association in the subset of *PDF1*s responsive to JA signaling pathway activation

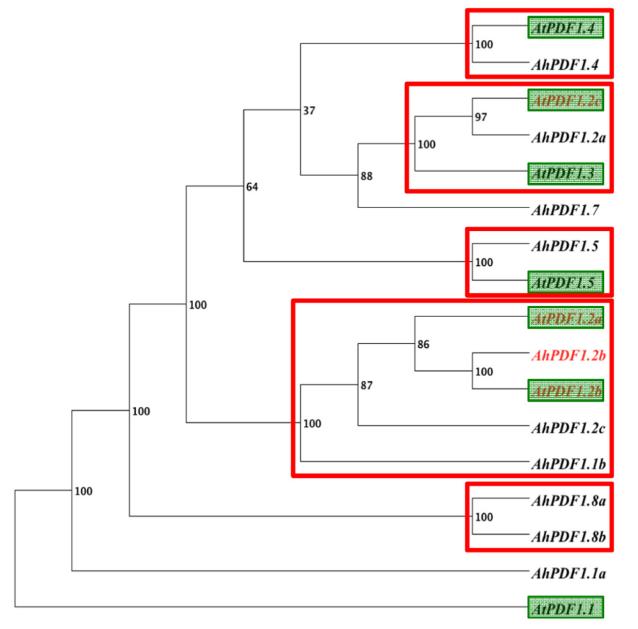

FIGURE 2 | Phylogeny of the 1 kb-upstream region of the studied *PDF1*s using an alignment free method. Phylogeny analysis of the 1 kb-long upstream sequence for the 17 studied *PDF1* genes: 10 from *A. halleri* and 7 from *A. thaliana* (green boxes). Phylogeny inference was carried out using FFP software (Sims et al., 2009), which involves an alignment-free approach based on k-mer frequencies. The bootstrap value obtained with 100 replicates is indicated for each clade. Strongly supported clades (bootstrap support ≥ 95%) are indicated by a red rectangle. These supported clades were consistently recovered when the analysis was conducted with 500 bp upstream restriction sequences.

Table 2 | Identification of motifs over-represented in 500 bp and 1 kb-upstream regions of *PDF1*s responding to MeJA.

Identification	Sequence[a]	Nb Occ[b]	*P*-value
HV chs-Unit_1_m1	ACCTAACCCGC	4[c]	0.006
LE L-box	AGATTAACCAAC	4[d]	0.01
AT CAG motif	GAAAGGCAGAC	4[e]	0.026
AS GT1 motif	GGTTAAT	4	0.04

[a] *As indicated in the annotation file of MotifScaner file.*

[b] *Number of occurrences.*

[c] *Variations in these motifs are identified as "ACCTAAGCGGC" in AtPDF1.2a, AtPDF1.2b and AhPDF1.2b and "ACCAGCCCCGC" in AtPDF1.2c.*

[d] *Variations in these motifs are identified as "AGATTAACCAGC" in all four JA-responsive PDF1s, i.e.: AtPDF1.2a, AtPDF1.2b, AtPDF1.3, and AhPDF1.2b.*

[e] *Variations in these motifs are identified as "GAAGGTCAGAC" in AtPDF1.2a, AtPDF1.2b and AhPDF1.2b and "GAAAGGCTGCC" in AtPDF1.2c.*

(**Figure 3A**). *Vegetative Storage Protein* (*VSP1* and *VSP2*) genes are also responsive to MeJA and involved in the plant response to herbivores (Hossain et al., 2011; Verhage et al., 2011). Searches conducted on the 1 kb-long upstream region of these genes revealed that two out of the four motifs were present in *V*SP1 (HV chs-Unit_1_m1 and AS GT1) but none in *VSP2* (data not shown), hence these motifs were not clustered in the upstream region of *VSP* genes.

Independently of this study, JA-responsive motifs have been identified and functionally studied *in planta* (Memelink, 2009). These motifs were sought in the studied *PDF1*s. They were found to be present at several locations and several times in different *PDF1*s originating from *A. thaliana* or *A. halleri* (**Figure 3B**; **Supplementary Table 5**). Remarkably, there were no combinations of these motifs clustered in the upstream region of the MeJA-responsive *PDF1* subset. On the other hand, no single motif was over-represented in this *PDF1* subset. Note, however, that the presence of these identified JA-responsive motifs is crucial but not sufficient since a residual response was reported to be detected when they were inactivated (Brown et al., 2003).

In summary, besides the fact that no phylogenetic grouping of promoter regions of MeJA-responsive *PDF1*s was identified, a set of over-represented motifs could be associated with these loci in the different studied species. It would thus be interesting to functionally investigate them further in order to experimentally validate their significance with respect to the *PDF1* JA-response.

ACTIVATION OF THE JA SIGNALING PATHWAY AFFECTS ZINC TOLERANCE IN *A. THALIANA*

High constitutive *AhPDF1* transcript accumulation in *A. halleri* was proposed to be an evolutionary innovation co-opting promiscuous *PDF1*s for their contribution to zinc tolerance (Shahzad et al., 2013). The initial observation that the over-expression of one *PDF1* paralogue (*AhPDF1.1b*) increased zinc tolerance in *A. thaliana* plants gives functional support for this proposal (Mirouze et al., 2006). Remarkably, *PDF1* transcripts are naturally accumulated through the JA signaling pathway as part of the pathogen response.

We thus investigated whether activation of this signaling pathway in *A. thaliana* could be correlated with increased zinc tolerance in this model species. Zinc tolerance assays were thus conducted by measuring the dry weight of shoots from *A. thaliana* seedlings germinated on media containing different combinations of MeJA and/or zinc (**Supplementary Table 6**). No significant difference was observed when measuring the dry weight of shoots from seedlings grown in control conditions or in the presence of 5 μM MeJA (**Figure 4**). A 36% decrease in shoot dry weight was observed for seedlings germinated in the presence of zinc, thus highlighting the sensitivity of this species. Surprisingly, there was only a 26% decrease when MeJA was added to zinc. In addition, *PDF1* transcript accumulation was affected by cultivation in the presence of MeJA, whereas accumulation of transcripts for two marker genes involved in zinc homeostasis and tolerance, i.e., *AtHMA4* and *AtMTP1* (Hanikenne et al., 2008; Shahzad et al., 2010), was not affected by MeJA treatment or by its combination with zinc treatment (**Supplementary Figure 2**). Activation of the JA signaling pathway through MeJA application in the media was thus responsible for this slight but significant increase in zinc tolerance.

INCREASED TOLERANCE TO *B. CINEREA* IN *A. HALLERI*

Most plant defensins display antifungal activities (Aerts et al., 2008; Carvalho and Gomes, 2011; De Coninck et al., 2013) and two major pathogen classes have been roughly identified on the basis of their "lifestyles." Biotrophic pathogens infect

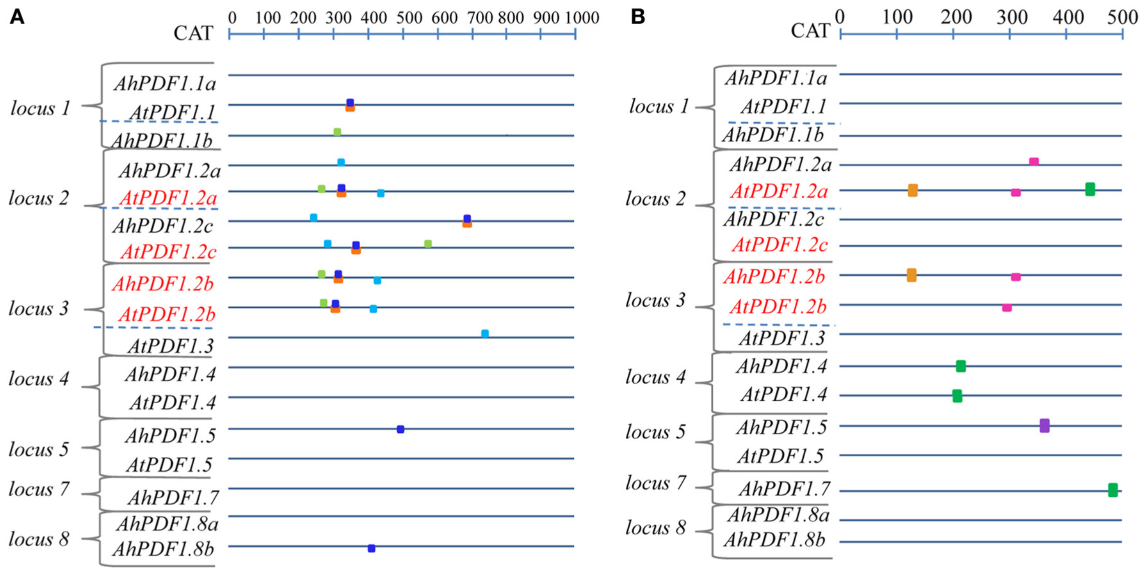

FIGURE 3 | Schematic architecture of upstream regions of *PDF1*s in *A. halleri* and *A. thaliana*. The potential promoter sequence of the 17 *A. halleri* and *A. thaliana PDF1*s are schematically represented, oriented upstream of the translation start codon from which the distance is indicated in bp on the above ruler. Red letters indicate *PDF1* sets responding positively to MeJA. **(A)** Motifs over-represented in the 1 kb-long upstream region of the *PDF1* set responding positively to MeJA are symbolized by boxes which are color-coded as follows: HV chs-Unit_1_m1 (light green), LE L-box (orange), AS GT1 (dark blue) and AT CAG (light blue). **(B)** JA-responsive motifs represented in the 500 bp upstream region of the *PDF1*s (**Supplementary Table 5**) are symbolized by boxes, color-coded as follows: *As*-1-type (green); G-box (light violet); G-box-like (brown); GCC-box (pink).

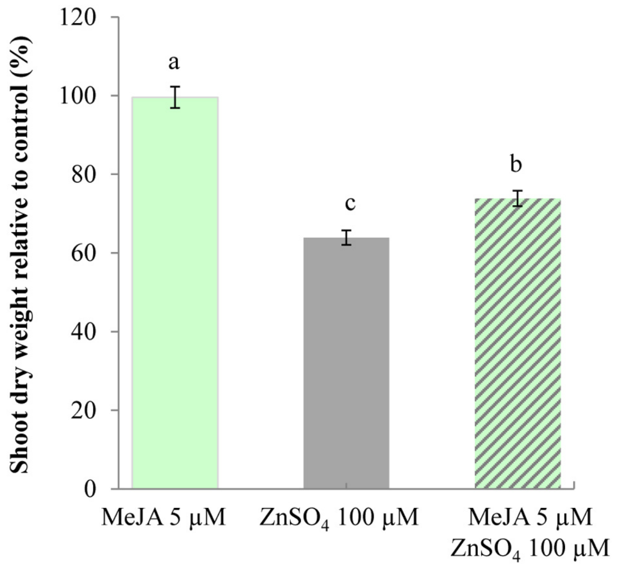

FIGURE 4 | Increased zinc tolerance of *A. thaliana* upon exposure to MeJA. Dry weight of shoots from seedlings were standardized to control conditions. Shoots of *A. thaliana* seedlings were collected from plants (n = 20) 9 days following germination in control conditions or in the presence of 5 µM MeJA (light green) or 100 µM $ZnSO_4$ (gray), or both 5 µM MeJA and 100 µM $ZnSO_4$ (hashed). For each condition, shoot dry weights are standardized to the shoot dry weight of seedlings germinated in control conditions. Different letters indicate significant differences ($P < 0.05$) according to the Kruskal–Wallis test.

living host cells and necrotrophic pathogens kill cells prior to consuming them (Oliver and Ipcho, 2004). Plant responses to biotrophic pathogens are largely mediated by salicylate signaling, while plant responses to necrotrophic pathogens appear to be mainly mediated by JA and ET. Since *PDF1* transcript induction occurs in response to JA signaling pathway activation, they are associated with plant defense against necrotrophic pathogens (Thomma et al., 1998; Glazebrook, 2005). This prompted us to investigate whether the high constitutive level of *PDF1* transcripts in *A. halleri* could be correlated with a higher level of immunity. For this purpose, we used the wide host range necrotrophic pathogen *B. cinerea* to comparatively challenge *A. halleri* and *A. thaliana* plants. Pathogenic assays were conducted by measuring the surfaces of macerating lesions during 7 days following inoculation with *B. cinerea* fungal hyphae. The results of pathogenic assays conducted following inoculation with *B. cinerea* fungal hyphae (**Figure 5**; **Supplementary Figure 3**; **supplementary Table 7**) indicated that macerating surfaces in *A. halleri* were significantly reduced compared to *A. thaliana*. Hence, *A. halleri* was more tolerant to *B. cinerea* than *A. thaliana*.

DISCUSSION

Certain organisms are particularly remarkable in their ability to survive under metal contamination conditions that would be detrimental to other organisms. Among these, a rare class of plants, called hyperaccumulators, accumulate and detoxify extraordinarily high concentrations of metal ions in their above-ground organs (Baker, 1989; Macnair, 2003). The scientific community is now faced with the challenge of uncovering

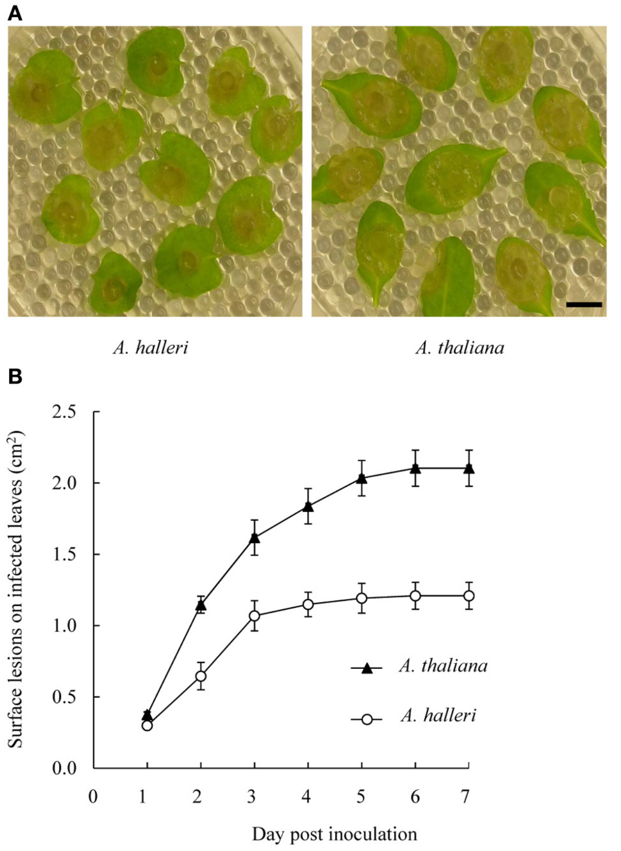

FIGURE 5 | Disease development on *A. thaliana* and *A. halleri* leaves following *B. cinerea* inoculation. Leaves were inoculated with 3 mm diameter mycelium plugs of *B. cinerea* **(A)** Spreading lesions on *A. halleri* leaves (left) and on *A. thaliana* leaves (right) were photographed 3 days post-inoculation (dpi). Bar = 1 cm. **(B)** Surface lesions on infected leaves were measured daily up to 7 dpi. For each genotype, mean lesion surface values were determined from ~20 inoculated leaves derived from 6 plants grown in soil. Error bars represent standard deviations. A Kruskal-Wallis statistical test was performed and all *A. thaliana* measurement values showed significant differences ($p < 0.005$) with *A. halleri* measurement values from 2 dpi.

the functions involved in these extremophile plants together with the benefits and adaptive values that triggered the evolution of the metal hyperaccumulation trait (Maestri et al., 2010; Rascio and Navari-Izzo, 2011). Molecular genetics and functional studies conducted on these extremophile plant species have consistently highlighted the importance of metal homeostasis-related genes, which play a key role in driving the uptake, translocation to leaves and, finally, sequestration in vacuoles or cell walls of great amounts of heavy metals (Verbruggen et al., 2009; Kramer, 2010; Marques and Oomen, 2011; Van Der Ent et al., 2012). Now regarding the question of the benefits and adaptive values of metal hyperaccumulation, a variety of hypotheses have been put forward on the selective factors that caused the evolution of hyperaccumulation (Boyd and Martens, 1992). Of these, the "elemental defence" hypothesis has received the most supporting evidence (Boyd, 2007). This hypothesis suggests that hyperaccumulation is a self-defensive tactic because it can protect plants from some natural enemies, e.g., herbivores and pathogens (Rascio and Navari-Izzo, 2011; Boyd, 2012a,b). The chemical defence of plants from enemy attack also involves a variety of organic (secondary metabolites) compounds. The "joint effect" hypothesis is based on the idea that organic defences increase the defensive effect of metals (Boyd, 2007, 2012b). On this basis, conceptual models have been developed which might explain the evolutionary emergence of the hyperaccumulation trait (Boyd, 2012a,b; Hörger et al., 2013).

PDF1 can shed new light on the benefits and adaptive values that triggered the evolution of metal hyperaccumulating plants because their functional promiscuity conveying zinc tolerance and antifungal properties placed them at the crossroads of the plant response to both biotic and abiotic stresses. With the aim of determining how both of these *PDF1* characteristics (zinc tolerance and antifungal properties) could be combined in zinc hyperaccumulator species, this study describes, for the first time, the comparative behavior of *PDF1* transcripts in the *A. thaliana* model species and *A. halleri* extremophile species in response to zinc excess or activation of the JA signaling pathway (as an indicator of the response to pathogen attack). The findings presented here highlighted that transcript variations in response to zinc excess and JA signaling were higher in shoots than in roots. This heterogeneity was also documented with respect to constitutive *PDF1* transcript accumulation in both *A. thaliana* and *A. halleri* (Shahzad et al., 2013). Overall, the *PDF1* characteristics presented in this study were generally in agreement with previous reports regarding the *AtPDF1* response to MeJA application (Zimmerli et al., 2004) and/or *AhPDF1* response to zinc excess (Shahzad et al., 2013). This was noteworthy since *PDF1* transcript levels are known to fluctuate and depend on culture conditions and developmental stages, etc. (https://www.genevestigator.com/gv/). Regarding abiotic stress in response to zinc excess, the present study revealed that *PDF1* transcripts were not responsive to zinc excess in *A. thaliana*. This is actually very close to the situation in *A. halleri*, where *PDF1*s are barely or not at all responsive to zinc, but instead characterized by higher constitutive transcript accumulation in comparison to *A. thaliana*, as supported by the recent detailed description of each member of this gene family (Shahzad et al., 2013). Regarding biotic stress, the present study showed that in *A. thaliana*, 3 out of 7 *PDF1*s were highly JA-responsive. Conversely, out of 11 *PDF1*s described in *A. halleri*, *AhPDF1.2b* was the only one to be JA-responsive (note, however, that exposure to a higher MeJA dosage actually reversed this response). According to these results, it would be very tempting to interpret the high constitutive *PDF1* transcript accumulation in *A. halleri* as being a means to skip the JA signaling induction characteristic noted in model species. In this context, we freely propose that there could be species specialization in promiscuous PDF1 proteins with respect to their role in zinc tolerance and the response to pathogens. In *A. thaliana*, the response to pathogens could rely on *PDF1* induction through activation of the JA signaling pathway. Conversely, in *A. halleri* hyperaccummulator and tolerant species, high constitutive *PDF1* expression would allow a joint effect on both zinc tolerance and the response to pathogens.

Comparisons of multigenic families between species should take orthologous and paralogous relationships into account since

they are inextricably intertwined and constitute a framework upon which evolutionary events can be mapped (Koonin, 2005). The results presented here revealed high diversity among *PDF1* paralogues in both *A. thaliana* and *A. halleri* with respect to their response to JA signaling pathway activation, i.e., their main responsive feature. Indeed, responsiveness to JA signaling pathway activation has not been systematically conserved during paralogous duplication events (*AtPDF1.2b* and *AtPDF1.3*), nor has it been systematically conserved in syntenic orthologues (see *PDF1* present at *locus* 2). This is indicative of evolutionary differences that exist in *PDF1* expression amongst *A. thaliana* and *A. halleri* species, which could potentially be due to micro-divergences in promoter regions amongst members of this multigenic family. Indeed, this study highlighted four punctual nucleotide motifs, which so far have not been described as being associated with or involved in the JA-response. They are annotated in the PlantCare database as related also to light-responsive elements. However, this is not completely disconnected from JA signaling pathway activation since, interestingly, interplay between light and the JA-response has been described (Kazan and Manners, 2011; Svyatyna and Riemann, 2012). This is also the case for some other motifs already described in the upstream region of *AtPDF1.2a* (De Coninck et al., 2010; Zarei et al., 2011; Germain et al., 2012), which were also described to be involved in light responsiveness (Wang et al., 2011). However, this situation is not out of line with our rationale here, which was to identify a common signature within JA-responsive *PDF1* promoters between species. These motifs could thus be involved in the common JA-response of this set of *PDF1*s or could at least highlight regions to be manipulated in order to gain further insight into the regulation of this response for this set of *PDF1*s.

From an evolutionary standpoint, the existence of a multigenic family implies that genes could have different evolutionary fates (Hurles, 2004). This study also revealed the JA-negative response of some *PDF1*s in *A. halleri* (*AhPDF1.8a*) and some JA unexpected characteristics (*AhPDF1.2b*). These characteristics have never been reported so far for *PDF1*s and would deserve further exploration, particularly with several time-point measurements of *AhPDF1.2b* transcript accumulation. It could well be that these observations are indicative of the different evolutionary fates of these genes, e.g., the restriction of their response to specific physiological conditions experienced by the plant when growing in the wild. Eventually, such negative behavior could also be indicative of some "species specialization" since some concerned *AhPDF1* are located at *locus* 8, which harbors *PDF1* specifically in the extremophile species *A. halleri* (**Table 1**). From an evolutionary standpoint, the *PDF1* family is evolutionarily dynamic in terms of gene gains and losses, but might also be in terms of transcript regulation in response to different signals.

Plant protection by metals can occur by different modes of action, as described in (Poschenrieder et al., 2006). Metal can act as a plant stressor, which can trigger an abiotic stress response sharing many characteristics with biotic stress responses. In particular, the key factors seem to be ROS production (Mithofer et al., 2004; Fujita et al., 2006; Poschenrieder et al., 2006; Fones et al., 2013) or the ability to maintain a high level of reduced glutathione (Freeman et al., 2005). The "joint effect" might therefore be expected from both metal and these secondary metabolites (Poschenrieder et al., 2006; Boyd, 2012b; Hörger et al., 2013). However, the mode of signal transduction seems to be stress specific. Hence, activation of one of the signaling pathways might not provide broad co-resistance (see Glombitza et al., 2004 and details in Poschenrieder et al., 2006). Ultimately, hyperaccumulator plants deprived of metals might show increased susceptibility to diseases. In that case, metals might act as a remedy for metabolic defects because of their toxicity to pathogens more than to plants, (Freeman et al., 2005; Poschenrieder et al., 2006; Fones et al., 2010, 2013). In metal extremophile species, most studies on "elemental defenses" and "joint effects" have focused on responses to herbivores and relatively few studies have been conducted on responses to pathogen attacks (Fones et al., 2013). In fact, no precise data is available on *A. halleri* responses to pathogens. As a first step, we showed in this study that *A. halleri* was more tolerant than *A. thaliana*, when challenged by the necrotrophic pathogenic fungus *B. cinerea* (**Figure 5**; **Supplementary Figure 3** and **Table 7**). Since PDF1 is associated with the plant response to fungal pathogens, further studies are needed in order to go beyond this initial observation. In particular, future analyses should be focused on determining whether the disease tolerance of *A. halleri* plants is decreased when the *PDF1* transcript and/or zinc contents are decreased. Considering the PDF1 protein promiscuity, it would also be relevant to test zinc tolerance in such plants. In the same vein, it would be very interesting to investigate if high *PDF1* constitutive transcript accumulation in *A. halleri* is correlated with a high JA content. Interestingly, the presently hypothesized PDF1 "joint effect" on zinc tolerance and response to pathogens could be supported by the *A. thaliana* data presented here, which revealed a correlation between increased *PDF1* transcript accumulation following activation of the JA signaling pathway (**Supplementary Figure 2**) and increased zinc tolerance (**Figure 4**). Further in-depth genetic and physiological investigations will also now be required in *A. thaliana* to determine if and how *PDF1* are involved in zinc tolerance through their increased transcript accumulation following JA signaling pathway activation.

In conclusion, and to quote François Jacob (Jacob, 1977), we suggest that evolutionary tinkering of *PDF1* expression is an adaptive evolutionary process, leading to a *PDF1* joint effect in *A. halleri*, where "the more you defend, the more you tolerate and/or *vice versa*." Yet, the causal links of the *PDF1* "joint effect hypothesis" remain to be assessed in both *A. thaliana* and *A. halleri* with suitable evolutionary ecological and molecular genetics approaches, since natural ecosystems are much more complex than found in laboratory experimental conditions.

ACKNOWLEDGMENTS

Thi Ngoc Nga Nguyen is supported by a Ph.D. scholarship from the University of Science and Technology of Hanoi. We are thankful to Dr. Pierre Saumitou-Laprade for kindly providing us with *A. halleri* seeds collected at the site of Auby, France. We are greatly indebted to Dr. Christian Dubos for critical advice regarding *in silico* promoter analysis. We are grateful to Drs. Hatem Rouached and Zaigham Shahzad for helpful comments on the manuscript.

We acknowledge Pr Pierre Berthomieu for initial input and constructive comments on this work. We deeply acknowledge each of the referees for their helpful comments and constructive suggestions, which greatly improved the presentation of this article.

SUPPLEMENTARY MATERIAL

The Supplementary Material for this article can be found online at: http://www.frontiersin.org/journal/10.3389/fpls.2014.00070/abstract

Supplementary Figure 1. | Location of *AtPDF1* specific primer pairs used in qRT-PCR along the cDNA aligned sequences. *AtPDF1* cDNA sequences were obtained from The Arabidopsis Information Resource (http://arabidopsis.org/index.jsp) according to the ID given in parenthesis: *AtPDF1.1* (NM_106233), *AtPDF1.2a* (NM_123809), *AtPDF1.2b* (NM_128161), *AtPDF1.2c* (NM_123810), *AtPDF1.3* (NM_128160), *AtPDF1.4* (NM_101817) and *AtPDF1.5* (NM_10437). When *AtPDF1* cDNAs were not available (*AtPDF1.2b* and *AtPDF1.5*), cDNAs were manually predicted by slicing the genomic sequence 200 bp downstream of the stop codon. *AtPDF1* cDNAs were aligned with MUSCLE3.8.31 software (Edgar, 2004) and visualized with the BOXSHADE 3.21 software package (http://www.ch.embnet.org/software/BOX_form.html). The positions of primer pairs used for qRT-PCR were located in the alignment and color-coded according to the gene name. Start codon and stop codon were colored in light pink.

Supplementary Figure 2. | Effect of MeJA and $ZnSO_4$ on transcript accumulation upon germination. *A. thaliana* shoots were collected from pools of plants 9 days after germination in control conditions (empty boxes) or in presence of 5 μM MeJA (light green) or of both 5 μM MeJA and 100 μM $ZnSO_4$ (hashed light green and gray). Transcripts could not be quantified from shoots harvested from seedlingsgerminated in the presence of 100 μM $ZnSO_4$ due to the poor state of the seedlings. Three individual pools of plants were analyzed and transcript quantifications were averaged and expressed relative to actin. *AtPDF1.2b* and *AtPDF1.2c* transcripts were not detected in control conditions. A Kruskal-Wallis statistical test was performed and different letters indicate significant differences ($p < 0.005$). ND stands for not detected.

Supplementary Figure 3. | Disease development on *A thaliana* and *A. halleri* leaves following *B. cinerea* inoculation for two biological repeats performed in addition to the results presented in Figure 5. Leaves were inoculated with 3 mm diameter mycelium plugs of *B. cinerea*. For each genotype, mean lesion surface values were measured daily up to 7 dpi. For each genotype, mean lesion surface values were determined from ~20 to ~40 inoculated leaves derived from 6 plants grown in soil. Error bars represent standard deviations. A Kruskal-Wallis statistical test was performed and all *A. thaliana* measurements showed significant differences ($p < 0.005$) with respect to *A. halleri* measurements.

Supplementary Table 1. | Gene specific primer pairs used in quantitative RT-PCR analyses.

Supplementary Table 2. | Threshold cycle (C_t) values obtained in qRT-PCR analyses performed in transfer assays and germination assays (Excel table).

Supplementary Table 3. | Presence of JA-responsive motifs in the 500 bp upstream region of *PDF1*s in *A. halleri* and *A. thaliana*.

Supplementary Table 4. | Values from Kruskal Wallis tests comparing the *PDF1* relative expression levels to actin determined for *A. thaliana* and *A. halleri* plants upon transfer to MeJA or $ZnSO_4$.

Supplementary Table 5. | Median values and one-standard error equivalent and 95% confidence intervals calculated for *PDF1* REL ratios on the basis of pairwise bootstrapped C_t and primer efficiency values.

Supplementary Table 6. | Dry weight of shoots from pools of *A. thaliana* seedlings collected 9 days following germination in control conditions or in the presence of 5 μM MeJA or 100 μM $ZnSO_4$ or both 5 μM MeJA and 100 μM $ZnSO_4$.

Supplementary Table 7. | Surface lesion measurements on infected *A. thaliana* and *A. halleri* leaves following *B. cinerea* inoculation.

Supplementary File 1. | Nucleic sequences of the putative *PDF1* promoter region used for *in silico* motif searches.

REFERENCES

Aerts, A. M., Francois, I. E., Cammue, B. P., and Thevissen, K. (2008). The mode of antifungal action of plant, insect and human defensins. *Cell. Mol. Life Sci.* 65, 2069–2079. doi: 10.1007/s00018-008-8035-0

Aerts, S., Thijs, G., Coessens, B., Staes, M., Moreau, Y., and De Moor, B. (2003). Toucan: deciphering the *cis*-regulatory logic of coregulated genes. *Nucleic Acids Res.* 31, 1753–1764. doi: 10.1093/nar/gkg268

Aerts, S., Van Loo, P., Thijs, G., Mayer, H., De Martin, R., Moreau, Y., et al. (2005). TOUCAN 2: the all-inclusive open source workbench for regulatory sequence analysis. *Nucleic Acids Res.* 33, W393–W396. doi: 10.1093/nar/gki354

Antico, C. J., Colon, C., Banks, T., and Ramonell, K. M. (2012). Insights into the role of jasmonic acid-mediated defenses against necrotrophic and biotrophic fungal pathogens. *Front. Biol.* 7:1. doi: 10.1007/s11515-011-1171-1

Atkinson, N. J., and Urwin, P. E. (2012). The interaction of plant biotic and abiotic stresses: from genes to the field. *J. Exp. Bot.* 63, 3523–3543. doi: 10.1093/jxb/ers100

Baker, A. J. M. (1989). Terrestrial higher plants which hyper-accumulate metallic elements- a review of their distribution, ecology and phytochemistry. *Biorecovery* 1, 81–126.

Ballare, C. L. (2011). Jasmonate-induced defenses: a tale of intelligence, collaborators and rascals. *Trends Plant Sci.* 16, 249–257. doi: 10.1016/j.tplants.2010.12.001

Bari, R., and Jones, J. D. (2009). Role of plant hormones in plant defence responses. *Plant Mol. Biol.* 69, 473–488. doi: 10.1007/s11103-008-9435-0

Beilstein, M. A., Nagalingum, N. S., Clements, M. D., Manchester, S. R., and Mathews, S. (2010). Dated molecular phylogenies indicate a Miocene origin for *Arabidopsis thaliana*. *Proc. Natl. Acad. Sci. U.S.A.* 107, 18724–18728. doi: 10.1073/pnas.0909766107

Boyd, R. S. (2007). The defense hypothesis of elemental hyperaccumulation: status, challenges and new directions. *Plant Soil* 293, 153–176. doi: 10.1007/s11104-007-9240-6

Boyd, R. S. (2012a). Elemental defenses of plants by metals. *Nat. Educ. Knowl.* 3, 57.

Boyd, R. S. (2012b). Plant defense using toxic inorganic ions: conceptual models of the defensive enhancement and joint effects hypotheses. *Plant Sci.* 195, 88–95. doi: 10.1016/j.plantsci.2012.06.012

Boyd, R. S., and Martens, S. N. (1992). "The raison d'être for metal hyperaccumulation by plants," in *The Vegetation of Ultramafic (Serpentine) Soils*, eds A. J. M. Baker, J. Proctor and R. D. Reeves (Andover: Intercept Limited), 279–289.

Brown, K. L., and Hancock, R. E. (2006). Cationic host defense (antimicrobial) peptides. *Curr. Opin. Immunol.* 18, 24–30. doi: 10.1016/j.coi.2005.11.004

Brown, R. L., Kazan, K., McGrath, K. C., Maclean, D. J., and Manners, J. M. (2003). A role for the GCC-box in jasmonate-mediated activation of the *PDF1.2* gene of Arabidopsis. *Plant Physiol.* 132, 1020–1032. doi: 10.1104/pp.102.017814

Browse, J. (2009). Jasmonate passes muster: a receptor and targets for the defense hormone. *Annu. Rev. Plant Biol.* 60, 183–205. doi: 10.1146/annurev.arplant.043008.092007

Bustin, S. A., Benes, V., Garson, J. A., Hellemans, J., Huggett, J., Kubista, M., et al. (2009). The MIQE guidelines: minimum information for publication of quantitative real-time PCR experiments. *Clin. Chem.* 55, 611–622. doi: 10.1373/clinchem.2008.112797

Carvalhais, L. C., Dennis, P. G., Badri, D. V., Tyson, G. W., Vivanco, J. M., and Schenk, P. M. (2013). Activation of the jasmonic acid plant defence pathway alters the composition of rhizosphere bacterial communities. *PLoS ONE* 8:e56457. doi: 10.1371/journal.pone.0056457

Carvalho, A. D. O., and Gomes, V. M. (2011). Plant defensins and defensin-like peptides - biological activities and biotechnological applications. *Curr. Pharm. Des.* 17, 4270–4293. doi: 10.2174/138161211798999437

Cheong, J. J., and Choi, Y. D. (2003). Methyl jasmonate as a vital substance in plants. *Trends Genet.* 19, 409–413. doi: 10.1016/S0168-9525(03)00138-0

Chico, J. M., Chini, A., Fonseca, S., and Solano, R. (2008). JAZ repressors set the rhythm in jasmonate signaling. *Curr. Opin. Plant Biol.* 11, 486–494. doi: 10.1016/j.pbi.2008.06.003

Chini, A., Boter, M., and Solano, R. (2009). Plant oxylipins: COI1/JAZs/MYC2 as the core jasmonic acid-signalling module. *FEBS J.* 276, 4682–4692. doi: 10.1111/j.1742-4658.2009.07194.x

Clauss, M. J., and Koch, M. A. (2006). Poorly known relatives of *Arabidopsis thaliana*. *Trends Plant Sci.* 11, 449–459. doi: 10.1016/j.tplants.2006.07.005

De Coninck, B., Cammue, B. P. A., and Thevissen, K. (2013). Modes of antifungal action and *in planta* functions of plant defensins and defensin-like peptides. *Fungal Biol. Rev.* 26, 109–120. doi: 10.1016/j.fbr.2012.10.002

De Coninck, B. M., Sels, J., Venmans, E., Thys, W., Goderis, I. J., Carron, D., et al. (2010). *Arabidopsis thaliana* plant defensin *AtPDF1.1* is involved in the plant response to biotic stress. *New Phytol.* 187, 1075–1088. doi: 10.1111/j.1469-8137.2010.03326.x

Deinlein, U., Weber, M., Schmidt, H., Rensch, S., Trampczynska, A., Hansen, T. H., et al. (2012). Elevated nicotianamine levels in *Arabidopsis halleri* roots play a key role in zinc hyperaccumulation. *Plant Cell* 24, 708–723. doi: 10.1105/tpc.111.095000

Fones, H., Davis, C. A., Rico, A., Fang, F., Smith, J. A., and Preston, G. M. (2010). Metal hyperaccumulation armors plants against disease. *PLoS Pathog.* 6:e1001093. doi: 10.1371/journal.ppat.1001093

Fones, H. N., Eyles, C. J., Bennett, M. H., Smith, J. A., and Preston, G. M. (2013). Uncoupling of reactive oxygen species accumulation and defence signalling in the metal hyperaccumulator plant *Noccaea caerulescens*. *New Phytol.* doi: 10.1111/nph.12354

Fonseca, S., Chico, J. M., and Solano, R. (2009). The jasmonate pathway: the ligand, the receptor and the core signalling module. *Curr. Opin. Plant Biol.* 12, 539–547. doi: 10.1016/j.pbi.2009.07.013

Freeman, J. L., Garcia, D., Kim, D., Hopf, A., and Salt, D. E. (2005). Constitutively elevated salicylic acid signals glutathione-mediated nickel tolerance in Thlaspi nickel hyperaccumulators. *Plant Physiol.* 137, 1082–1091. doi: 10.1104/pp.104.055293

Fujita, M., Fujita, Y., Noutoshi, Y., Takahashi, F., Narusaka, Y., Yamaguchi-Shinozaki, K., et al. (2006). Crosstalk between abiotic and biotic stress responses: a current view from the points of convergence in the stress signaling networks. *Curr. Opin. Plant Biol.* 9, 436–442. doi: 10.1016/j.pbi.2006.05.014

Gachomo, E. W., Jimenez-Lopez, J. C., Kayode, A. P., Baba-Moussa, L., and Kotchoni, S. O. (2012). Structural characterization of plant defensin protein superfamily. *Mol. Biol. Rep.* 39, 4461–4469. doi: 10.1007/s11033-011-1235-y

Ganz, T. (2003). Defensins: antimicrobial peptides of innate immunity. *Nat. Rev. Immunol.* 3, 710–720. doi: 10.1038/nri1180

Germain, H., Lachance, D., Pelletier, G., Fossdal, C. G., Solheim, H., and Seguin, A. (2012). The expression pattern of the *Picea glauca Defensin 1* promoter is maintained in *Arabidopsis thaliana*, indicating the conservation of signalling pathways between angiosperms and gymnosperms. *J. Exp. Bot.* 63, 785–795. doi: 10.1093/jxb/err303

Gfeller, A., Liechti, R., and Farmer, E. E. (2010). *Arabidopsis* jasmonate signaling pathway. *Sci. Signal.* 3:cm4. doi: 10.1126/scisignal.3109cm4

Glazebrook, J. (2005). Contrasting mechanisms of defense against biotrophic and necrotrophic pathogens. *Annu. Rev. Phytopathol.* 43, 205–227. doi: 10.1146/annurev.phyto.43.040204.135923

Glombitza, S., Dubuis, P. H., Thulke, O., Welzl, G., Bovet, L., Gotz, M., et al. (2004). Crosstalk and differential response to abiotic and biotic stressors reflected at the transcriptional level of effector genes from secondary metabolism. *Plant Mol. Biol.* 54, 817–835. doi: 10.1007/s11103-004-0274-3

Hammond, J. P., Bowen, H. C., White, P. J., Mills, V., Pyke, K. A., Baker, A. J., et al. (2006). A comparison of the *Thlaspi caerulescens* and *Thlaspi arvense* shoot transcriptomes. *New Phytol.* 170, 239–260. doi: 10.1111/j.1469-8137.2006.01662.x

Hanikenne, M., Talke, I. N., Haydon, M. J., Lanz, C., Nolte, A., Motte, P., et al. (2008). Evolution of metal hyperaccumulation required *cis*-regulatory changes and triplication of *HMA4*. *Nature* 453, 391–395. doi: 10.1038/nature06877

Hiruma, K., Nishiuchi, T., Kato, T., Bednarek, P., Okuno, T., Schulze-Lefert, P., et al. (2011). Arabidopsis *ENHANCED DISEASE RESISTANCE 1* is required for pathogen-induced expression of plant defensins in nonhost resistance, and acts through interference of *MYC2*-mediated repressor function. *Plant J.* 67, 980–992. doi: 10.1111/j.1365-313X.2011.04651.x

Hörger, A., Fones, H., and Preston, G. M. (2013). The current status of the elemental defense hypothesis in relation to pathogens. *Front. Plant Physiol.* 4:395. doi: 10.3389/fpls.2013.00395

Hossain, M. A., Munemasa, S., Uraji, M., Nakamura, Y., Mori, I. C., and Murata, Y. (2011). Involvement of endogenous abscisic acid in methyl jasmonate-induced stomatal closure in Arabidopsis. *Plant Physiol.* 156, 430–438. doi: 10.1104/pp.111.172254

Hurles, M. (2004). Gene duplication: the genomic trade in spare parts. *PLoS Biol.* 2:e206. doi: 10.1371/journal.pbio.0020206

Jacob, F. (1977). Evolution and tinkering. *Science* 196, 1161–1166. doi: 10.1126/science.860134

Kazan, K., and Manners, J. M. (2011). The interplay between light and jasmonate signalling during defence and development. *J. Exp. Bot.* 62, 4087–4100. doi: 10.1093/jxb/err142

Koch, M. A., and Matschinger, M. (2007). Evolution and genetic differentiation among relatives of Arabidopsis thaliana. *Proc. Natl. Acad. Sci. U.S.A.* 104, 6272–6277. doi: 10.1073/pnas.0701338104

Kombrink, E. (2012). Chemical and genetic exploration of jasmonate biosynthesis and signaling paths. *Planta* 236, 1351–1366. doi: 10.1007/s00425-012-1705-z

Koonin, E. V. (2005). Orthologs, paralogs, and evolutionary genomics. *Annu. Rev. Genet.* 39, 309–338. doi: 10.1146/annurev.genet.39.073003.114725

Kramer, U. (2010). Metal hyperaccumulation in plants. *Annu. Rev. Plant Biol.* 61, 517–534. doi: 10.1146/annurev-arplant-042809-112156

Lay, F. T., and Anderson, M. A. (2005). Defensins-components of the innate immune system in plants. *Curr. Protein Pept. Sci.* 6, 85–101. doi: 10.2174/1389203053027575

Lescot, M., Dehais, P., Thijs, G., Marchal, K., Moreau, Y., Van De Peer, Y., et al. (2002). PlantCARE, a database of plant cis-acting regulatory elements and a portal to tools for *in silico* analysis of promoter sequences. *Nucleic Acids Res.* 30, 325–327. doi: 10.1093/nar/30.1.325

Macnair, M. R. (2003). The hyperaccumulation of metals by plants. *Adv. Bot. Res.* 40, 63–105. doi: 10.1016/S0065-2296(05)40002-6

Maestri, E., Marmiroli, M., Visioli, G., and Marmiroli, N. (2010). Metal tolerance and hyperaccumulation: costs and trade-offs between traits and environment. *Environ. Exp. Bot.* 68, 1–13. doi: 10.1016/j.envexpbot.2009.10.011

Manners, J. M., Penninckx, I. A., Vermaere, K., Kazan, K., Brown, R. L., Morgan, A., et al. (1998). The promoter of the plant defensin gene *PDF1.2* from Arabidopsis is systemically activated by fungal pathogens and responds to methyl jasmonate but not to salicylic acid. *Plant Mol. Biol.* 38, 1071–1080. doi: 10.1023/A:1006070413843

Marques, L., and Oomen, R. J. (2011). On the way to unravel zinc hyperaccumulation in plants: a mini review. *Metallomics* 3, 1265–1270. doi: 10.1039/c1mt00117e

Marques, L., Oomen, R. J., Aumelas, A., Le Jean, M., and Berthomieu, P. (2009). Production of an *Arabidopsis halleri* foliar defensin in *Escherichia coli*. *J. Appl. Microbiol.* 106, 1640–1648. doi: 10.1111/j.1365-2672.2008.04131.x

Memelink, J. (2009). Regulation of gene expression by jasmonate hormones. *Phytochemistry* 70, 1560–1570. doi: 10.1016/j.phytochem.2009.09.004

Mirouze, M., Sels, J., Richard, O., Czernic, P., Loubet, S., Jacquier, A., et al. (2006). A putative novel role for plant defensins: a defensin from the zinc hyper-accumulating plant, *Arabidopsis halleri*, confers zinc tolerance. *Plant J.* 47, 329–342. doi: 10.1111/j.1365-313X.2006.02788.x

Mithofer, A., Schulze, B., and Boland, W. (2004). Biotic and heavy metal stress response in plants: evidence for common signals. *FEBS Lett.* 566, 1–5. doi: 10.1016/j.febslet.2004.04.011

Murashige, T., and Skoog, F. (1962). A revised medium for rapid growth and bioassays with tobacco tissue cultures. *Physiol. Plant* 15, 473–497. doi: 10.1111/j.1399-3054.1962.tb08052.x

Niu, Y., Figueroa, P., and Browse, J. (2011). Characterization of JAZ-interacting bHLH transcription factors that regulate jasmonate responses in Arabidopsis. *J. Exp. Bot.* 62, 2143–2154. doi: 10.1093/jxb/erq408

Oliver, R. P., and Ipcho, S. V. (2004). *Arabidopsis* pathology breathes new life into the necrotrophs-vs.-biotrophs classification of fungal pathogens. *Mol. Plant Pathol.* 5, 347–352. doi: 10.1111/j.1364-3703.2004.00228.x

Penninckx, I. A., Eggermont, K., Terras, F. R., Thomma, B. P., De Samblanx, G. W., Buchala, A., et al. (1996). Pathogen-induced systemic activation of a plant

defensin gene in *Arabidopsis* follows a salicylic acid-independent pathway. *Plant Cell* 8, 2309–2323.

Penninckx, I. A., Thomma, B. P., Buchala, A., Metraux, J. P., and Broekaert, W. F. (1998). Concomitant activation of jasmonate and ethylene response pathways is required for induction of a plant defensin gene in *Arabidopsis*. *Plant Cell* 10, 2103–2113. doi: 10.1105/tpc.10.12.2103

Pfaffl, M. W. (2001). A new mathematical model for relative quantification in real-time RT-PCR. *Nucleic Acids Res.* 29:e45. doi: 10.1093/nar/29.9.e45

Pfaffl, M. W., Horgan, G. W., and Dempfle, L. (2002). Relative expression software tool (REST) for group-wise comparison and statistical analysis of relative expression results in real-time PCR. *Nucleic Acids Res.* 30:e36. doi: 10.1093/nar/30.9.e36

Pieterse, C. M., Van Der Does, D., Zamioudis, C., Leon-Reyes, A., and Van Wees, S. C. (2012). Hormonal modulation of plant immunity. *Annu. Rev. Cell Dev. Biol.* 28, 489–521. doi: 10.1146/annurev-cellbio-092910-154055

Poschenrieder, C., Tolra, R., and Barcelo, J. (2006). Can metals defend plants against biotic stress? *Trends Plant Sci.* 11, 288–295. doi: 10.1016/j.tplants.2006.04.007

Rascio, N., and Navari-Izzo, F. (2011). Heavy metal hyperaccumulating plants: how and why do they do it? And what makes them so interesting? *Plant Sci.* 180, 169–181. doi: 10.1016/j.plantsci.2010.08.016

RCoreTeam. (2012). *R: A Language and Environment for Statistical Computing.* Vienna: R Foundation for Statistical Computing.

Roosens, N. H., Willems, G., and Saumitou-Laprade, P. (2008). Using Arabidopsis to explore zinc tolerance and hyperaccumulation. *Trends Plant Sci.* 13, 208–215. doi: 10.1016/j.tplants.2008.02.006

Roux, C., Castric, V., Pauwels, M., Wright, S. I., Saumitou-Laprade, P., and Vekemans, X. (2011). Does speciation between *Arabidopsis halleri* and *Arabidopsis lyrata* coincide with major changes in a molecular target of adaptation? *PLoS ONE* 6:e26872. doi: 10.1371/journal.pone.0026872

Sagaram, U. S., Kaur, J., and Shah, D. M. (2012). "Antifungal plant defensins: structure -activity relationships, mode of action, and biotech applications," in *Small Wonders: Peptides for disease control,* ed K. Rajasekaran (Washinton, DC: American Chemical Society), 317–336.

Santino, A., Taurino, M., De Domenico, S., Bonsegna, S., Poltronieri, P., Pastor, V., et al. (2013). Jasmonate signaling in plant development and defense response to multiple (a)biotic stresses. *Plant Cell Rep.* 32, 1085–1098. doi: 10.1007/s00299-013-1441-2

Schranz, M. E., Song, B. H., Windsor, A. J., and Mitchell-Olds, T. (2007). Comparative genomics in the *Brassicaceae*: a family-wide perspective. *Curr. Opin. Plant Biol.* 10, 168–175. doi: 10.1016/j.pbi.2007.01.014

Shahzad, Z., Gosti, F., Frerot, H., Lacombe, E., Roosens, N., Saumitou-Laprade, P., et al. (2010). The five *AhMTP1* zinc transporters undergo different evolutionary fates towards adaptive evolution to zinc tolerance in *Arabidopsis halleri*. *PLoS Genet.* 6:e1000911. doi: 10.1371/journal.pgen.1000911

Shahzad, Z., Ranwez, V., Fizames, C., Marques, L., Le Martret, B., Alassimone, J., et al. (2013). *Plant Defensin type 1* (*PDF1*): protein promiscuity and expression variation within the *Arabidopsis* genus shed light on zinc tolerance acquisition in *Arabidopsis halleri*. *New Phytol.* 200, 820–833. doi: 10.1111/nph.12396

Shan, X. Y., Wang, Z. L., and Xie, D. (2007). Jasmonate signal pathway in *Arabidopsis*. *J. Integr. Plant Biol.* 49, 81–86. doi: 10.1111/j.1744-7909.2006.00416.x

Silverstein, K. A., Graham, M. A., Paape, T. D., and Vandenbosch, K. A. (2005). Genome organization of more than 300 defensin-like genes in *Arabidopsis*. *Plant Physiol.* 138, 600–610. doi: 10.1104/pp.105.060079

Silverstein, K. A., Moskal, W. A. Jr., Wu, H. C., Underwood, B. A., Graham, M. A., Town, C. D., et al. (2007). Small cysteine-rich peptides resembling antimicrobial peptides have been under-predicted in plants. *Plant J.* 51, 262–280. doi: 10.1111/j.1365-313X.2007.03136.x

Sims, G. E., Jun, S. R., Wu, G. A., and Kim, S. H. (2009). Alignment-free genome comparison with feature frequency profiles (FFP) and optimal resolutions. *Proc. Natl. Acad. Sci. U.S.A.* 106, 2677–2682. doi: 10.1073/pnas.0813249106

Soulie, M. C., Perino, C., Piffeteau, A., Choquer, M., Malfatti, P., Cimerman, A., et al. (2006). *Botrytis cinerea* virulence is drastically reduced after disruption of chitin synthase class III gene (*Bcchs3a*). *Cell. Microbiol.* 8, 1310–1321. doi: 10.1111/j.1462-5822.2006.00711.x

Svyatyna, K., and Riemann, M. (2012). Light-dependent regulation of the jasmonate pathway. *Protoplasma* 249(Suppl. 2), S137–S145. doi: 10.1007/s00709-012-0409-3

Talke, I. N., Hanikenne, M., and Kramer, U. (2006). Zinc-dependent global transcriptional control, transcriptional deregulation, and higher gene copy number for genes in metal homeostasis of the hyperaccumulator *Arabidopsis halleri*. *Plant Physiol.* 142, 148–167. doi: 10.1104/pp.105.076232

Thomma, B. P., Cammue, B. P., and Thevissen, K. (2002). Plant defensins. *Planta* 216, 193–202. doi: 10.1007/s00425-002-0902-6

Thomma, B. P., Eggermont, K., Penninckx, I. A., Mauch-Mani, B., Vogelsang, R., Cammue, B. P., et al. (1998). Separate jasmonate-dependent and salicylate-dependent defense-response pathways in *Arabidopsis* are essential for resistance to distinct microbial pathogens. *Proc. Natl. Acad. Sci. U.S.A.* 95, 15107–15111. doi: 10.1073/pnas.95.25.15107

Turner, J. G., Ellis, C., and Devoto, A. (2002). The jasmonate signal pathway. *Plant Cell* 14(Suppl.), S153–S164. doi: 10.1105/tpc.000679

Van De Mortel, J. E., Almar Villanueva, L., Schat, H., Kwekkeboom, J., Coughlan, S., Moerland, P. D., et al. (2006). Large expression differences in genes for iron and zinc homeostasis, stress response, and lignin biosynthesis distinguish roots of *Arabidopsis thaliana* and the related metal hyperaccumulator *Thlaspi caerulescens*. *Plant Physiol.* 142, 1127–1147. doi: 10.1104/pp.106.082073

Van Der Ent, A., Baker, A. J., Reeves, R. D., Pollard, A. J., and Schat, H. (2012). Q8 Hyperaccumulators of metal and metalloid trace elements: facts and fiction. *Plant Soil* 362, 319–334.

Van Der Weerden, N. L., Bleackley, M. R., and Anderson, M. A. (2013). Properties Q9 and mechanisms of action of naturally occurring antifungal peptides. *Cell Mol. Life Sci.* doi: 10.1007/s00018-013-1260-1

Verbruggen, N., Hermans, C., and Schat, H. (2009). Molecular mechanisms of metal hyperaccumulation in plants. *New Phytol.* 181, 759–776. doi: 10.1111/j.1469-8137.2008.02748.x

Verhage, A., Vlaardingerbroek, I., Raaymakers, C., Van Dam, N. M., Dicke, M., Van Wees, S. C., et al. (2011). Rewiring of the jasmonate signaling pathway in arabidopsis during insect herbivory. *Front. Plant Sci.* 2:47. doi: 10.3389/fpls.2011.00047

Wang, Y., Liu, G. J., Yan, X. F., Wei, Z. G., and Xu, Z. R. (2011). MeJA-inducible Q8 expression of the heterologous *JAZ2* promoter from Arabidopsis in *Populus trichocarpa* protoplasts. *J. Plant Dis. Prot.* 118, 69–74.

Wasternack, C., and Hause, B. (2013). Jasmonates: biosynthesis, perception, signal transduction and action in plant stress response, growth and development. An update to the 2007 review in Annals of Botany. *Ann. Bot.* 111, 1021–1058. doi: 10.1093/aob/mct067

Wong, J. H., Xia, L., and Ng, T. B. (2007). A review of defensins of diverse origins. *Curr. Protein Pept. Sci.* 8, 446–459. doi: 10.2174/138920307782411446

Yan, J., Zhang, C., Gu, M., Bai, Z., Zhang, W., Qi, T., et al. (2009). The Arabidopsis CORONATINE INSENSITIVE1 protein is a jasmonate receptor. *Plant Cell* 21, 2220–2236. doi: 10.1105/tpc.109.065730

Zarei, A., Korbes, A. P., Younessi, P., Montiel, G., Champion, A., and Memelink, J. (2011). Two GCC boxes and AP2/ERF-domain transcription factor ORA59 in jasmonate/ethylene-mediated activation of the PDF1.2 promoter in Arabidopsis. *Plant Mol. Biol.* 75, 321–331. doi: 10.1007/s11103-010-9728-y

Zimmerli, L., Stein, M., Lipka, V., Schulze-Lefert, P., and Somerville, S. (2004). Host and non-host pathogens elicit different jasmonate/ethylene responses in *Arabidopsis*. *Plant J.* 40, 633–646. doi: 10.1111/j.1365-313X.2004.02236.x

Conflict of Interest Statement: The authors declare that the research was conducted in the absence of any commercial or financial relationships that could be construed as a potential conflict of interest.

Received: 15 July 2013; accepted: 10 February 2014; published online: xx February 2014.

Citation: Nguyen NNT, Ranwez V, Vile D, Soulie M-C, Dellagi A, Expert D and Gosti F (2014) Evolutionary tinkering of the expression of PDF1s suggests their joint effect on zinc tolerance and the response to pathogen attack. Front. Plant Sci. ***5****:70. doi: 10.3389/fpls.2014.00070*

This article was submitted to Plant Physiology, a section of the journal Frontiers in Plant Science.

Copyright © 2014 Nguyen, Ranwez, Vile, Soulie, Dellagi, Expert and Gosti. This is an open-access article distributed under the terms of the Creative Commons Attribution License (CC BY). The use, distribution or reproduction in other forums is permitted, provided the original author(s) or licensor are credited and that the original publication in this journal is cited, in accordance with accepted academic practice. No use, distribution or reproduction is permitted which does not comply with these terms.
