# Supplementary material for: Evolutionary tinkering of the expression of PDF1s suggests their joint effect on zinc tolerance and the response to pathogen attack
Source: Front Plant Sci. 2014 Mar 11;5:70. doi: 10.3389/fpls.2014.00070 (PMC3949115; doi:10.3389/fpls.2014.00070)
Supplement: Supplementary Table 1 — Gene specific primer pairs used in quantitative RT-PCR analyses. [file DataSheet1.PDF]

Listing of supplementary tables

Supplementary Table 1: Gene specific primer pairs used in qRT-PCR analyses
Supplementary Table 2: Values of threshold cycles (C_t) obtained in qRT-PCR analyses performed in transferred assay and in germination assay (Excell Table)
Supplementary Table 3: Values of Kruskal Wallis tests comparing the *PDF1* Relative Expression Levels to Actin determined for *A. thaliana* and *A. halleri* plants upon transfer to MeJA or $ZnSO_4$.
Supplementary Table 4: Median values and one-standard error equivalent and 95% confidence intervals calculated on *PDF1* REL ratios after pairwise bootstrap of C_t and primer efficiencies values.
Supplementary Table 5: Occurrence of MeJA responsive motifs in the 500 bp upstream region of PDF1s in *A. halleri* and *A. thaliana*
Supplementary Table 6: Shoot dry weight measured for pools of *A. thaliana* seedlings collected 9 days following germination in control condition or in presence of MeJA 5 µM or $ZnSO_4$ 100 µM or both MeJA 5 µM and $ZnSO_4$ 100 µM.

Listing of supplementary file
Supplementary File 1: Nucleic sequences of the putative PDF1 promoter region used for in silico motif searches.

SUPPLEMENTARY TABLES

Supplementary Table 1: Gene specific primer pairs used in qRT-PCR analyses

Gene name	Primer name	Primer sequences $5^{/} \ldots 3^{/}$	Ann. temp. (°C)	PCR efficiencies (E)
AtPDF1.1	AtPDF1.1F	GCGGAAACAGTAACGCGTGCA	64	1.89
	AtPDF1.1R	TTAAGCATTAAGAGCTCTTGGTAGA		
AtPDF1.2a	AtPDF1.2aF	GCTAAATCGTGTGTATTTTACATA	61	1.88
	AtPDF1.2R	GTAACAACAACGGGAAAATAAACA		
AtPDF1.2b	AtPDF1.2bF	GGTTCTTTATCGATGTGTATTTATAT	61	1.70
	AtPDF1.2R	TTTATAGAAAAGTCACACCAAACATT		
AtPDF1.2c	AtPDF1.2cF	CTTGATCGTGTGTGTATTTTACTTT	61	1.80
	AtPDF1.2R	GTATTATTATAACAAGGAGAAACCA		
AtPDF1.3	AtPDF1.3F	GCTACCAATGACTACCAATTATACT	61	1.76
	AtPDF1.3R	CAACAACAAAGCAAACGAAACA		
AtPDF1.4	AtPDF1.4F	GAATCTTCTTCAACGGTCACTTTTA	61	1.93
	AtPDF1.4R	GAAACCCAAGCTAAAGATAACGGT		
AtPDF1.5	AtPDF1.5F	GTACCACCATTACCCTTATTTTGGT	64	1.75
	AtPDF1.5R	CCCTTTTGCAAAGCTCTGCTTTCA		
AtActin	ActinF	GGTAACATTGTGCTCAGTGGTGG	64	1.76
	ActinR	AACGACCTTAATCTTCATGCTGC		

Supplementary Table 2: Values of threshold cycles (C_t) obtained in qRT-PCR analyses performed in transferred assay and in germination assay (provided as an Excell file)

Supplementary Table 3: Mean values of *PDF1* Relative Expression Levels to Actin determined for *A. thaliana* and *A. halleri* plants upon transfer to MeJA or $ZnSO_4$[1].

PDF1 transcripts	Material type	Control	MeJA 5 µM	MeJA 50 µM	$ZnSO_4$ 100 µM
AhPDF1.1a	Shoots	0.8518^{a}	0.8069^{a}	1.152^{a}	1.2624^{a}
	Roots	0.1415^{b}	0.2468^{ab}	0.2091^{ab}	0.6646^{a}
AhPDF1.1b	Shoots	145.2571^{a}	76.9397^{a}	81.2279^{a}	69.2576^{a}
	Roots	0.0051^{a}	0.0062^{a}	0.0137^{a}	0.0058^{a}
AhPDF1.2b	Shoots	0.0010^{c}	0.0356^{a}		0.0030^{b}
	Roots				
AhPDF1.4	Shoots	0.0242^{a}	0.0075^{b}	0.1036^{ab}	0.0173^{ab}
	Roots	0.0003^{ab}	0.0002^{ab}	0.0001^{b}	0.0003^{a}
AhPDF1.5	Shoots	0.0214^{a}	0.0427^{a}	0.0197^{a}	0.0144^{a}
	Roots	0.0041^{a}	0.0042^{a}	0.0034^{a}	
AhPDF1.8a	Shoots	0.0574^{b}	0.0068^{c}		0.0148^{c}
	Roots	0.0655^{b}	0.5322^{a}	0.0008^{d}	0.0341^{c}
AtPDF1.1	Shoots	0.0086^{b}	0.0096^{ab}	0.0090^{b}	0.0157^{a}
	Roots	0.0041^{a}	0.0026^{a}	0.0022^{a}	0.0036^{a}
AtPDF1.2a	Shoots	2.0856^{c}	4.3536^{b}	27.5038^{a}	4.0985^{b}
	Roots	0.0019^{b}	0.0013^{b}	0.0130^{a}	0.0026^{b}
AtPDF1.2b	Shoots	0.0374^{c}	0.06220^{b}	0.2820^{a}	0.0440^{bc}
	Roots				
AtPDF1.2c	Shoots	1.1064^{c}	4.3617^{b}	26.9409^{a}	1.0406^{c}
	Roots				
AtPDF1.3	Shoots	17.3394^{a}	15.7624^{a}	24.4057^{a}	25.6240^{a}
	Roots	0.06868^{a}	0.0216^{b}	0.11072^{a}	0.07285^{a}
AtPDF1.4	Shoots	0.5602^{bc}	0.3797^{c}	1.2713^{a}	0.7165^{b}
	Roots	0.0406^{a}	0.0193^{b}	0.0109^{b}	0.0459^{a}
AtPDF1.5	Shoots	0.1319^{bc}	0.0836^{c}	0.2192^{a}	0.1572^{ab}
	Roots	0.0164^{ab}	0.0083^{b}	0.0243^{a}	0.0220^{a}

[1] Different letters appearing in exponential represent significant differences after Kruskal Wallis and are ranked by decreasing expression levels. Empty cases indicate that *PDF1* transcripts quantification was not detected.

Supplementary Table 5: Mean and median ratios of relative expression levels (R) of *PDF1* genes calculated from mean C_t values, and after pairwise bootstrap of C_t and primer efficiencies values, respectively. Lower and upper one-standard error equivalent (SE) and 95% confidence intervals (CI) from bootstrapped R values are presented.

PDF1 transcripts	Material type	Condition	$R_{mean\ Ct}$	R_{median}	SE_{lower}	SE_{upper}	CI_{lower}	CI_{upper}
		Arabidopsis halleri						
AhPDF1.1a	Shoot	MeJA 5 µM	0.606	0.931	0.107	2.563	0.025	5.685
		MeJA 50 µM	1.521	1.699	0.676	2.887	0.422	6.447
		$ZnSO_4$ 100 µM	1.333	1.377	0.439	3.791	0.148	7.152
	Root	MeJA 5 µM	2.037	2.521	0.323	6.954	0.066	104.610
		MeJA 50 µM	2.117	2.705	0.228	11.005	0.018	88.907
		$ZnSO_4$ 100 µM	9.334	7.566	1.361	56.094	0.198	110.513
AhPDF1.1b	Shoot	MeJA 5 µM	0.516	0.466	0.226	1.124	0.135	2.327
		MeJA 50 µM	0.522	0.498	0.219	1.303	0.110	3.775
		$ZnSO_4$ 100 µM	0.552	0.527	0.232	1.307	0.133	3.421
	Root	MeJA 5 µM	0.824	1.010	0.227	2.799	0.099	5.324
		MeJA 50 µM	1.351	1.731	0.196	4.319	0.060	11.446
		$ZnSO_4$ 100 µM	1.045	1.078	0.510	2.160	0.188	4.933
AhPDF1.2b	Shoot	MeJA 5 µM	25.843	21.411	8.460	74.104	6.529	161.667
		$ZnSO_4$ 100 µM	2.908	3.129	1.517	4.581	1.282	9.450
AhPDF1.4	Shoot	MeJA 5 µM	0.290	0.249	0.128	0.711	0.094	1.345
		MeJA 50 µM	0.395	0.296	0.191	1.009	0.131	1.423
		$ZnSO_4$ 100 µM	0.655	0.745	0.262	1.494	0.132	5.102
	Root	MeJA 5 µM	0.711	0.793	0.253	2.620	0.047	7.456
		MeJA 50 µM	0.347	0.363	0.130	0.792	0.030	2.090
		$ZnSO_4$ 100 µM	1.188	1.681	0.293	3.373	0.175	9.402
AhPDF1.5	Shoot	MeJA 5 µM	1.593	1.886	0.446	5.195	0.145	11.037
		MeJA 50 µM	0.911	0.875	0.384	1.993	0.166	4.334
	Root	MeJA 5 µM	0.885	0.900	0.461	2.529	0.164	3.215
		MeJA 50 µM	0.658	0.864	0.247	2.074	0.088	3.088
AhPDF1.8a	Shoot	MeJA 5 µM	0.156	0.129	0.062	0.483	0.039	1.185
		$ZnSO_4$ 100 µM	0.303	0.281	0.107	0.862	0.035	3.494
	Root	MeJA 5 µM	4.851	6.073	1.180	16.467	0.573	43.396
		MeJA 50 µM	0.009	0.011	0.003	0.022	0.001	0.054
		$ZnSO_4$ 100 µM	0.528	0.556	0.293	0.849	0.122	1.760
		Arabidopsis thaliana						
AtPDF1.1	Shoot	MeJA 5 µM	1.1827	1.2781	0.7295	2.2439	0.2947	3.9315
		MeJA 50 µM	1.0779	1.0782	0.5624	1.9359	0.2272	3.2374
		$ZnSO_4$ 100 µM	1.9768	2.1193	1.0520	3.6397	0.7087	4.5626
	Root	MeJA 5 µM	0.5546	0.4522	0.3843	0.9740	0.2171	1.7993
		MeJA 50 µM	0.3111	0.2873	0.0468	1.1932	0.0229	1.8224
		$ZnSO_4$ 100 µM	0.6844	0.6948	0.2043	1.6517	0.1177	3.3754
AtPDF1.2a	Shoot	MeJA 5 µM	2.5041	2.6051	1.2370	5.6339	0.4451	12.5537
		MeJA 50 µM	16.2419	15.3953	8.9589	35.5828	3.6658	77.6820
		$ZnSO_4$ 100 µM	2.1966	1.9413	1.2158	5.2531	0.4375	15.2523
	Root	MeJA 5 µM	0.6821	0.6998	0.3188	1.1594	0.2138	3.3256
		MeJA 50 µM	5.8140	6.6620	2.8438	11.1652	0.7135	30.5632

		$ZnSO_4$ 100 μM	1.2550	1.0877	0.5915	2.9775	0.3570	9.0448
AtPDF1.2b	Shoot	MeJA 5 μM	1.5455	1.4998	1.0105	2.5709	0.7027	3.6702
		MeJA 50 μM	6.7697	6.1870	3.7348	12.1517	2.4645	15.6641
		$ZnSO_4$ 100 μM	1.1407	1.1421	0.7964	1.6963	0.5187	2.0855
AtPDF1.2c	Shoot	MeJA 5 μM	4.0125	4.2917	2.4238	6.9901	1.2774	11.3602
		MeJA 50 μM	25.5906	25.1742	13.2331	46.8718	8.8085	76.1755
		$ZnSO_4$ 100 μM	0.9058	0.8939	0.4448	1.6350	0.2310	3.7372
AtPDF1.3	Shoot	MeJA 5 μM	0.9597	1.0133	0.5544	1.6790	0.3656	2.6893
		MeJA 50 μM	1.1972	1.0582	0.5319	2.6391	0.2862	8.8985
		$ZnSO_4$ 100 μM	1.4518	1.3217	0.7110	3.1387	0.3468	6.9389
	Root	MeJA 5 μM	0.3099	0.2944	0.1756	0.5370	0.1040	0.8159
		MeJA 50 μM	1.1945	1.4605	0.2983	3.8544	0.1367	4.5069
		$ZnSO_4$ 100 μM	1.0242	0.8965	0.6127	1.6695	0.3789	3.7118
AtPDF1.4	Shoot	MeJA 5 μM	0.7538	0.8102	0.4411	1.4985	0.1641	3.3615
		MeJA 50 μM	2.5676	2.6264	1.8182	4.7246	0.6765	6.8189
		$ZnSO_4$ 100 μM	1.4666	1.6489	0.6624	3.0854	0.3018	4.8395
	Root	MeJA 5 μM	0.4270	0.3325	0.2496	0.8606	0.1117	1.2161
		MeJA 50 μM	0.2546	0.3028	0.1367	0.3856	0.0581	0.7125
		$ZnSO_4$ 100 μM	0.9278	0.8639	0.3476	2.4556	0.1538	2.9947
AtPDF1.5	Shoot	MeJA 5 μM	0.6120	0.5934	0.3675	1.0272	0.2229	1.5144
		MeJA 50 μM	1.7065	1.7386	1.1000	2.6857	0.6672	3.3625
		$ZnSO_4$ 100 μM	1.2067	1.0994	0.8107	1.8429	0.4471	2.2543
	Root	MeJA 5 μM	0.5390	0.5684	0.2022	1.4286	0.1263	2.0604
		MeJA 50 μM	1.5464	1.6933	0.5691	3.5831	0.3704	7.4954
		$ZnSO_4$ 100 μM	1.2761	1.3921	0.4570	3.6459	0.1714	7.6268

Supplementary Table 5: Occurrence of MeJA responsive motifs present in 500 bp upstream region of *PDF1*s in *A. halleri* and *A. thaliana*.

Locus containing *PDF1*s[1]	Gene name[2]	Name and sequence of motifs			
		As-1-type[3] CGTCA	G-box[4] CACGTG	G-box like[5] AACGTG	GCC-box[6] GCCGCC
locus 1	*AhPDF1.1a*				
	AtPDF1.1				
	AhPDF1.1b				
locus 2	*AhPDF1.2a*				1
	AtPDF1.2a	2		2	1
	AhPDF1.2c				
	AtPDF1.2c				
locus 3	*AhPDF1.2b*			2	1
	AtPDF1.2b				1
	AtPDF1.3				
locus 4	*AhPDF1.4*	2			
	AtPDF1.4	2			
locus 5	*AhPDF1.5*		2		
	AtPDF1.5				1
locus 7	*AhPDF1.7*	2			
locus 8	*AhPDF1.8a*				
	AhPDF1.8b				

[1] *PDF1*s are grouped by locus according to their orthologous syntenic relationships which are indicated by parenthesis (Shahzad et al., in press).
[2] Genes for which transcripts are responsive to MeJA are indicated with red letters
[3] (Kim et al., 1993)
[4] (Kim et al., 1992)
[5] (Guerineau et al., 2003)
[6] (Brown et al., 2003; Wang et al., 2011)

Supplementary Table 6: Shoot dry weight measured for pools of *A. thaliana* seedlings collected 9 days following germination in control condition or in presence of MeJA 5 µM or $ZnSO_4$ 100 µM or both MeJA 5 µM and $ZnSO_4$ 100 µM.

Condition	Experiment 1		Experiment 2	
	Dry weight (mg)	Number of plants	Dry weight (mg)	Number of plants
Control	8.23	21	6.71	17
Control	8.38	20	6.15	16
Control	7.96	20	6.66	17
Control	8.26	21	6.38	16
Control	8.51	21	6.82	17
Control	8.31	21	6.52	16
Control	8.61	21	6.8	17
Control	8.22	21	5.66	14
MeJA 5 µM	8.37	21	7.25	19
MeJA 5 µM	8.03	20	8.23	20
MeJA 5 µM	8.19	21	7.54	19
MeJA 5 µM	8.14	21	6.8	17
MeJA 5 µM	8.07	19	5.01	13
MeJA 5 µM	8.26	20	6.03	16
MeJA 5 µM	8.28	21	6.2	16
MeJA 5 µM	8.23	21	5.01	12
$ZnSO_4$ 100 µM	5.27	21	3.29	13
$ZnSO_4$ 100 µM	5.2	21	4.15	16
$ZnSO_4$ 100 µM	5.51	20	4.8	18
$ZnSO_4$ 100 µM	5.44	21	4.04	17
$ZnSO_4$ 100 µM	5.38	21	4.22	17
$ZnSO_4$ 100 µM	4.97	20	4.6	18
$ZnSO_4$ 100 µM	5.17	21	4.85	18
$ZnSO_4$ 100 µM	4.99	20	4.62	18
MeJA 5 µM + $ZnSO_4$ 100 µM	6	21	5.74	20
MeJA 5 µM + $ZnSO_4$ 100 µM	6.08	21	5.51	19
MeJA 5 µM + $ZnSO_4$ 100 µM	5.86	20	5.11	17
MeJA 5 µM + $ZnSO_4$ 100 µM	6.31	21	5.62	18
MeJA 5 µM + $ZnSO_4$ 100 µM	5.97	21	5.56	19
MeJA 5 µM + $ZnSO_4$ 100 µM	6	21	6.4	21
MeJA 5 µM + $ZnSO_4$ 100 µM	6.19	21		
MeJA 5 µM + $ZnSO_4$ 100 µM	6.43	21		

Supplementary File 1: Nucleic sequences of the putative PDF1 promoter region used for in silico motif searches.

>AhPDF1_1a
GTCTAGTTTTTATGCTCAGTTGTTTTCAATGTGTATATGTTTTAGCTTGAAGAAATAAGC
TTGAGATCAACCTCTATTTATACTGAAAAAAGGATGGGGGAGAAAGTGTTTAACCCCATA
AACAAATAAAAATCACACCGTCTGATAGTAGAGAATACTAACACGCAAGTATACACCTTT
TGATAGAAGATAATATCTTGGCCAATTCATAAAAAGTGTGCTGATCAATGCTTGTTTCAA
GTGCCACATTTGTTCATTTGCAATTTCATTCGACCGGCTGGTTTGAATTGGTCAACATTT
TAAATATCTTGAGGCTTGATCAAATCCTAGTACATTGGTTATTAAAAAAAAAAAAATCCT
AGTTACACAAGTGGTAGTGATTTGTCGGCCACATCGAAGTCTATAAGTTGGATATTTTCT
ATTGACTTTTCTTCCCTATTCATTTATTGTTACTGAAACCTCCAAGGAGCTACTTCACTA
GCTGGCAAGTTGATAAATTAGGTTATTTCTTCACTTTCTTTGTTATTGATCACATAATCA
TCAAACTAATTAAAATCTACACGTGTAATTACATATTAGTCATATAGTGGATCTGAACAA
ATTTGCAAGAGGGTTTGGACTAATAAATTTGTAATTTCGTAATTCCCACCCCAAACCCAA
CACAAACTTCTTGGATTCAACTCTGTCAAATTCAGACCACCAAAACAAATTACATCCGAT
TTTTATTAACGAGTATTAAAAGTAAATCTAAAAAAGTAAACTAATAATCGAAAGAAAATT
CGTTTTAGACTTCATAGTCGTCATCCGCAAAGTTTGACAAAGACATGTCTCCTCTTTAAA
CATTTTCTGTCGACAGCCACGTGCCAACAACTTGCTTTCACGCCGTACTAAGAGAGCACC
AGCACTATCCATGGACATGTAGCAAACTCAGAAGAACACCACACAAGTTAATTTGTTAGA
TTAGAAAGGCAAGTTATTAATGAGATTTAAAATCCTTAAATTAGATAGAATATTATAGAT
TTAATGTCGTTAGATTATGAATTATATTTGCTTAGATTAAAAACTAAGGAATGGTTAACT
TAGAATCTTTGATTGGCAAAGATGGTAGTTTTGCAAATAATTATCTTTTTTTTAATTACT
TTTTAAATAGAGATGTCAAAATGAGCTAACTCCGATCCGTCGATTCACTTAGTTCACCAT
CGCCGGATTTGTAACCAATATGTGGCTTTCGTTGCTAATATGTGGCAAGTTGGTAAAGGA
TATATATGCCGTGACCACTCTGTCCTTTCATTACATACTGTATAATATTTCTAGCCATAA
GCCATCCGTACGTGGTTTTTCAAGTATTAGCTAGCCACGGATTTACGACTTTGGCTACGA
AAATTTGACTTCTACTTGTAGTACGAGAGTTGGAAAGAAGCATGGGGAAGATGGAAGAAG
GAACCATGATGTACCAGCCACATTGATGCAGTTTATGTATGTAAAGCTCGTCATGTTGTT
GCCCATAACATACGTAACGCTTCCTCTTGAGGATTAGATCTCTTATAGCATTTTCATTTT
TATTCGAAACCTTCTAGGTGTTTTTGCTTCACCCCGATCATACGCACCTTTTTTTCTGCA
TATTATTTTTGTTATTTTAAACATACTAGAAATTGAGTTGGTGCTACAAAAATAGTATCC
GAATCTTGAGTTTTTTTTTGTTTTGTTTGCCGTGATTCAACTCTTAAATAATATTTCATT
TCCGCAACGTAAATTTGGTAGTCCGATCTAACTATTGAGTTGGGGGCCGGTTTTTCTCTA
GAAAATATATAAGATTTTCTTTTGACATACTATTCGTTCATTTAATTGGACAATATAATT
CATAGTTTTATCGGGGATCATTGCACAGTAGTTTTTTTTTCCCTTATCATTACACGGGAG
TTGGATAACTCACACACACATATATATATATATGTTCATTTTTGAATAGCAAAACCAATA
TTAATTATTTTTGCCTTTCT
>AhPDF1_1b
GATGATTATTACTACTTAGCTTTATTTTCAATGTAGATATGTATGTGTTGTGTGAAGAAA
TAAGCTTAAGATCAACCTCTATTTATAATGAAATGATGGTGAGTTAATCTTCTACTTGTG
TACCGTACAATAACTTGGTAACCCCACAAATTAAACACACCGCGACTTCTAGTAGCTAAT
ATTGAAAGATAACATCTTGGATATTATTCATAGTTGATGGCTGGTTTCTACACCACATAT
TTTTCCCTGTCAATATCTAATGTAACCTAGGTGGCTGCTTTATCTTTGAGCTAATGCTAC
AACCAGCCCGGTCCTTTACCAATTTTTATGAAACATGTGTTGGGCTTCATATTTTTTAGG
CCCATTTTGTTGTGAATTTTTTTTTTTTTTTTTAGGTTTCAAAATTAGTAAATAATCTCC
CAAAATTAGTAAAAGACCCTATAATTAACAAAAAAAAATACTCCATAATTAGTAGAAAACT
CTAATAATTAACAAAAAATGTCTAAAAATTAAGAAAAATTATGAATATCTCATTTTTAGT
AAAAAAAAAAAAAATCAAAATAACAGTTTTTAATTTTTTAAAAAGCCCAAAACAAATTTT
CGTATTTGGCCCCGGATTTTACCGGACCGGCACGGGCTACAACAACATGGGCAGCTGCTT
AATCTGACTTGGTCAAAACTCAAGAAAATAAGTATATGAATATGGATATGTTAGAGATTT
TCATAGTTAAGACAAAACAGCATGGTTCCGCAAAACCGCTGTCCCCTGATTTCGCCATCA
GAATGGGACGACTTTTTACCGGAGGAACGGAGAGGGCAGGGACTGAGAAATAAGCTTAGT
TGGCTATGGACAAACAAAACCATTGTTAGTAGTGGCTAAACTGAAAACCGAAGCGGAAAT
AAATACCGAAACCTTTGCCCGTAATGGTCTGAACGTAATGGGCTAAGTGAGAGGCTCAGG
ATAATTAAAAAAAAAAATAACGGTGAATTTCATCAAAATTTGGTGGATTAGCTGGCTAAAG
GTCTAATTCGTACATTTGTAACAAAAGAGTTTGGTAAAATAACTATAAAATTAATAACTG
ATGAAACAAATATCATTAAGGACAACCTGAAAGATTATAAGAGATCAAAATTAAAATATC
TATATATATATATATATATATATATATATATATATATTTCTTTTAAAATCTTAGGAAATTTA
GATTTTTTCTAGGATTTTTTTTTGGAGGATTGATTTAAAGATTTTGGATTTAAGCTGAAA
TTTTTAGAATTTAGGATTTAGAATTTTTTTTATTAACTTCGAAATTTGTATTAGGATCTT
AATTTTAGTGAACCCGGATGCGGGCTTTACACTTATCTTCTATGGGAATATCCTTTTCCC
CAAGTGAACTAGAAGCAGGAATATCCTTATCAGTGTTTTTGTTCTCTTTCATTAGGATTC
ATGATTATAAATTTTGAATTTAAAATATATCGTCTTCTATTTGAACATCGTTCAATCACG
TGGTAAGTGGTAACTAGTAACCCCACAAATTAAACACACAGCGTCTTCTAGTAACTAACA

```
ATATTAAAAGATAAGATCATCTTGGTTGATGGCTGGTTTCTCCACCTCCACCACATATAT
TTCCCAATGGGAAGGCTGCTTCTCTCTATCTAATGTAACCTAAGTGGCTAGCTGCTTTGG
CTTTACCTAAGTGGCTAGCTGTTTTGGCTTTTTGAGTCAAGTATTATATCGTTCACATAG
GCGGCTAATTAATTTGACTTAGTCAAGAGGTTTATACGGTTCAATGTTTGGAAGGAAGAA
GACGTGAAAAAATGAACCATGAATCATGATGCCTCTCGCGGCACTGATGCGGTCATTTTT
GTAAATGTCGTCCACATTTTCTATAACATTCTTGACACTTCCGTTTGAAATTTAAATCTC
CTATTTTATTTCATTTTGATCAGAAACTTTTATAATTTATTACTCCCACGCACACAAAAA
AGGTATCTTATATATATATGAAAGAATTC
>AtPDF11
GACTATTGTTTATACTTAGTTGTTTTCGATGTATATATGTTTTAGTTTGAAGAAATCTGC
TTGAAATCAACCACTATTTATAGTGAAAAAATGATGGGGAGGTCGACTTTTACGTGTGCA
TGGGACAATAAAGCGTTCAACCCTATAAATAAAAAAATACACACCGTCTGATAGTAGAGA
ATACTAACATGCAAGTATTCATCTAATGATAGAAGATAATATCTTGACCAATTCATAAAA
AGTGTGCTGATCGTGATCGGTGGCGAGTTTTCAGGTGCTACGTTTGTTCATTTGCAATTT
CATTGGACCGGTGGCTACTACATTAACTGTTAACATCTCTGGCTGGTTAATTTGACTTGG
TCAAAATTTTAAATATGTTGAGCATATCCTAGTACGGTACTCAAGTGGTACTTTGTCTCG
ACCACATCGCATTTAATAATAAACCATGAAGTTGGGTAGTATTTTTTTATCGACTTTTAT
TATTTTTTACTGAAACCTCCAAGGAGCTACTTCACTAGCTGGCAAGTTGATAAATTAGGT
TATTTCTTCACTTTCTTTGGTATTGATTATATAATCATCAAACTAATTAGGATCTAAACA
TGTAATTAAATATTAGCAACATAGTGGATTTGGACTAACAAATTTGTAAAATAGCGCATA
GATTATATTTAATTTCGTAGTTCCCACCTCAACTCCCACACAAAACTTCTTGGATTCAAC
TCTGCCATTTTCGGACGACCGAGAAGATAAATCCGATTTAATTTTATTTATTTAACTTAT
TGTTTTAAGGTAAAAAAAAATTTTTAAGAAAAACTGAAAGAAAATTCGTTTTCGATTTCA
TAGCCGTCATCCGCAAACTTTGACAAAGACTTGTCTCCTCTTTAAACATTTTCTGTATCA
ACAGCCACGTACCTTCAAGCCCTTCTAAGACAGCACCAGCACTAGCCGTGCATGGACAAG
ACAAGTAACAAACTCAGAATAGTACACCACCCAAGTTAATTTGTTAGGTTATTATTAAGA
ATAGAAAGAGAAGTTAACATTGTTAGATATGAATTATATTTGGTTAGATTAAGAATGCTT
AACTTAGGGAAGGCTGCTTCTCTGTTTAATGTAACTTAACTATTTAAGTGGCTGCTTTGG
CTTTTGAGTCAAGTGTTACATTGCCTATAAGAAGTGGGCTTATTTTGAATACACACACAA
TTAAGGGATCAATTATGGGATTAGCATATTAAAGTCTGTGCTTTTGTAGATATAGGAACA
TCTCATGTTCCTTACAAATTTAGATACTTTTATATTATATGGATGACTGTTTTATCCTTC
AATCTAATTGAGTTTCTTCTCCTTGTACCTTCAATCTAATTGAGGAGAATTCATCAATAT
CAATCGATCTCAGAAATGTGCGATTATTTCTCTCTCTTTCTCTTTCTCTAATGGCATGGA
ATCTATTGCCTCTAAACTCACCATTGATGTCCATTGCTTCTCCATCACCTTACAAAATTC
AGATCTATGCCTGGCCAATCTTCTTCCTCGACGATGCAGGTGGTAATCCTCCATAGCTTT
TTTGAATTTGAATTGTTTGATCTGAATTAATTTCATCCTCTCTTTCTTCAATTTCAAAGA
GGGATAAGAAATAATTGAAAATTTTAATCGATTCCAATTATTTAAGATTAGTATATTTGG
GTTCGTTATATAATATTTTCTTAGGGGTTAGCTTTCTTCCATCAAAAAGCTTTTAACTGA
TTTTATTGGGGTTTATCTGAATTGGATGGTGATGAAATGTTATTAACAGATTTTCTAATT
TTTGTCTCTATCGATTTTGTTTCCGTAAATACATCTTTTGTGTATATATTGTCTTGTAGA
AAGAAACATTGATTAAGTAGAGAAATAGGATAATCAATCTCTGTACTTAATACATCATCC
ATTGAAACAAGTTAGGAATCGATCCAATATGAGGCTCTGCAAAAACAATTCAGGAAAAGA
TTCTGCAAAAACAATTTAGGAGAA
>AhPDF1_2a
GACTATTACTTTTGGTTTACAATTAATGTTGTATGTATTTATGTGTAGTGTGAAGAAATA
AGCATAAGATCAACCTCTATTTATAGTGAACTGATCAAAAGTTAGTGATCTTACATGTCA
CCATGCATGTCAACACCACGTAGAAACACAGACTGCAAAAATCTAGTAGATATTAAATCG
TAAATAGATAATAATAGAAGATACGATGTTGACTAATTTCATAAAAGAAAAAAAGAAGGA
AAAAAAAAAGAAGAAGATGTCGACTAATCAATAGTTGAAGGCTGGTTTCTCTACCACTTA
TCTTTGCATATGAAAGGCTGTCTGATCTATCTAATGAAACCTAAGTGGGCTGTGGGCGGC
TGGGTTGGCTTTGAATCGTTCGATTGTTCACATGGGCTGCTGGTTATAAATTTGACTTGT
TAAAGACTAAATATATAAGATAAAATCTTGACCCTAGGTCATAGTAACATAAGTGGTAGT
GATTTGCAGAGGAAACTAGAACTTCAAGGATACTATAATTGGCCATATGCTTGGGTTGCA
AGAAATTTCCAGATATGATACCAGTTTATAATCTTCTTCTCTTAAACTAGGTAGTATATA
TATATTTCATATAAACTATTTATCCAATAAGATGAATCATCGGTGCAAGTTAAGGTTTTA
AAAAGTGAAACTAGATCGGGTTGAATTTCATTTATTACAAAATTAATAAATTTTATGTTT
TAGAATAAAGTTGTAATATATTATAATAGTAAGTGAAAAAATAATATTTTTTTGTCAGTC
CCCATATATAATATTAGAATGAATATATTTTGTTTTTTCTATAGTATTCAATTATGAATT
TTTTTTTAAGATATGAAAACGGAAAATAAGATTATTTAAAAAAATAATAATATTGTTTGA
ACAATGAGTTAATTTATGTTAACAACCACAAAATGAGAGTGCATATAATTTAGTTAGATC
AAAAAATGAAAATATCATAAATGAATCGCATCTAAAACGTAGATTATTTTGAATTACTTA
AGAAAATTAGCTATTATTTTGAATTAATTTAGGAAATTATGTTTTCAAATTCTGATTCAT
TGGTTGTTGAAATAGAGATTTGTTTGAGCTAATTACGGAATTTACCAATTATTCACATAA
TCAAATATGTTATGAACATATATTTGGTATATAATAATATCAAATTCAATCCGTGTGTGG
AGTCTATATTTCATTTTACAATCCCTATGAAAATATTGCGGCATAATCTATTATTTATT
```

ATTTTCAAAATTGCTTGCAGAATCAAATTTTCAAATATTTAGAAGTGTCAAACCTTATTT
AACTCATCTCATCATAAAATCTTAAAATGCCGTCTGTTATAAAGATCATAACTTTCTACC
ATATAATACATTACATTTAATATGCCGCCTACTATAAATATTTTAAACATATTTTTTTAG
AAAATCAAATTAATGTATTTATAATTTTTTTTATCATTTTAATCTATTAAATATTTATGA
TATTCTTTAATATATTAAATTAAACTTTTTAAAAATTTTCCAAATTAAAATCTTTAAAAA
ATTTTATTGTAGTATGTAAAATTATGAATTTTAAAAAAATGATATGAAATTAAATATAAA
TATTCAAATAGTTATAAGTATCATAACTCATTTCATCATATAATATATTATATTCAACCC
GCCGCCTATTATAAGTATTTGTTTCAAAAAATCAAATTAGAGTATTTATACTTCATAAAT
TTTTGATCTATTAAGTATTGATGATATTAAAAAAAAAGGATATATTACTCAAATATAAAT
TTTTGACCCGTTAAATTTGTTCTTTCTTACATTGAATAATTTATATTCTTTTTTTTTTTG
TCAACCTTGGGCCACAACTGGCCGGCCCATTAGCCTAATCCCTACAAATTGTAAGGAGCG
GGACTCGATCCCTGATGTGATGGTGCCTGTCCATCAGTGGACTTATCATATGCCACTAGA
CTAAGGTCACTT
>AhPDF1_2c
GATGTTTATTACTACTTTGTTTTCAATGTATAGATGTATGTGTTGTGCGAAGAAATAAGC
TTAAGATTGTTAAGATCTATGGGCTTCCAACTCAAAACCAATTGGCAATGAGTGGAGTGG
CCCATATCCCTTATATATTACTTAGGATCCCTTCCAACTTCCGATGTGGGACATTATGTC
CTAATACGTCCCCTCGAGATGATGGCTCTTTGGCCATTAATCTCGGTATGCTCGGGCATG
GATCGGCGGGCCAACTATTGGGCCGGTCCGATGGTGGATTGGATTTGGACATGTACGGAT
CGGGCTCTGATACCATGTTAAGATCTATGGGCTTCCAACTCAAAACCAATTGGCAATAAG
TGGAGTGGCCCATATCCCTTATATATTACTTAGGATCCCTTCCAACTTCCGATGTGGGAC
ATTACGTCCTAATAAAGATCAACCTCTATTTATAGTGAAATGGTAGCGAGTTAATCTTCT
GCTTGTGCATCGTACAATAATAACGTGGTAACCCCACAAAAAGAAACGCACATTGTCTTC
TTGTAGCTAATAGAAAAAGATAAGATCTTGTTTTTTGATAGTGGATGGCTGGTTTCTCCA
CCACATGTTTTTCTCTGTCCATATATATAATGCAACCTATAAGCGGCTGCTTTGGTTTTT
AGCTAATGCTATATCGTTCACTTGGGCGGCTGGTTAATCTGACAAGGCTAAAGAAAATAA
GATCTTAACAAAGCCATAGTTTTTTTTCTTTTTCTTTTAGCTAATAATATCTTAACCAAT
AACCAAGCCATAGTTACACAAGTATATTGATATGTCAGAGATTTTCAAAGTTAAGACAAG
TGTAGTTAGTAGTCTGTCCGTCGACGAGTGAATACCACGGAAGTAACAGTGTAAGAGATT
TCTTTGCGCACACATTCTGAAAGGTGAAAACCATATGAGTAGAGCGATCAGATCAAAGTT
TGGTTGATCTCAAAAGCAGGATCGTTCCGCCAAGTTTGCAGAACAAGGAACTGCTCGCGA
AGTTCGCGCCAACAGTAGGTTTCAAACCGTCAGAAGGAGGAATGAAGAGGGCAGGAACTG
AAAATTTAGCTTAGTTGGTTATAAAATAAACAAACTATAGTTGGTCGTGGCCTTGAACGT
AATAGACTATCGACATGGTCTGAACGTAATTGGTTAACTTGGCCTATTTTGGTTAATGAG
CATTAGTGACTAGTGAGTAGTCTTTTTAAGAACTTTGGGCTTAATAGTTAACTTGTGATG
TTTAGGGGGTAGATTTCGTACTTTCCATGTTTATGGGATGTTTTTAATGCTAGATTAATA
ATGATTAAATCGTGCATGATTTACATTCAAATTAAAATTAAATCGATTAAGATTGACCAT
AACATCTAATCTCTGTTTTTGTTCAACTATCAAATTAATGTTAATGTTCCGTTAGGGTTT
CGACTTTGGAGATTGGTTTCTTGAAGAAACTGTGGTTTAACAACGATTAAACACCTAAAT
CTTTGTTTCTAGTAATGGTTTTTTTAATTTAGCCTCACTTGCTTGCGCTTGAAGAAACTT
GTTTTACTTGCACACGTCAATCATGAAAAGTTTATTCAACACACATAAATCACAATCACA
ATCACACAGATAAGTTACACACTTGAAGCATAATTATTCAACACACAGATAATTTCACAG
AGAAAATAAAAGACTCATAAAATAGAAAACTTCAAATGATATCTTTAGGTAACTTTGTTC
AGCACCGGAATGCTCTGTTCATCGCAGAAGAAATCTGTTGGATCAACACTCGTTTTCACT
CTCACCAATCTCTCGAAATTGTTCTTGAAGTACTTCACTCCCCAACTCTTTCCTTCCTCG
TACTTTGTCTTCTTCTTTCCAAGGTACATACCCAAATCCATATCCATGAAATTGACATAA
GCTCCTCTTGGAGATTTCGAAACATATGGAGTCATAAGTTCGTAAACGCTATCGACCCAT
TTCAGATATTTATTTGTCCCCGTCATGTTCTTGTCTTCCTCTTCTCTCCAGTAAGCC
>AtPDF12a
GATGATTATTACTATTTTGTTTTCAATGTATAGATGTATGTGTTGTGTGAAGAAATAAGC
CAAAGATCAACGTCTATTTATAATGAAATGGTAGTGGGTTAATCTTCTACTTGTGCATCG
TACAATAACGTGGTAACCCCACACATTAAACAGACATCATCTTCCCGTAGCCAATATCAA
AAGATAAGATCTTGCTTTTTGTTTGTTGATGGCTGGTTTCTCCACCACATATATTTCTCT
GTCGATATCTAAAGTAACCTAAGCGGCTGCTTCGGCTTTTAGCTAATGCTACATCGTTCA
CATGGGCGGCTGGTTAATCTGAATGGATTAAGACTCAAGAAAATAATATCTTAACCAAGC
CATAGTTACACAAGTATATTGATGTCAGAGATTCTCGCGGTTAGGACAAGTGCAGACTGT
GCAGTTCATAGTCTGACCTTCGTCATCGTCGAACAAATACAACATTTTTCGATTAGAGGA
TCACCCCTAGACATATAGATCGTTGTGGACTAAACATATGGTTTGGTTGATCTCAAGAGC
AGCATTGTTCCGCCAAGTTTGCAGAACAAGGAACCGTTCTCGCGTAGTTTCGCCATTTAT
ATATCCCTGGGACACACCTATTACTGAAAATTGGATGATTCATCAATATTTTGCTCTCAC
CGTCGGTAGGTTTTCAAACTATCGAAGGGACAAAGAAACGATTGGTACCGGAGGAACGGA
GAGGGCAGGAACCTAAAAGTTAGCTTAGTTGGCTATTGATAAACAAACTATAGTTGATTG
TGGCCTTAAACGTAATGGACTATCGACATGCATGGTCTGACCGCAAGTGAGAGGCTCAGG
GTAATTTAAAAAAAAACATTAACGGTGTTGTCAGTCATCAAAATTTTGTAGATTAGCTAT
AAAGGTATTATTCGTAAATTCGTAAACAACGACATTCAACAAAATAACTGCAAATTTTAA

```
CTAACTTAATAAATAAAAAAGGAAAAACCGAACATTATAAGAGATCAGAATTTAAATTTG
TAAAAGTATAGATTAATTCTTTTTAAACTTGGGAAATACATATTTTTGTAGGGCTTAGAA
CTTTTGCTTTGGAATGAGGATTAAAATTTGGGTTTAGATTTATGATTATAAATTTACATA
GGCAACATGATATATATTGTGAGTTAACCCCAAATTTGGACATGAAAGTAGAAACGATGC
ACCTCGGGATATCGATGCGGCGATGCATGCTGCATATAAAGCTTGTCCCAATGTCCATAA
AATTCTCGACACGCCCGTTTTACATTTATAAATCCCTATCTTTATCATTTTGATTTAATA
TTTTTTTAATATGTTCAAGATTTTCACCCCAATCATGTATTTTTATGGGATATGATGAAA
CAGTCCAACGTCGTACATAATTTTTTAGTATTATTATAACAAGGAGAAACCAAACATAAA
AACATAAACATATATGCATGTCATAAAGTCACTCATAGAGTGACACCGACTTATTTAAAG
TAAAATACACACACGATCAAGCACCAAAGATTTTTGGTAGA
>AtPDF12c
GACTATTATACTTTTGCTTTGCAATGTATGTCTTTTGTATGTTTGAATGTGGGATGTGAA
GAAATAAGCATAAGATCAACCTCTATTTATAGTGAACTGATGAAGAAGAGTTAGTCTTTT
ACATGTCATCATGTCAACCCCACGTAGTAAACACAGACTGTAATCTATAGTAGATATTAA
ATCGTAAATAGCTAATAATAGAAGATATCATGATGTTGACTAATCAATACTTGATGGCTG
GTTTCTCCACCACTTATCTTTACATATGAAAGGCTGCCTGATCTATCATCTATCTAATGT
AACTTAAGCGGGAGGCTGCGTTGGCTTTGAGGTAATGCTTCATCGTTCGATTGTTCACAT
GGGCTGCTGGTTAATTTGACTTGTTCAAGACTCAATATATTCTATTTTCGATGAAAAAAA
TATAATTAAGAAGGTTATCTCTGATTTTTTTTTTTTTTTTTTGCAAGTGAGCCCAAGTCT
TATTAACATAAGTTGTAGTGATTGGCAGAGATTTTCCGACGTTCGTTGAATGAGAGATTA
TAAAGGTAGAAGAGGAAACTAGAACTTTAAGGATACCAGCCCCGCCTAAAGAAAGACAAA
TTGGCCTTTGGGTTAAAAGACAATTGGGCTTTCTATCATCAAAATGTTTTTCTTAGAACT
ATTTTTAATAATGTTTTTGATTTTTTATTATAAAAAAATAATTTCAAAATTATCACTAGA
CACACAAAAATAAAAATAAGAGATGGTGAATTACAGTGTTCCGAGTACATTATGTCGACC
TACTTAAGATAATGAATGATTTAGATTAAAATTAAAATTAAATCGATTAAGATTATTACC
ATAGATAGATCTAAGACCAAATAGAGGAAGAAGATTCATACAGGTCAAAACATCTAATCT
CTGTTTTTGTTCAACTATCAAATTCCGTTAATGTTCCATTAGGGTTCGAGATTCGTTGCT
TGACCTAATTCCAGCATAAATGTTTCTTGAAGAAACAGTGGTTTTAACGAGGAGAGGATT
AATTACACACCTAATCTTTGTTTCTGTGATGTCTTTTTATTTAGTTTCACTTGCTTGCGC
TTGAAGAAACTTGTTTTTAGTTACACACTTCAAACATGAAAAGTTTATTCAACACACATA
TCACAATTCACAATCACACACATAAGTTACACACTTGACGCATAATATTCAACACGCACA
GATGATTTCACAGAGAAAAGGGACTAATAAAAAACTTCAAATGATATCT
>AhPDF1_2b
GATGATTATTACTACTTTGTTTTCAATGTATTTATGTATGTGTTGTGTGAAGAAATAATC
TTAAGATCAACCTCTATTTATAATGAAATGATGGTGAATATTTTCTACTTGTGATCGTAC
AATAACGTGCTAATCGTACCCCACAAATTGAACACACCATGTATGGCTTCTCGTAGCTAA
TATTAAAAGATAAGATCTTGGGTTATTAATAGTTGATGGCTTCTTTCTCCACCACATAAT
TTTCTCTGTCAATATCTAATGTAACCTAAGCGGCTGCTTTGGCTTTGCTACATCGTTCAC
ATGGGCGGCTGGTTAATCTGATTTGGACCAGACTCAAGAAAATAAGATTTTTAACCAAGC
AATAGTTACACAAGTATATTGATATATATGTCAGATATTTTCACAGTTAAGACAAGTGTA
GTTGGTAGTCTGACCTTCGTCGAATAAATACCTTGGAAGTAACAGTGTAAGATATTTCTT
TGCGCAGATTTTTTGAAAGGTGAAAACCATATGAGAAGAGCAATCAGATAAATCTAAGCG
GTCAAAATTTTCGGATTAGAGCATCACCCCTAGACATACATCATAGTGGACTATCGACAT
GGTTTGGTTGATCTCAAGAGGAGCATCGTTCTACCAAGTTTTCAGAACAAGAAACTGCTC
TCGCAAAGTTTCCCCATGTAAATTTTACCAGGAAACACCTATAACCGAAATTGGATGATT
CATCAATGTGTTGCTCTCGCCGGTGGTAAATCTAGATTTTCAAACTAGCTAAGTTGGCTA
TGGATCAAACCATTGTTGGTCGTGGCTATGAATTTAGAGTACATAGGAAAAACGCTATAT
ATTGTGAGTAGTTCTTAATTATGGAAATTCAAACAGATGTTGATCATCCGAAGAAACAGT
AGTATGTAGTAAAAATACTATGTGGAGACATGCATGAAAAAAGGAACAATGCACCTCCCG
ATACCGATACCGATGCCTGATTTATGTAAAGCTTGTCCCATTGTCCATATATAACATTCT
CATCACTTCCGTTTGAGACTTAAATCTCTTATCTTTATCACTTTTATTTAATCTTGTTAT
ATATGTTCTTGCTTCACCCCAATCATGTCTTTATATGCATTTCATATGTTTGCTATAATT
GTTACTAGATAGACAATATTAGAAAATGCTATAAATTTTTTTTTTCTTTTTTGGATCTTT
AGTTTTTTTTTTGTTTTTTTTTACCGTAATTCAACTATCTCAATTAGTATATCACTTGTC
TAGCATGAATTTGGTAGTCTGAATAAAGTTGGGGTTTAATTTCACCTAGTGATTTAACGT
GAAAAATATGGACAAGAACAAAAAGTCCTACTTCGTCGTACATAATTCTTTTATATTATT
ATAACAAGAAAGCAAACCAAAACAAACATTGAAACATAAAGATGCATGTCATAGAGTCAC
TATGTAAAATGTTTGTTTTGGTTTGCTTTCTTGTTATAATAATATATGTTTATGACATGC
ATCTTTATGTTTGTGTCAAACATTTGACATGCATCTTTTGGTTTCCAATTTATGTATATG
TTTGTATGTATGTATGTATGTATGAGTAGTGTGAAGAAGTAAGCACAAGATCAAACTCTA
TTTATAGTGAACTATTGTAAATAGAAAACAATAGAAGATAAGATGTTGACTAATCAATAG
TTGATGGCTGGTTTCTCCACCACATATGAAAGACAAAGCGGGCGGCTGCGTTGGTTATGA
GATGGTGCTACATCGTTCGATATGTTCACATGGGCTGCTGGTTAATTAATTTGACAAAAT
CTTGACCTAAGTCAAAAATATGTTGTAGTAATTTGCAGAGATATTCCAACGTTCGTTGCA
TGAGAGTGTATAAATATATTTATCCAAAAGATGAATCATCAATTTATTTATTTTTTCTAA
```

TGAGAAGGTGTTCCGGATTTTTACCATCCAACTAATTTCTATGGTGGGTATAGTCGTCAA
GTAGCCCACTCACCGGATTTTCCAATGGGCCTAAAACATGACACCATTCA
>AtPDF12b
GATGATTATTACTACTTTGTTTTCAATGTATGTATGTATGTGTTGTGTGAAGAAATAAGC
TTAAGAGATCAACCTTTATTTATAATGAAATGATGGTGAGTTTATTTTCTACTTGTGCAT
GGTACAATAACGAGCTAATTGTACCCCACAAATTAAACACACCACGTACGCCTTCTCGAG
CTAATATTAAAAGATAAGATCTTGGCTTATTAATAGTTGATGGCTTGTTTATCTACCACA
TATTTTTCTCTCTCAATATCTAATGTAACCTAAGCGGCTGCTTTGGCTTTGCATGGGCGG
CTGGTTAATCTGACTTGGACAAGATGCAAGAAATAAGATCTTAACCAAGCCAATAGTTAC
ACAAGTATATTAAATTCAGAGATTTTCACAGTTAGGAAAAGTGTAGTTGGTAGTCTGACC
TTCGTCGAATAAATACCTTGATAGTAATAGTGTAAGCTATTTCATAATTTGTGCAAAATT
TTTGAAAGGTGAAAACTATATATATGAACAAGATAAAACTAAGCGGTCAAAATTTTCCGA
TTTGAGCATCACCCCTAGACATACAACATAAGGGACTATCGACATGGTTTGGTTGATCTC
AAGAGCAGCATCGTTCAACCAAGTTTTCAGAATAAGAAACTGCATGCTGTCGAAGTTTCC
CATTTACATTTTACCAGGAAACACCTATAATCGAAAATTGGATGATTCATCAATCTCTTG
CGCCGGTGGTAAATTTTCAAACTAGCTAAGTTGCTATGTATAAACAAACCATTGTTGGTC
GTGGCTATGAATTTAGGGTACATAGGCAAAATGCTATATATATATATATATATATATATA
TATATATATATTGTGAGTAGTTCTTAGTTATGGAAATTCAAACAAATATGGATCCGATGA
AACAGTCGTATGTAGTATAAATACTATTCGGAGACATGAAACAAACTGTTATCGATGCCT
GATGTATGTAATGCTCGTCCCATTGTCCCATATATAACATTCTCGTCACTTCCGTTTGAG
ATTTAATTCTCTTATCTTTATCATTTTTATTCAATCTTTTTATATATTTTTCTTGCTTCA
TCCCAAATTCCCAATCATGTTTTTATATGCATTTTATGTTTGCTATTATTGTTACTTGCT
AGCAAGACAATATTAGAACATATGCTATAAAAAGGTAATTTAAATCTCTAGTTTCTTACC
GTATATATAATTCAAAGTCTCAATTAATATTTCATTTGTCAAGCTTGAATTTGGTAGTCT
GATCAAACAATAAATTGGGGTTTAGTTTCACCTAATGATCTAACGTGACAAATATTGATA
GAAGAAGAAAAAACTTCGTACATAATTCTTTTATTTTTATTATAAAACCAAAGCAAACGA
AAAAAAGAAAACAACAACAAAGCAAACGAAACACTAAAACATAAACATGCATGTCATAAT
AAAGTCACTCATAGAATGACAAAGACTTATTTTATGTAAAATACACACCATGAAGCACCA
AGTATAATTGGTAGTCATTGGTAGC
>AtPDF1_3
GATTATATTACTTTCAGTTTCCAATTAATGTATGTTTGTATGTATTTGTGTGTAGTGTGA
AGATATAAGCACAATATCAACCTCTATTTATAGTGAACTGGTGAAGAGTTAGTCTTTTAC
ATGTCAAGTAGATATTAAATCGTAAATAGCTAATGATAGAAGATAAGATGTTGACTAATC
AATAGTTGATGGCTGGTTTCTCCGCCACTTATCTTTACATATGAAAGGCTGCCTGATCTA
TCTAATGTAAGCTAAGCGGGCGGTTGTGTTGGCTTTGAGAAAGTGCTACATTGTTCGATA
TGTTCACATGGGCTGATGATTCATTTTTCTTTTCTAAGAATGTAAATATCATTAATCAGT
GTACGTTTACAGATTTTACAGATTGCAAAACCAGTTTTAAAGGTCCAAAGTAAACATGAA
AGCAAAAACAACTCAAACTTCAGTGCATCGACAAAACTTTAAGCTATGAATCTTATCGGT
GTCGAGATGCAATGATAACGATTTTGAAAAACCGGGAAACTGGAAAAACCGTGATATACA
TAGCCCATAAAGGAAAAGCAACATGAATGTCTTGGTTATATAACTTCGCTAAACGGAAGC
TCTTTCACAAGCTGCCTGAGGGATTCAAAACCTTCACTCCAAATGATATTCAATGCCCTA
CGCATTGCCTAGCATTCGTGGCTCATCCAACTAAACCGAGATCAAGCACAACTTCTCGAT
TTAAACATTGGTGAATGGCGGACAATGCCGAGTCCGCCTCCTTACTATGTCGAAGCCAGA
CATCATTCGGTCTTTGTGAATGGTTCCATCTACTGGTTCGAAGTACTTAATGGTTACAAG
ATTCTATCTTTGGATCTTCACACTGAACAGTTCCATGATGTGTCCCAAGACCGCGCGCAT
TACCTACACCTGGCCGCTAGTCAACCTTGAAGATCTTCTAGCCATGGTTAGTGGCAGTGC
TGAACTTTCTGATTGGAAATTAGCCATATGGACATTGATACACAAGAAGAGACATGGACC
AAAACTTACTCAATGCGTTTATTCCCTGGATATCTTTGCCCCAACTATGTGACGATGAAG
TGCATTCCACTTACAAGCCTTATCCTCCAGGGGAGGTGGTACAGATGTATTAAAAGGACC
CTCTCATATATGTTTCCTTTAACTTAGTTTAAGAACACAATTTTTTTGTTCATGCACAAA
TAATTAGTTTAAGAGTTTAATGCGTAGAGTAATCCAACACAAAGCGTGAAAATATGTAAC
ATAGGATAATTTTATTTTATTTTGCTTTTAAATAAAAAAATAGCGCATAATTATGTGATT
GCATAATCATAAAATATATATATTTTTTAATCGTAGTTACAAATTATTAAAAATTGCATGAA
CATAATACGGATACCCAATAATTCTACATGACATATTTGAAAGGTTGTTGTTAGAAGAGA
AAAAAAAAAAAAAAAAAAAAAAAGGACATAGTTGAAGGGTTGTACCGCATGATAATACTTA
CGACTTACAAGTGGCAATGAAGCAACGTGGTAGCAAACGAGGCCCATGGTCATGTGGCCC
CCGGATGCGTAGATCAACACGAACATAACATAGCACCACCAATGACAAATAGAACAACGA
ATGGAAAACAAAAGCATTTTTCGCTTCAAGCCAAGTGTTTTATGTGAGAGTCCAATTATG
TCTTTCTTTCAGATGGTGTCTACTCTCTTTATAGCTTGCACCTTCTTCTTGTGATCTTGT
GTGTTAGTAGACACCATCTGAAAGAGATAATGGACTTCTAATTCCAAATTGGTACAATAT
GGGTTTCAGTCATTTAACTTGGATTAACTTTCAAACCCATAGAATTTGTTTATCATAAGT
ATCATTGACTCTTGAAATTAGTGCTTCAAATGTAAAACTATAACTAACCGGTCTAGTGAC
ATGAAAAATAATTGAATTTGATTATAAACGAAAAGAGGAAATTAGAAGATAAAGCCATAA
GATGGGGTAAAGTCTTATGAAACCTCCAAC
>AhPDF1_4

GGCTTATTTTGGTTATATGGTAATATCCACTAGCTGAGAGCTCTTTATATACAAATGTAT
ATGAAAAAACGTTTAATCTTTGAATGTAAAACAATTATCGTTCTCTAGAACTCTCTTTGT
GTTTTGTATAGTGTGAACTGGGAGAAACTATGAAGCCTACTATTTATCCAAATTCTTATC
CATACAAGTTTTGAATATAGACTTTGTGATGATAGACTTTTGACGTCTAATTTGTTCGGA
CATGTTACTACCGTACACAAATGTGTGTATATATTTGTTCAAATTTTATGTTTGCATTAA
AAAATGTTAAACACAATGACTAATTAACTATAAACATAAATGAAGACACAAAATTATGAA
AGTCCATATATAAAAATTGTTCGAACGAAAGTGCACGGTGGTCAAAACAGGACATGAATC
TGGCTAAGCATTACAAATTATGTTGAACCTGCATGTACCGGTATCTAAATTACTTTAATT
TTTTTTTTTACGATCTAATTATTTGCTTGATATTGTCGGAATATATAAAAAAATGGTATG
ATTATGACAACGATTGTTTGATCGCGACCACTGAATCAGATAGTGTTTGGCTCTGCACAT
ACATAAATTAAAACCAGTCTTGTCTGTAATTTCTTTTAGGCTACTATATTTTCTAAAATA
AATGGATTCAACCTTGATCGTTGAAAATGTTACATCTCCATCTTTATTATGTGTGTCAAC
TGTGAAATTGCTTTTAAGCACGTGACTCATCTCATCTCACGTGATTCTCGGATCTCGACC
ATTTTTCTTGATTTTCCGATTTCCATGACCCTCGTGATCTCTATACTATTAAAAGAGAAG
TACAAATGAAAATGGGTCGGGTTAAAATCAGAAAATTGACCCGAAATTATGAAATCGGAT
CTTTGAATACTATTAAAAGAGAAGTACAAATGAAAATGGGTCGGGTTAAAATAAGAAAAT
TGACCCGAAATTCTGAAATCGGATCTTTGAATTTTTTTTGGAATATAAATTCGAATTAAT
AATGATAAATCGAATTAAAATCAAATATTTATGGAAATCAAATAAAATCTGATTGTACTT
ATGGTAACTAATTATAAAAGCATATTCCGAACAATATTATACATTAATTACGTATAATAA
ATTTTTCATATATTTTTGGAACTAATTCCATAACAATCAACGTTTTTGTCATTAATTTCG
CTCATTTTAGCCAATTATCATTAATTAAAACATATCTTACTTGCTTCTAAAGATTTCTAG
AAAATTTATCACAAATAACTCATCTGTTTTATACAACGCGGTTATTTGAGATTTCTAGAT
TACTTGATATTTTACTACAAATAACCCATCTGTTTTATGAAACATATCTTACTTGCTTCC
AAAGATTTATAGATTACTTGATATTTTTAATTTAATTAGATTAAATCAAAATTATGGTAT
TTTCATACCTATAGCTTACCAAAGAAGTTTAATTTTCAATTTATAAAGATTTTTTGAATA
TCATTTTAGAATTATTCAACAAGTTGATTAGTATTTATTAAATTTTGTGAATCATGAAAA
TTAATAATTATATTTTAATCATTATATTTTATCTTTCAATCTCTCATCTAACAAATAAGA
TACATATTCTATTATTCCTAAGTCCAAACTTTATTTTGTTGTATGACTAAATAATATTCC
CATTAGAGCTGTCAAACCGAGTTGATTCGTCCCGTTAAGCCCGTTTTGCGAAAAGGCAAA
AAATATTTTGACTTGTAGTTAGTCGGTCCGCACGACCCGCGGATAACATATATGAAATAC
AAACCCGTCCCGCGAATGTATGCGGATTTGCGGGATTGTCCGTGGATTTTGTATAAATAA
TTTTAAAGTTACCAAAATTTAAAATACTACTAAAATAAATAAATAAATAAAAATACTATT
AAAAGTTTTTTTAAATTACCACATGTTGAATAAATTATAATTTTATCAATAAATACTAAC
ATGCGGGCTGATCCGCCAACCCGCGGATAAAAACCGATTAAACCTATGACCCACTGCGAA
TTAATCCATGTGGCCCGCTTATTTGCGGGTTTAATATATTTTCCCAATTCTGCCCCATAG
TGAGATGATTTGGGCTCGACCCGCGGGTTTTGACCTAAAGTGACTGCTCTAATTCCCATG
AATCGTCTAATTTTAGAATTC
>AtPDF1_4
GACTTTTTGTTGTATGGTTAATATTCACTAGCTGAGAGCTCTTTATATACAAATGTATTA
AAAACCCGTTTAATCTTTGACTGTAAAACGATTATCGTTCTCTAGAACTATCCTTGTATT
TTGTATGGTGTGAACAAGGAGAAACTATGAAGCCTACTATTTATCCAAATTCTTATCCAT
ACAAGTTTTGACTATAGACTTTGTGATGATACACTTTTGACGTCTAATTTGTTCGGACAT
GTTAGTGTTATTAGTTCAAATTTTATGTTTGCCTTAAACAAAAAGTTAAACGCAACTAAT
AACGATATATAAACGTAAATGAAGACACAAAATTATGAAAGTCTATAGTCAAAAACTGTT
CGAACGAAAGTGCATGGTGGTCAAAACAGGACATGAATCTAACTAAGCATCGCAAATTAG
GTCTAGCATGAATGTAACAGCTAAATTAATTTAATTTGTACGATCTAATTATTTGCTTG
ATATTTTTGGAAAAAAAAAATGGTATAATTATGACAAAGATTGTTTGATAGCGACCACAGA
ATCAGAGAGTGTTTGGCTCTGCACATACATAAACCAGTCTTGTCTGTAATTTCTTTTAGG
CTAATCTATTTTCTGAAATAAATGGATTCAACATTGATCACTATTCACTGAAAATGTTAC
ACTCCATCTTTATTATTATGTGTCAACTGTGAAATTGCTTTTGAGCACATGACTCATCTC
ATCTCACGTGATTCTCGGGTCTCGACCATTTTTCTTGATTTTGCGATTTCCATGACCCTC
GTGATTGTGATACTAATTCAGTAATTCTGAAGCATTTATGTGTGGATAAGTTTGTTCTTG
GCGCGTGACTTTCACGTGTCTGCTTAAAGGGGTTTACAAGTGGAATTTAGGCTTTATTGT
TTATTTATACATGCTCACCAACATCTATTTACATATACTTCTTGTTCTTCGCCTATGCAC
TACTACACATATCTTGTAAAATTTGTATATTGTTAAAGATAGGCATTGTTGCTTTGTAGA
AACTTAAATAGTTGTAGTTATATGTAGATGCAAAGGAACATTTTACTTTAGGAAAGATTC
AGAAAACAGCTCGAGTTAATTAATATCTACACCCAAATCTAGAAAACTAATTAATGAGGT
GTTTATATCGGAAACTTGCTGGATCGATATGAGATCTTACAAATATTCTGCAACCTAAGA
GTATATATCAATTGAACATGTCGGATATAATAGTATCTATATATGTATGTGTATATAAAA
TGGAAAAGTTCTAGTTTTATTAAAACCAAAAGATCTACCAACGCAAGCAATGCCAATAAT
ATAGATTGAAGTAATAATATATATATCTTGCAATGACATTTTAATTGACACTAAGTATCC
ATATAGTGGCCAAGGTACCTAGATAAAGAATTTATGTTAAACATCCACCATGGTAATTAA
CCTTTTAACCTACATGCTTCTATATATGAATTTCGATTATAAACCCTGTAATCTATGACT
ATATTTTATTATTATATGCACATACTAAAACACTCCTAATCAGAAATGTTATAGCGCAAA
ACATTCTTTCATAAAGTTAAAAAGAACCATATAATTTGCCTAACACATTAGCTAAAAACA

TAGGAAAATTGGGACATTATTGGTCGAGCGACCACAATATTAAAACTTTGAGATAGCAAA
GCAAAAACATTACTAAATGACCATTATGCAATCATATTAATTTATTATAAAGTCCCTATA
AAATGACCACACTGGTGATCGTATCCATCTCGGTATCAGTGTGTCTCTTTCTCCAACCAG
AATATTTTCTTTCTCCCATCTTTCCTTTCATTAATCCGATTAACATCCCAAGAACATTTA
TCATATATATTCACTTGCCCGTTTCATTTTCTCGCAACATATTCTATAGATTTCTTTCTA
ACATTTTCACATACATATCTATAAATACTAAAATACACCATAATAAATCTCAACAATTTC
AATATATAAAAAAAA
>AhPDF1_5Allele5F13
GACTATTACTTACTTAGATATCAATGTATTTGTTGTAATTAGCGTAATATCAACCTCTAT
TTATAATAAAATGATGGTGCGGTGAGTTTGTCAATTGTCACCAATTAGACAAGTAGATAA
TAATACTTGATACCTATCTATAATGTTGCCTGATCTAACCTAAGTGACTGGTTTGGCTTG
AGCTAGTACTACAATGTTCACTAGGGTGGCTGGATTAGTTGACTTATTGTAGATTAACAT
GGCAAGTAGATAAATCGGCATATGATCAAGGTCTTAGCAAATGAGCATGATTCCCCCTCT
CCATGTCCTTCATATTTTTGATACATGTTTTGGTATATGGTATCATAGAAGAGGATGGAT
AGTCACACGTGAGAATTGGCTTTCTAATTTGGTGTTTTGGTCTTTTTACGGTTTTGAGAA
TTTTTTCACATTAGGTTTTGATATTTACTTTTTAGACAATGTATCTCTCTTCTTAATAAG
AAAACAACTTCCAACCATTAACCTAATTCTCTAAAAAGGTTTGGGTTAAAGGAAGCTTTG
CTATAGCCTTTTCTGTTCTTAAGGTTTAAACAACAGCATCTACACTAGATCTGTGGCAAG
GGCTGAGGCTCGTATCACTTTTATTTCCGTTTAAATTTGCAATAAAAAAACTTTCTTTTT
AATTTGAGTTTTTATTTTTTATTTTTTTTTTGTCATCCGAAATTAATTAGAACTCAAAAT
AGCCATTTCTGAAAAATGTTGTTTAGATATGTGATTTGCGTTTTTATCATGTTCTGTTTT
TTAAAATTTTTTAATGATATATATATTTATTGATCTATTATCTTATGGGTTTTTCCTTTC
TAGTGAGCAGGATTATATTCATTATTTTTGAAAGACTAACTCCCAACAGAATGGCCCGTA
AAACTGAAACTTAGCTCTATCTTAAATGCTCTGTTATTTCGACTAATAATTGTTCGTGTA
TAATAATCAGAAATGACAATAATTCACACATAAGAATCAGTGTTGAGAAATTCACAACTC
AAAAACTAAATCAATAGTACTAACACATAACAGTAATCACACCATGACCGTAAATGAAGA
ACTATATAGATTTTACTATGCGAGACTTTTAGAAGTCACAAGTATACTTATACTTAACAG
GGTTTAATGTATATTTTGTACCGGAAGACAACAATATTTCAAACCACCAAACGAACAAGA
AGAACCACAGGAAGATAAAACTTTGCAAGGATATACATATATCAATCTCGGCTCGAATTG
GACACCGATAAATCACACCCCATAAGTCTTATATACTGGAGCTAATTACATTCTGATTCC
TGAATCATACTCCTGATAAGTTACACTCTCACCCTGCAAGAGAACAAATCCCATACTAAT
AATCTATTTGTAACTATATTAATATATGTATACTTGAAACCCGAAATTCTTGACTAATCT
ATAACAATCAGAAAATCTTGATTTACTCTCGCAAAGAAAAACAATGGC
>AhPDF1_5
GACTATTACTTACTTAGATATCAATGTATTTGTTGTAATTAGCGTAATATCAACCTCTAT
TTATAATAAAATGATGGTGCGGTGAGTTTGTCAATCACCAATTAGACAAGTAGATAATAA
TAGTTGATACTTGATACCTATCTATAATGTTGCCTGATCTAACCTAAGTGACTGGTTTGG
CTTGAGCTAGTACTAATGTTCACTAGGGTGGCTGGATTAGTTGACTTATTGTAGATTAAC
ATGGCAAGTAGATATATCGACATATGATCTACCGATATAATAATCAAGGTCTTAGGCTCT
TAGCAAATGAGCATGATTCCCCCTCTCCATTTCCTTCATATTTTTGATACATGTTTTGGT
ATATGGTATCATAGAAGAGGATGGATAGTCACACGTGAGAATTGGCTTTCTAATTTGGTG
TTTTGGTCTTTTTACGGTTTTGAGAGTTTTTTCAGATTATATTTGATATTTATACTCTT
TAGACAATGTATCTCTCTTCTTAATAAGAATCGATTCAATACATTTCTTATGTACCCATA
TGACTAGTTCGAACACCAGGAGCGGAAGGAGGGACTTGAACCCTCAACCTTAGCCTTGGC
AAGGATCACACAATGAATTTGTTTAAAAGGGGGAAAACAAATTCAATTAACTCTCTTGCT
ATTTGTGATCAAAGACTCGAAAAAAAGGGTGAATTTTATTGCTTAGAGACAACGCTAGGG
TTTAGCTCAAGAACAATAGGGTTTTTTGTGCAAAGTTGTGGATCCCCTGAATAGGGGACA
AACCCCCCCTATTTATACATGATTAATGCGGTTTTAGGATATGTTAATCTTCTAAATTTT
CAGAGCCTTATCTTCT
>AtPDF1_5
GACTTACTACTTAGATTTGTGGTGTGGAGAAATAATGAGCGTAAGATCAACCTCTATTTA
TAATGAAATGATGGTGAAGTTAGTTTGTCATCAATTAGACAAGTAGCTAATAATGTTGCC
TGATCTAACCTAAGCGACTGGTTTGGCTTGAGCTAGTACTACATTGTTCACTAGGGCGGC
TGGTTCAGTTGACTTATTAACATGGCATGTAGATAGAATGACATATGATCTAGCGATATA
ATAATCAAGGTGTGATCAAATGAGCATGATACGCCCTCTCCATGCATGTTCTTCAGCTTT
TATTTCCATTTTTGAATTTTATCAAGTTCTGTTTTTCTTAATTTTGTAAATGATACGTAT
ATATTAGTTGATATCTTATGGGTTTGATTTCCAGTGAGCAGGGTCATATTCAATATTTTT
TAAAGACTTATCTCAAGAGAATGACCCGTAAACTGAAACTTAGCTCCATCTTCGTGTCTA
GGCAAATGCCGACTAATAACGTGTAATGATAATCTGAATCACAGTGTTGAAGGAACACAT
AAAATCAACTCAATAGTAACACATAACATTAATCACACCATGACCGTCAAAGAAGAACTT
TGTCGATTTTATTATGTGAGACTTTGAGAAGGCAAAAGTTTACTTATATACTTAACAGGG
TTTAATGTATATTTCTTACCGGAAGACAACAATATTTCATAGGTACATAACAGCTATATG
GTCACCAAAACCACCAAATGAACAACAAGAAGCAAGAATGGACATGTATCAATCTGGGTT
CAACTCTGGGCACCGATGTTTTGAGCCTGGAATCACACCCCATAACTGTTATATACTAGA
GGCTAATTACATACATATAAACACTCATTTACCTTTGGATTAGAAATCCAGGATCATACT

TTGCTTAAAGC
>AhPDF1_7
GACTATTACAACTTCGTTTTCAATGTATGTGGTGTGAGGAAATAAGCTTAGTATCAACCT
CTATTTATAGTGAAATGATGGTGAGGTTAGTCTTTTACTTTTCATCTTATACGCACACTT
TCTTCTAGTAGACAATAGAAGATAAGACGTTTGACTAGTTCATAAGTGGGTTTCTTAATC
ATATATCTGGCTGCCTAATCTACAACGTTGTTTACCAGTTTCTTAATTTTATTTTTATGT
TTTGAGAGTCTTTTAGTCATTTACCAAGTTGTTTTATAGATTTTAGGTTGCTTTGTAGAA
AAACGAGTCATTTTGGAATACCACGGTGGTATTACCACCGCTTGACTGGGGATGCAATAG
CATGTAGAAGCATAAAAAATTTAGTATTTGTTGGAAGAATTCAAATTGTTATATTGAGCT
AAAACTTTGGATACAAGATATTTGCTTGAGACAGGTTTCATTTTTAAGTAGTTTCATATC
ATTCCGATTGACGATTTAAAAGTTATAACCGTTTTTACTGAAAACATGTTTCTCGTGAA
TAGATGGAACATGCAAATAAATTTCTAACCTGGAAATATTGTTAGTTCGATCTACACACT
ACCCAAACAAATCCAATTCCAATTCGATTTTTTTTTTTGTTTTAAAACCTTAAGAGAATG
TTGATTATTTGGATATTTTGAGAAAGCATGAAAATACAAATCTCTGGTCCAACAGTGTTT
TTGGAAATATTAATATATTTAGTTTCATAATGTTTTTCATAGATTATTTTGTTTTGAATT
TTTCTATATGTGAGTAGTTTCCCAATTGGATTCAAGGTTTTCCAAAGGATTCCATTGTTT
GATTGATTGAGATATTGACTTAGGAAATTGTAATTTAGTTTCTTCATCTATGATGTTCTT
GATGTTGTAGCTAACTAGTTTCAGATCTTAGATTGTTGATGCATAAAAAATTAGTTATTT
AAAATTAAATGCAATGCAAGATGGAATTTAGATGAGTAAAACATCCATAGCCAAAGAAAA
TTGATGTTAGGATTTTTTGTGAACATATCAAACTAATTTTAATGTGTATTTTATTTCCTC
AATCAAACGATTGTTTTGAAGCTCAGTTTTTCAGACATTTTAATGGATTTTACGACATGT
AAATTATAAAATCGTTATTTGATTTTATTTTCTAATGATTTTTCTGTTTCTGCATGCAGA
AGTACTTGCAAAATTATTGATGTATAATCCTTTTAATGTATTGCTTTATCGTTTGTTTTA
ATTATATTCTCTACTTGATTTTAAAAAAATAACCAAAAACCATATTTTAGTTTAGTTCTA
CATTATTTTCGCAGACTTTTCATGGATCCCATATATATTAATTGAGAAGCATTTTTAAAA
AATAACCTTATTTTCATGTGTAATTAACAAAAATATCATGCTGACGTGTCGTTCTCACAG
AACTTCTCAATCCATTTAATTAAAAATCTAACATTTATTAAGATATTTACAGATTTTGTC
ATTAGTTTTACTTATATTCATTTTTCCACTTAATTTGTGTTTTTTATTTTACTCAATTAA
TTAAAACGAAAATATAATTAAAATATACAATTAAGATATACAAATCTATATAAAATGGAG
TGCTTAATATTTTGTATATTGATTAATATACCAAGATATATATCATAAATTATGATAAAC
ATAAACATTAATACATAAATAAAAAATTTACTAATCTTGATAATCATTAATACACCACCG
TTTATCTAATAATATAAAAATTGAACACGTTCAATATCATCAATATATTAAAATTGGTTG
ATATTCGTTTTGTCCCGTACATAGTGCGGGTGATTATCTAGTTTGATCATTAATATAGCG
ATTTATTTATGTTAAAACTTTAATTATCACTATCTCTATCTAATGTTGACCTAAGCCGCT
GCTTTGGCTTCGAGTTAGTGCTACTGCTATATCATTCACATAAGGTGGCAGTGGCTGGTT
AACTTGGATAAATTATCAAG
>AhPDF1_8aAllele12A07
GACTAGTTTTTATGCTTAGTTGTTTTCAATGTGTATATGTTTTAGCTTGAAGAAATAAGC
TTGAGATCAACCTCTATTTATACTGAAAAAAGGATGGGGGAGAAAGTGTATAATAAGTCT
ATAAGTTGGATATTTTCTATACTTTTCTTCCCACTGAAACCTCCAAAGATATTTTCGGCC
AAGTGGTAGTGATTTGTCGGCCACATCCCATTTAATAATAAGTCTATAAGTTGGATATTT
TCTAGGGCTATGCATAATGCATGATAATATTATAATTAAGTAATTACGAAAGAGAAAGAA
GAAATAAACCAAGTTTCTCCTTCTATTTATGATTAATAACGAAAATGTCATTGATTAAAA
AATTCGTTTTCCTACTTCAATATATCTACGATACTGTTCATTCCGGCGTCGATGATACTG
TTCATCACCGGATAATATTTATCAACCACATAACGACATTGCTCATCTATGTGAACAGTT
ATTTTATTTACATGTTAAATGATATATTATCGTGATAAGTTCATATAATATGTGCTTGTT
ACATTCATAGTTATCGTGTTAAACGAGTTACCATATCATACAAAATTAATATTTTATATT
AATTTAGAATTGTGAATTAATATATATTTTTAAAAAAGCTTAATTAATTACATGATAAAA
ATATTATAAACAAATTACATAGAATGATAACGATAACAATAATGATAACGCACTACACAT
ACTTGTAACGTGTTAAAATATTAAATACATCAAAATAATATTATTATTAATCTGTTAATA
CATTAAATAGTATTTTATATTGATAAATTAGGATGCCGACAAGATACCTAACAAATGATT
ATGTATTAGTTTAGATATTGATTTATTTATTTAACATGGTAAAGTAAATTGTATGTCGAA
AATTTAACATTTAACACAATACAAAAATTATAAACGTGTTATATAAAATATTAACAAGAT
ACAAAATTTAATAAAAAGATAAAGTTTTGTGTGAATTACATATTTTTTGTACCATGTTAA
TACATTACTAAACAAGAAAAACATAAAGATACAAACTTAATAAATGATTACTAAAAGATC
GTTTAATAGATTAACCAAATTGTTTACGGTCTAAACAAAGTTTTATGGGTTAACGGGTAC
GTTATCTCTCTGGGACGAAGTCCGAGA
>AhPDF1_8a
GACTAGTTTTTATGCTTAGTTGTTTTCAATGTGTATATGTTTTAGCTTGAAGAAATAAGC
TTGAGATCAACCTCTATTTATACTGAAAAAAGGATGGGGGAGAAAGTGTATAATAAGTCT
ATAAGTTGGATATTTTCTATACTTTTCTTCCCACTGAAACCTCCAAAGATATTTTCGGCC
AAGTGGTAGTGATTTGTCGGCCACATCCCATTTAATAATAAGTCTATAAGTTGGATATTT
TCTAGGGCTATGCATGATAATATTATAATTAAGTAATTATGAAAGAGAAAGAAAAAAATAA
ACCAAGTTTCTCCTTCTATTTATGATTAATAACGAAAATGTCATTGATTAAAAAAATTCGT
TTTCCTACTTCAATATATCTATGATACTGTTCATTCCGGCGTCGATGATATTGTTCATCA

CCGGATAATATTTATCAACCACATAACGACATTGCTCATCTATGTGAACAGTTATTTTAT
TTACATGTTAAATGATATATTATCGCGATAAGTTCATATAATATGTGCATGTTACATTCA
TAGTTATCGTGTTAAACGAGTTACCATATCATACAAAATTAATATTTTATATTAATTTAG
AATTGTGAATTAATATATATTTTTAAAAAAGCTTAATTAATTACATGATACAAATATTAT
AAACAAATTACATAGAATGATAACGATAACAATAATGATAACGCACTACACATACTTGTA
ACGTGTTAAAATATTAAATACATCAAAATAATATTATTATTAATCTGTTAATACATTAAA
TAGTATTTTATATTGATAAATTAGGATGCCGACAAGATACCTAACAAATGATTATGTATT
AGTTTAGATATTGATTTATTTATTTAACATGGTAAAGTAAATTGTATGTCGAAAATTTAA
CATTTAACACAATACAAAAATTATAAACGTGTTATATAAAATATTAACAAGATACAAAAT
TTAATAAAAAGATAAAGTTTTGTGTTAATTACATATTTTTTGTATCATGTTAATACATTA
CTAAACAAGAAAAACATAAAGATACAAACTTAATAAATGATTACTAAAAGATCGTTTAAT
AGATTAACCAAATTGTTTACGGGTTAACGGGTACGTTATCTCTCTGGGACGAAGTCCGAG
ACACCCTTCACCATATTTCCTGATCTCCTCTTCCAACTTTGCCAAAAAAAAGTTTAATCA
TCAAATTTTTTCATTAATGACCACTGACAGCTCGCCGGGTTCATGAACCTCCAAACTCAC
ATGATATCTTGCTTTTGTTACTTGAATGACACCACAGTTGAACCATAGACTCCGCCGGAA
TCCAAAACTGCTGTCCACCTTGGCTTTACCCGTCACGGAATTAAAAACCCACTCCATTTA
GATTTTTTTATTTCAAGTTCACTGGGATGAATTCTTGGCCGGAGCAATTTCGCCGGAAAT
ATGCAACGAAGAGAAGAGAGAGAGAGATAAATGTGAGAAATTAAAAAATTAAAAACAAAGAG
AGAGAGAATGATAGTAGAGTAATCTCCTTAGTTTTATTACTGTAGAAATAATATCTATTT
TACTCACCTAATTTTTCAATTATCTGAGCATATTCATTAATATTTTCCTATTTTCTATTG
ACTTTTCTTCCCACTGAAACCTCCAAGGAGCTACTTCACTAGCTGGCAAGTTGATAAATT
AGGTTATTTCTTCGCTTTCTTTCTTATTGATCACATAATCATCAAACTAATTAAAATCTA
GACATGTAATTAAATATTAGTCATATAGTGGATCTGAACAAATTTGCAAGAGGGTTTGGA
CTAACAAATTTGCAAGATTGAATGATTCAATAACATCCATCTTACTCCGACTTGGAATCG
TTGCCTTACTTCTCTAACTTGTTTTGCTTGGATGCTTTGTTAGTGGAATCTTCTTAAAAT
TTCTTACCAACCTTTCTTCATTTTTGTCTTGATTTAGATACCTTCGTTTCTAGTGCGTCT
CATTTGATAAACTCTTGTCT
>AhPDF1_8b
GACTAGTTTTTATGCTTAGTTGTTTTCAATGTGTATATGTTTTAGTTTGAAGAAATAAGC
TTGAGATCAACCTCTATTTAAAAGGATGGGGGAGAAAGTGTATAATAGGGGAAAAGGGTC
TATTTCCACCCCTACTATTTGGACGGTTCCGTTTCTTACCCACACAACGAGTTGGTGCTT
ATTTCAACAGTTTTCGTAACAAATTTCAAATTAACTATCCATACTCTGTGTTTATAGTTG
AATTCAACATTGACTCAAACGGACGTTAGTCAACCGTTAAAATAGTAGAGTCGGATGACA
CGGTGGCAATTCAAAAGAGGGAAAAGGTCCTATTTCCACCCTTACTATTTGAACGGTTCC
TTTTCCTACCCACACAACGAATTGGTGCCTATTTCAACAGTTTTTGGAACAAATTTCAAA
TTAACCATTTGAAATTTGTGATGACAACTTGGATATTTTCTATACTTTTCTTCCCACTGA
AACCTCCAAAGATATTTTCAGCCAAGTGGTAGTGATTTGTCGGCCACATCCCATTTAATA
ATAAGTCTATAAGTTGGATATTTTCTAGGGCTATGCATGATAATATTATAATTAAGTAAT
TATGAAAGAGAAATAAAAAATAAACCAAGTTTCTCTTTTTATTTATGATTAATAACGAAA
ATGCCATTGATTAAAAAATTCGTTTTTCTACTCCAATATATCTACATACTGTTCATTCTG
GCGTCGATGATACTGTTCATCACCGAATAATATTTATCAACCACATAACGACATTGCTCA
TCTATGTGAACAGTTATTTTATTTACATGTTAAATGATATATTATCGTGATAAGTTCATA
TAATATGTGCATGTTATATTCATAGTTATCGTGTTAAACGAGTTACCATATTATACAAAA
TTGATATTTTATATTAATTTAGAATTGTGAATTAATATATATTTTTAAAAAAGCTTAATT
AATTACATGATAAAAATATTATAAACAAATTACATAGAATGATAACGATAACAATAATGA
TAACGCACTACACATACTTGTAACGTGTTAAAATATTAAATACATCAAAATAATATTATT
ATTAATCTGTTAATACATTAAATAGTATTTTATATTGATAAATTAGGATGCCGACGAGAT
ACCTAACAAATGATTATGTATTAGTTTAGATATTGATTTATTTATTTAACATGGTAAAGT
AAATTGTATGTCGAAAATTTAACATTTAACACAATACAAAAATTATAAACGTGTTATATA
AAATATTAACAAGATACAAAATTTAATAAAAAGATAAAGTTTTGTGTGAATTACATATTT
TTTGTACCATGTTAATACATTACTAAACAAGAAAAACATAAAGATACAAACTTAATAAAT
GATTACTAAAAGATCGTTTAATAGATTAACCAAATTGTTTACGGTCTAAACAAAGTTTTA
TGGGTTAACGGGTACGTTATCTCTCTGGGAGGAAGTCCGAGT
